# From Quantum Mechanics to Coarse-Grained Models: Bridging the Gap toward Polymer Rational Design

**DOI:** 10.1021/acs.jctc.5c02092

**Published:** 2026-04-01

**Authors:** Abderrahmane Semmeq, Andoni Ugartemendia, Alessandro Mossa, Serena Coiai, Giorgia Brancolini, Giacomo Prampolini

**Affiliations:** † Istituto di Chimica dei Composti OrganoMetallici, 201844 Consiglio Nazionale delle Ricerche (ICCOM-CNR), Area della Ricerca, via G. Moruzzi 1, Pisa I-56124, Italy; ‡ Istituto Nanoscienze, 9327 CNR, S3, Via G. Campi 213/A, Modena 41125, Italy; § Polimero eta Material Aurreratuak: Fisika, Kimika eta Teknologia Saila, Kimika Fakultatea, Euskal Herriko Unibertsitatea (UPV/EHU) and Donostia International Physics Center (DIPC), M. de Lardizabal Pasealekua 3, Donostia, Euskadi 20018, Spain

## Abstract

In the rational design of novel polymers, the role of simulation methods based on classical physics is often hindered by the limited accuracy and transferability of the available models, at both the full-atomistic (FA) and coarse-grained (CG) level. Here, we introduce a first-principles-based, fully modular computational protocol for the generation of accurate and consistent FA and CG force fields, tailored on a specific material, and requiring as the sole input the chemical formula of one repeating monomeric unit of the target polymer. The proposed workflow is aimed to connect, across multiple scales, Quantum Mechanical (QM) calculations, FA and CG quantum-mechanically derived force fields (QMD-FFs), and Molecular Dynamics (MD), integrating them into a single, consistent, and reproducible framework. The protocol is tested on poly­(ethylene terephthalate) (PET), a well-known polymeric material, widely used in the packaging industry. MD simulations carried out with our FA and CG QMD-FFs are found to significantly outperform standard general-purpose models in predicting key properties such as density, glass transition temperature, as well as the intra- and supra-molecular structure. Such improvement is traced back to the accuracy of the parent QM description by controlling the adherence of the lower level models to the reference set, monitoring this flow of information at each step of the applied procedure. The performances obtained for PET confirm the reliability of a general and tunable approach, which supports systematic refinement and hence stands as a promising tool for *in silico* design of novel polymers. Subjected to further automation, the procedure could also be integrated into computational machine-learning-based high-throughput schemes, paving the way toward an efficient data-driven polymer discovery.

## Graphical abstract

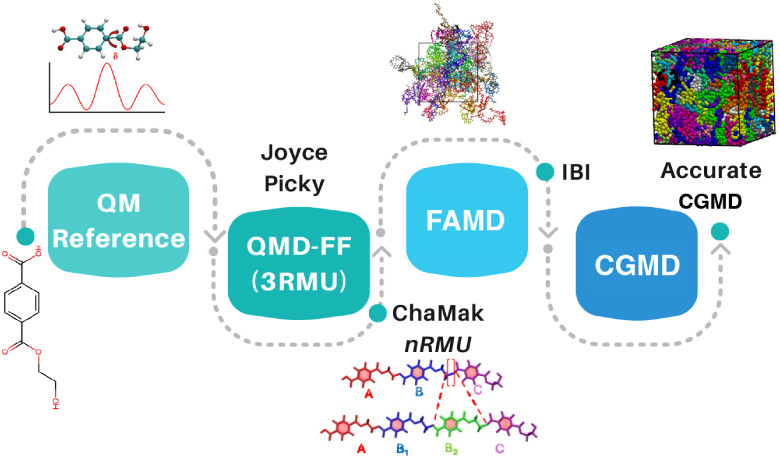

## 1 Introduction

Thanks to their structural versatility and tunable physicochemical properties, polymers are ubiquitous in modern materials science, enabling applications that range from traditional uses, such as packaging, construction, the automotive sector, and the production of technical components, to more specialized fields, including optoelectronics and biomedical technologies. Today, in response to growing environmental pollution concerns and increasingly stringent regulations regarding the life cycle of plastic materials, both research and industry are shifting toward the development and use of ecofriendly and sustainable polymers. These materials are being applied not only in conventional sectors, such as packaging,^1^ but also in advanced fields, such as nature-inspired biomedical materials,^2^ high-performance electronic devices,^3,4^ bulk heterojunction solar cells,^5^ and organic light-emitting diodes (OLEDs),^6^ to name some of the most promising emerging applications. Such a never-ending quest of novel and improved polymeric materials is nowadays flanked by the increasing concerns on their environmental impact,^7^ which bring in the challenge to design advanced polymers not only with enhanced performances but also with sustainable environmental compatibility.^8^ Both of these aspects hinge upon a deep comprehension of the structure–property relationships connecting the detailed atomistic structure of the polymer, and its possible functionalization, with the performances of the final device.

In this context, computational approaches can, on the one hand, play a crucial role in interpreting such connections, and given the availability of accurate and reliable models, bottom-up strategies could be devised for an efficient rational design of these materials. On the other hand, the wide range of length and time scales required to reliably rationalize the intricate supramolecular features characterizing polymeric materials in terms of atoms and their dynamics hampers this development and poses a significant challenge,^9–11^ which stands in simultaneously considering the supramolecular organization and the fine molecular features, including the electronic effects of functional groups and the backbone conformational flexibility. This is particularly critical in smart polymers and optoelectronic materials,^12^ where the desired performance is governed by both molecular-scale structural organization and fine electronic structure, but also more standard properties such as density, barrier, diffusion, and transport significantly depend on the accuracy of the chosen model.^13,14^

Although the most accurate approach should consist of resorting to a Quantum Mechanical (QM) framework, QM calculations are computationally prohibitive for these materials and usually limited to small systems and short simulation times (typically up to hundreds of atoms and ps). Conversely, simulation methods based on classical physics, such as full-atomistic Molecular Dynamics (FAMD) and coarse-grained Molecular Dynamics (CGMD), offer great scalability, allowing for exploration on the μs scale of wide portions of the investigated material.^15^ In particular, a long-lasting and considerable attention in polymer design has been devoted to coarse-grained models,^16–20^ which are able to disclose larger spatial and temporal scales with respect to FAMD approaches, exploiting a reduction in the number of degrees of freedom (DoFs), usually obtained by averaging out the fastest modes by grouping together multiple atoms into a single interaction site (bead). CG approaches allow in fact for accessing many dynamical properties of polymeric systems, such as chain relaxation time, diffusion, shear stress relaxation modulus, and miscibility.^20–25^ Yet, both FA and CG simulations often fail to deliver accurate and reliable predictions due to limitations in the adopted force field (FF), i.e., the model adopted to describe the potential energy surface (PES) of the target system. In fact, at the CG level, bottom-up approaches parametrize CG-FFs to match some preselected structural, dynamical, or thermodynamic reference properties, obtained with the higher resolution FA model. CG accuracy is hence not only related to the employed parametrization strategy but also remarkably dependent on the reliability of the underlying FA-FF.^26,27^ Therefore, a careful selection of the latter is crucial in any computational protocol aimed at rational design through either FA- or CGMD simulations.

The most straightforward choice is probably invoking transferability, thus assembling a FA-FF through general-purpose parameters.^28^ Nonetheless, widely used transferable FFs, such as OPLS^29^ and GAFF,^30^ are typically parametrized for biomolecules or small organic compounds, and they often underline erroneous trends in capturing key conformational and dynamic features that are critical for accurately modeling polymer systems.^31–35^ These deficiencies include inaccurate descriptions of short-range steric repulsion, conformational flexibility of polymer backbones, and π–π stacking interactions.^32^ This lack of specificity inevitably reflects on the predicted thermodynamic properties (e.g., glass transition temperature or vaporization enthalpy) and dynamical features. For instance, in the case of *n*-tetradecane, GAFF was recently found to deliver a significant overestimation of both density and heat of vaporization,^35^ which eventually led to the formation of artificial crystalline domains at room temperature. Similarly, Mohanty et al.^33^ reported that OPLS4 ^36^ performs well in predicting the self-diffusion coefficient of the ethylene glycol monomer, but its accuracy drops sharply for oligomers as short as the dimer, further deteriorating for longer chains. Such behavior upon chain elongation has also been found by Semmeq et al.^34,37^ for GAFF when tackling polyethylene glycol oligomers. Some of these inaccuracies can be corrected by resorting to a partial refinement of selected parameters, specifically carried out for the target species. Following this line, bonded and nonbonded FF terms for conjugated polymers were, for instance, obtained^38^ using capped minimal models, where the polymer backbone is optimized at the QM level and subsequently combined with OPLS-derived side-chain parameters. Similar efforts in this direction focused on recalculating partial atomic charges,^13,39^ reparametrizing torsional potentials to better capture polymer-specific conformational behavior,^40^ or applying both simultaneously.^32,41,42^ While such targeted modifications can improve the prediction of certain properties, they may worsen the performances with respect to some others, hence reducing the transferability and internal consistency of the refined FF. In particular, overfitting torsional profiles or adjusting charges to match a specific experimental observable can distort the PES defined by the FF in regions not directly constrained by the fitting data. Furthermore, such discrepancies can propagate into CG models, introducing uncontrolled biases, which can in turn undermine the CGMD predictive power.

An alternative procedure stands in retrieving the full set of FF parameters from accurate QM information, purposely computed for the species of interest.^43,44^ In this framework, the performances of quantum mechanically derived force fields (QMD-FFs) were recently validated in challenging related areas such as complex fluids,^45,46^ nanomanipulation of single-chain polymers^47,48^ and, most lately, phonon interference,^49^ hence suggesting the possibility to apply a similar strategy to tackle the simulations of polymer bulk phases. A successful integration of robust QMD-FF parametrization strategies^50^ into an automated workflow designed for computational screening protocols targeting polymer materials could not only enhance the accuracy of the FAMD predictions but even bridge the gap between the detailed and specific information retrieved at the QM level on molecular structure and the material properties predicted by the CG dynamics. As recently pointed out by Burke and Troisi,^10^ it is however crucial to carefully monitor the flux of information across the different levels of theory, whose integrity is pivotal for the success of a reliable protocol.

In this work, we present a modular and general workflow, aimed at a systematic and consistent transition of information through multiple levels of theory, ranging from QM reference data, through QMD-FF-based FAMD, up to the CG representation. To this end, the unique possibility offered by first-principles approaches to retrieve key molecular information from the sole knowledge of the molecular structure is first exploited to build an accurate atomistic QMD-FF for the chosen target species through the integration of the Joyce^35,51^ and Picky^50,52^ algorithms. The resulting parameters are thereafter employed in FAMD simulations to collect the supramolecular structural descriptors required for the subsequent CG parametrization, while preserving key physical characteristics such as chain flexibility and intra- and interchain interactions. Once validated, the here proposed workflow could bring three major advancements: i) the integration of robust and widely employed techniques such as density functional theory (DFT), QMD-FFs, FAMD, and CG modeling ensures reproducibility at all levels of theory; ii) it is in principle applicable to any polymer chain, as only the structure of the constitutional or repeating monomeric unit (**RMU**) is required as input; iii) it is highly tunable and supports systematic refinement, making it well-suited for *in silico* design and a promising tool to answer the urgent call of automated and reliable protocols in data-driven polymer discovery.^11^

For a first extensive benchmark of our protocol, we have selected a well-known and widely employed polymer, polyethylene terephthalate (PET). This choice is motivated by several reasons. First, the physicochemical properties of PET are widely known and well-characterized experimentally, which is crucial to validate the accuracy of the parametrized QMD-FF. Furthermore, abundant computational studies have been reported on PET using transferable force-fields (Tr-FFs) or partially refined ones,^53–55^ giving us the chance to thoroughly evaluate the advantages and lacks of the present approach. From a more fundamental point of view, the semiaromatic polyester structure of PET, comprising alternating ethylene glycol and terephthalate units, exhibits a combination of rigid (phenyl ring) and flexible (ethylene glycol) moieties, making it ideal to test our parametrization protocol. Finally, despite its extensive use, particularly in food and beverage packaging, textiles, and high-volume consumer products,^56^ a significant fraction of PET still ends up in landfills after single or limited use. This issue arises from multiple factors, including contamination during collection, material degradation upon reprocessing, and the limited efficiency of current recycling technologies.^57^ Moreover, the adoption of multilayer and heterogeneous packaging systems, where PET is combined with barrier polymers, adhesives, or coatings, further complicates recycling routes, often preventing a closed-loop circular process. As a consequence, PET waste contributes to environmental accumulation, microplastic formation, and long-term ecological and health concerns.^58^ In this framework, the proposed workflow may act as an enabling technology for the computational screening and rational design of next-generation linear polymers with PET-like performance but reduced environmental burden, bridging the gap between sustainability targets and advanced materials design.

## 2 Methods

### 2.1 Workflow Overview

The proposed multilevel workflow, summarized in Figure 1, is made up of several sequential modules, as outlined in the following.

**Figure 1:**
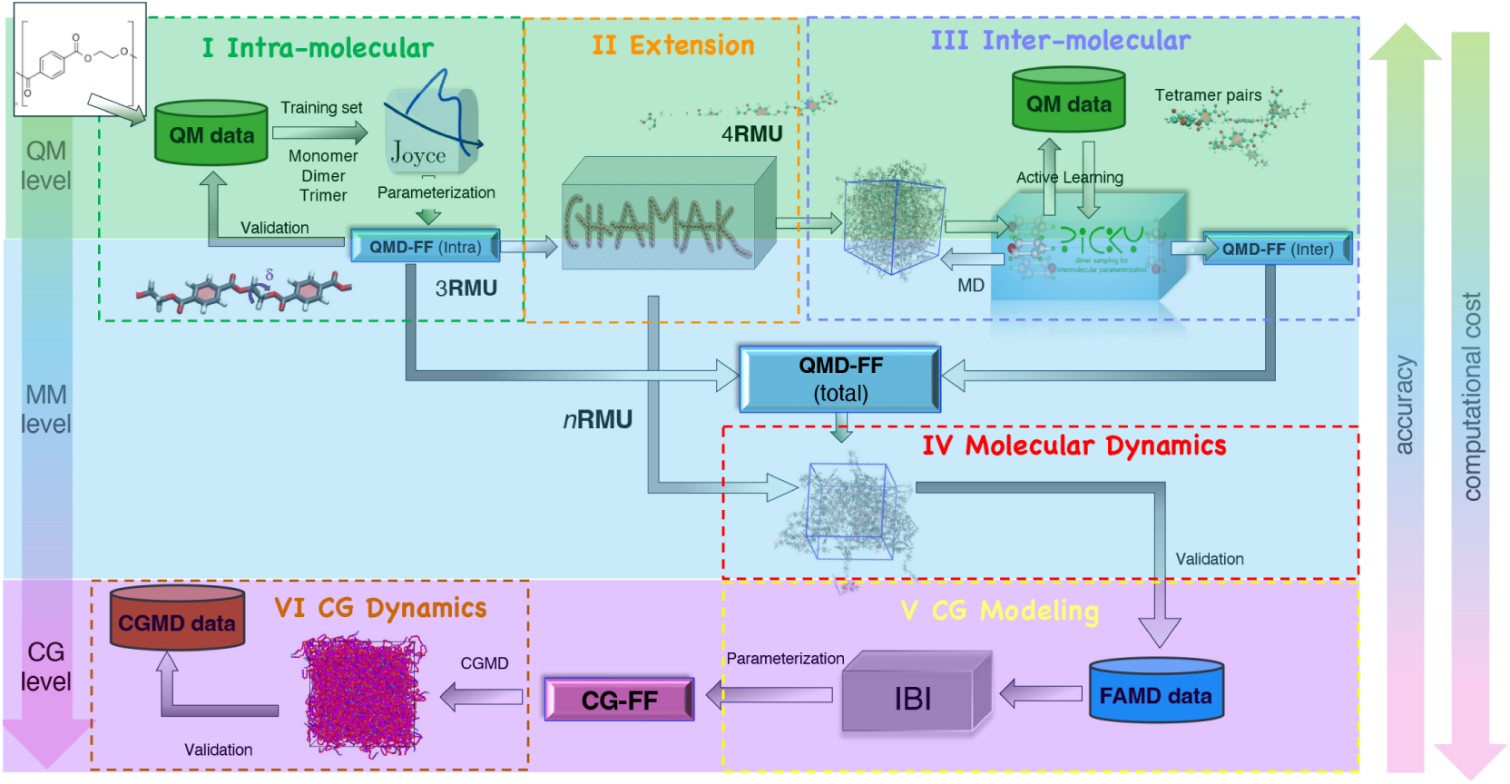
Overview of the computational workflow. The chemical formula of one repeating monomer unit (**RMU**, here ethylene terephthalate; see the inset in the top left corner) is employed to build a database containing selected molecular descriptors, specifically computed at the chosen QM level of theory (green background) for a few **RMU**s. The QM data are then employed, through modules I to III (sketched in the top panels), to derive all parameters of a PET dedicated full-atomistic QMD-FF (FA level, cyan background), suitable for polymer chains consisting of a chosen number of units (*n* **RMU**). The resulting QMD-FFs are next used in extensive MD simulations (step IV) to generate a new FA database, which serves as input for ensuing FF parametrization, carried out at the CG level (violet background) in step V of the proposed workflow. The final CG-FF is eventually employed (bottom left) in the final step VI to compute polymer properties and structural descriptors collected in the final CGMD database.

I. From the sole knowledge of the chemical structure of one **RMU** (ethylene terephthalate in this work), a number of QM descriptors is computed for the smallest representative units of a polymer (i.e., 1**RMU**, 2**RMU,** or 3**RMU**) and successively employed for the parametrization of the intramolecular terms of the QMD-FF, resorting to the Joyce protocol.^35,51^

II. The intramolecular QMD-FF obtained in the previous step for small oligomers is propagated to chains of *n* units (*n* **RMU**) through an automated protocol coded in the Chamak software (*vide infra*).
III. The QMD-FF parametrization is completed by refining the intermolecular term, which rules the interaction between different polymer chains, by applying the Picky protocol,^50,52^ where the FF parameters are again obtained by minimizing the difference with QM reference data, here purposely computed for selected chain pairs, at the same level of theory adopted in module *I*.
IV. Once the QMD-FF is assembled, FAMD at different temperatures is carried out to i) validate the FF reliability through the comparison of relevant experimental properties (e.g., density and glass transition temperature), ii) compare the results with existing transferable models to assess and control the gain in accuracy; and iii) retrieve information concerning both polymer single chains and the achieved supramolecular structure, necessary for the parametrization of the successive CG module.
V. Computationally more efficient CG-FFs are obtained through the iterative Boltzmann inversion (IBI) method, which allows for robust parametrization exploiting the FA trajectories retrieved at the previous module and offers the possibility of different mapping schemes.
VI. The best-performing CG-FF is eventually used in CGMD simulations for a final validation.

As further summarized in Table 1, each module or subunit is based on the application of a specific software, some of which is purposely coded or refined to fit the present workflow. In the same table, estimates of the average computational cost required by each module are also reported to evaluate scalability to similar or more extended systems.

**Table 1:** Summary of the Codes Employed along the Proposed Computational Workflow Sketched in Figure 1, Listed Together with Their Functions and Approximated Average Computational Cost (Wall Time), Registered for Each Module

Module	Code	Output	Wall time^a^
I.a QM database	Gaussian16 ^59^	PES descriptors	60 (<1)
$\mathrm{I.b}\;{\mathrm{QMD‐FF}}_{\mathrm{oligo}}^{\mathrm{intra}}$	Joyce^60^	QMD-FF parameters	2 (<1)
$\mathrm{II}\;{\mathrm{QMD‐FF}}_{\mathrm{poly}}^{\mathrm{intra}}$	ChaMak^61^	QMD-FF topologies	<1 (<1)
III.a QM database	Gaussian16 ^59^	IPES descriptors	280 (<1)
III.b IPES sampling	Gromacs^62^	4**RMU**’s chain pairs	4 (1)
$\mathrm{III.c}\;{\mathrm{QMD‐FF}}_{\mathrm{poly}}^{\mathrm{inter}}$	Picky^63^	Final QMD-FF for *n* **RMU**	1 (<1)
IV FAMD	Gromacs^62^	*n* **RMU** distribution functions	220 (11)
V IBI	MagiC3.0 ^64^	CG-QMD topologies	30 (10)
VI CGMD	LAMMPS^65^	polymer properties	180 (6)

a Total wall time (h) was obtained by summing all elapsed times registered for each type of calculation in the module: e.g., QM optimizations, MD runs, etc. The average time required for a single production run is also reported in parentheses.

### 2.2 QM Calculations

While most QM methods provide reasonable accuracy for all the data concerning short single chains (such as equilibrium minimum-energy geometries and relaxed energy scans of the most flexible DoFs), the noncovalent intermolecular interactions between two separate chains are highly sensitive to the choice of theory and basis set.^66,67^ Moreover, it has been shown^68^ that, even for simple liquids as pure benzene, slight differences in the reference QM description can eventually lead to significant under- or overestimations of the macroscopic properties yielded by the resulting QMD-FF. Since among other condensed-phase features, the density was reported^68^ to be particularly affected by the quality of the parent QM description, here a not careful calibration of the latter could cause artificial bias, which in turn may be inherited by the final CG model. To ensure robustness, we therefore benchmarked multiple DFT functionals with the 6-311+G­(d,p) basis set against highly accurate reference data, obtained through Coupled Cluster calculations, carried out, as described in Section S1.1 in the Supporting Information, with Single, Double, and perturbative Triple excitations, extrapolated to the Complete Basis Set limit (CCSD­(T)/CBS).^66^ As detailed in Section S1.2, our benchmarks revealed that the B3LYP functional, augmented with Grimme’s D3 dispersion correction using Becke–Johnson damping (GD3BJ),^69^ provides interaction energies that are comparable to the ones obtained at the CCSD­(T)/CBS level, yet with a remarkable computational benefit. Based on these outcomes, the DFT calculations required for Joyce and Picky parametrizations were performed at the B3LYP-D3­(BJ)/6-311+G­(d,p) level. All QM calculations were carried out with the Gaussian16 ^59^ software.

### 2.3 QMD-FF Parametrization

For a system of *N*_*c*_ polymer chains, the total energy modeled at the classical level by the QMD-FF (*E* ^QMD‑FF^) is expressed as

1 $$E^{QMD‐FF}=E_{\mathrm{intra}}^{QMD‐FF}+E_{\mathrm{inter}}^{QMD‐FF}$$

Therefore, as illustrated in Figure 1, the QMD-FF parametrization separately addresses the intramolecular $\left(E_{\mathrm{intra}}^{QMD‐FF}\right)$ and intermolecular $\left(E_{\mathrm{inter}}^{QMD‐FF}\right)$ contributions, which, respectively, govern the flexibility of each polymer chain or their interaction. Following such partition, this hierarchical approach ensures a systematic and independent control over both the intra- and intermolecular terms, enhancing the accuracy, physical consistency, and transferability of the whole QMD-FF to longer chains.^35,47,50^

#### 2.3.1 Intramolecular Term

As far as $E_{\mathrm{intra}}^{QMD‐FF}$ parametrization is concerned, we adopt a bottom-up strategy, where such transferability is first evaluated by addressing, in a stepwise manner, one, two, or three covalently bound **RMU**s, hereafter labeled 1**RMU**, 2**RMU,** and 3**RMU**, respectively. For each oligomer, all intramolecular parameters were obtained with the Joyce3.0 ^60,70^ code, following the procedure detailed in Section S2.1 of the Supporting Information. Based on the resulting 2**RMU** and 3**RMU** parameters, a custom-built algorithm is here proposed to automatically generate chains composed of *n* units (*n* **RMU**), streamlining the workflow for parameter development and enabling straightforward scaling to larger systems. The procedure has been coded in the Chain-Maker (ChaMak) software^61^ and is briefly described in the following. The ChaMak algorithm is devised for homopolymers, i.e., chains made up of *n* identical **RMU**s, whose skeleton can be schematized as A–B_1_–B_2_–···–B_(*n*‑2)_–C, where A indicates the head building block, C the tail, and B_*k*_ an internal unit. In such a framework, it is evident that 3**RMU**, which can be indicated as A–B_1_–C, already contains the main ingredients required for the description of the flexibility of longer chains. Following this scheme, ChaMak automatically inserts (*n* – 3) RMU units in the 3**RMU**’s A–B_1_–C structure, hence obtaining a longer *n* **RMU** chain, whose QMD-FF parameters are retrieved by transferring those optimized for the corresponding unit (A, B, or C) in the smaller oligomer. The resulting set, which contains information only on the interaction between First Neighboring Units (FNUs), is successively integrated by ChaMak (see section S2.1.2 for details) with additional parameters, able to describe the interaction among Far Lying Units (FLUs).

#### 2.3.2 Intermolecular Term

An accurate description of the FF intermolecular term is critical for capturing condensed-phase behavior, including packing, density, and long-range conformational properties. The parameters defining *u* ^inter^ were obtained resorting to the Picky3.0 package,^63^ following a recently proposed procedure,^50^ which integrates the original Picky algorithm^52^ with the Fragmentation Reconstruction Method (FRM).^71^ This combined approach enables the intermolecular QMD-FF parametrization of molecules of large dimensions.^50^ While further details can be found in sections S2.2 and S3.2 of the Supporting Information and in the cited references, a brief summary of the main Picky features is given in the following. The iterative procedure implemented in Picky starts with a systematic exploration of the QM interaction potential energy surface (IPES). The IPES of two interacting *n* **RMU**s is sampled by automatically selecting a diverse set of representative chain pairs, extracted from MD configurations of several *n* **RMU** chains, equilibrated at specified thermodynamic conditions. The QM interaction energies of these dimers are then computed, generating a reference database (Figure 1). The resulting QM training set is used to obtain the nonbonded QMD-FF parameters for each atom of the *n* **RMU** chain, as defined in eq (S8), namely σ^inter^, ϵ^inter^, and partial charge *q*. The resulting set of parameters is subsequently used in FAMD simulations, which produce a trajectory equilibrated on the new IPES. Additional configurations are then sampled from the latter trajectory and employed in the next Picky cycle, thus identifying additional distinct dimers and progressively enriching the training set. This QMD-FF refinement cycle (sampling → QM evaluation → parameter fitting → FAMD resampling) is repeated iteratively until convergence is achieved, namely when two consecutive cycles yield negligible differences in the IPES.

### 2.4 FAMD Simulations

Polymeric systems, consisting of a fixed number (*N*_*c*_) of *n* **RMU** chains of varied lengths (*n* = 10, 20, 50, or 100), were equilibrated at room temperature and 1 atm through FAMD simulations, following the extensive annealing protocol proposed by Lightfoot et al.^72^ The same protocol, described in more detail in Section S4.1 of the Supporting Information, was applied both to the tailored QMD-FF and to a general purpose FF (OPLS),^29^ the latter generated with the LigParGen server^73^ and hereafter labeled as Tr-FF. After equilibration, each system was then simulated for 100 ns under ambient conditions, collecting structural and thermodynamic properties such as densities, torsional distributions, and glass transition temperature (*T*_*g*_). A time step of 1 fs was used to integrate the equations of motion with the leapfrog algorithm, while bonds involving hydrogen atoms were constrained with the LINCS scheme.^74^ Short-range Coulomb and van der Waals interactions were truncated at 1.2 nm, while long-range electrostatics were treated with the smooth particle mesh Ewald method^75^ (cubic interpolation, maximum grid spacing 0.12 nm). Temperature and pressure were controlled with the velocity-rescaling thermostat^76^ and stochastic cell rescaling barostat,^77^ using coupling constants of 0.1 and 2.0 ps, respectively. All simulations were performed with the Gromacs engine.^62^

### 2.5 CG-FF Parametrization

Systematic coarse-graining included two main steps, i.e., the selection of the CG mapping scheme, which sets the degree of resolution, and the following CG-FF parametrization. For the development of a chemically specific CG model, it was essential to choose a mapping scheme that balanced the predictive accuracy and computational efficiency. On one hand, it was desirable to retain as much structural detail as possible from the FA force field to minimize information loss and preserve predictive reliability. On the other hand, because the mapping process is not unique, benchmark tests were necessary to identify the scheme that best preserved the relevant physical information while offering computational advantages.^78,79^ Hence, three different CG mapping schemes were investigated, as discussed in detail in the next sections. Each mapping scheme aimed to capture a different set of local interactions that may affect the global packing structure. In PET, material properties like density, *T*_*g*_, and oxygen permeability are governed by local interactions such as torsion (e.g., δ_5_ defined in Figure S5 and phenylene ring-flipping) and π–π stacking of the phenylene rings.^80^ For this reason, we have chosen rather finer mappings that keep key displacements between chemical moieties, such as ester–ethylene and phenylene–ester rotations, at the expense of partially losing some computational speed-up. As detailed in section S5.1 of the Supporting Information, the IBI method^16,81,82^ implemented in the MagiC3.0 package^64,83^ was used to derive the CG-FFs according to each considered scheme. To the best of our knowledge, torsional terms are not implemented in the current MagiC 3.0 version.^83^ Even though CG dihedrals usually correspond to 1–8 atomistic interactions, PET showed a relatively long soft segment, which could result in several CG torsions around the ring–glycol and ethylene glycol moieties, depending on the mapping scheme. To capture the correct chain flexibility and further address the subtle differences among the mapping schemes, an “in-house” script was used to extend the IBI parametrization to the dihedral interactions. At each iteration, additional CGMD runs were performed, from which the distributions were extracted to obtain a new set of refined CG potentials, running for each model a minimum of 5 iterations.

### 2.6 CGMD Simulations

All CGMD simulations were computed with LAMMPS software^65^ at the same thermodynamic conditions at which the CG-FFs were derived. Following the approach of Golmohammadi et al.,^84^ who validated the transferability of their CG force field derived from short PET chains (four repeat units) to longer chains, our simulations were also performed starting with short oligomer systems. The initial structures were therefore obtained by coarse-graining a well-equilibrated atomistic trajectory for the 10**RMU** system. To reduce any bias in the initial state, for each mapping scheme, 10 independent simulations were run in the NVT ensemble at 298 K using different starting configurations. The temperature was controlled with a Nosé–Hoover thermostat using a damping parameter of 100 time steps. The NVT ensemble was applied to ensure that the density of the CG model was consistent with that of the atomistic one. First, an equilibration step of 10 ns was performed, followed by a production run of 100 ns to sample the structural distributions. Such simulation lengths are sufficient to evaluate whether the developed CG models preserve the structural and thermodynamic properties. The reversible reference system propagator algorithm (rRESPA) integrator^85^ was used to speed up the bond stretching, bending, torsional, and nonbonded degrees of freedom. Namely, this algorithm decouples the slow and fast degrees of freedom by using different Liouville operators for each, making it possible to use more than one time step for integration of the different degrees of freedom. Time steps of 2 and 8 fs were used for the bonded and nonbonded interactions, respectively. A cutoff of 25 Å was employed for both nonbonded Lennard-Jones and electrostatic interactions. Beyond this cutoff, the long-range interactions were computed using the PPPM solver,^86^ with an accuracy of 1 × 10^–6^. Thermodynamic properties and snapshots were stored for analysis every 1 and 10 ps, respectively.

## 3 Results and Discussion

### 3.1 QMD-FF Parametrization

#### 3.1.1 Intramolecular Term

As outlined in Figure 1, the first module of the workflow consists in the QMD-FF parametrization of the target PET polymer through a stepwise procedure, which eventually leads to a reliable and accurate FA model of chains composed of *n* units (*n* **RMU**). The initial step involves specific atom typing, hence enabling chemically accurate differentiation of local atomic environments. Indeed, unlike general-purpose Tr-FF models, which often rely on limited or overly generic atom types, QMD-FFs employ a chemically informed labeling scheme that captures electronic and steric distinctions. The assigned atom types for the 1**RMU** monomeric unit of PET are illustrated in Figure 2a, together with a possible partition of 1**RMU** into three chemically distinct moieties: (a) the aromatic ring, (b) the carboxylate groups, and (c) the ethylene glycol-like moieties. Such fragmentation reveals three possible interactions within each **RMU**: i) steric between the COO–CH_2_–CH_2_ segment and the aromatic ring, ii) hydrogen bonding between hydroxyl and carboxylate groups, and iii) ring–ring interactions. As displayed in Figure 2b, all of these specific interactions within each **RMU** are fully transferable to larger oligomers. As an example, Figure 2c displays the partition of the 3**RMU** trimer into three units (A, B, and C), respectively, referring to the head, central, and tail units. Following this scheme, we retain the same types shown for the 1**RMU** parametrization for all *n* **RMU** polymer chains, with an additional designation “a” for atoms belonging to the head unit (A), “b­(_*n*_)” for the central (B) unit(s), and “c” for those in the tail unit (C), to account for positional asymmetry in going from 2**RMU** to 3**RMU**.

**Figure 2:**
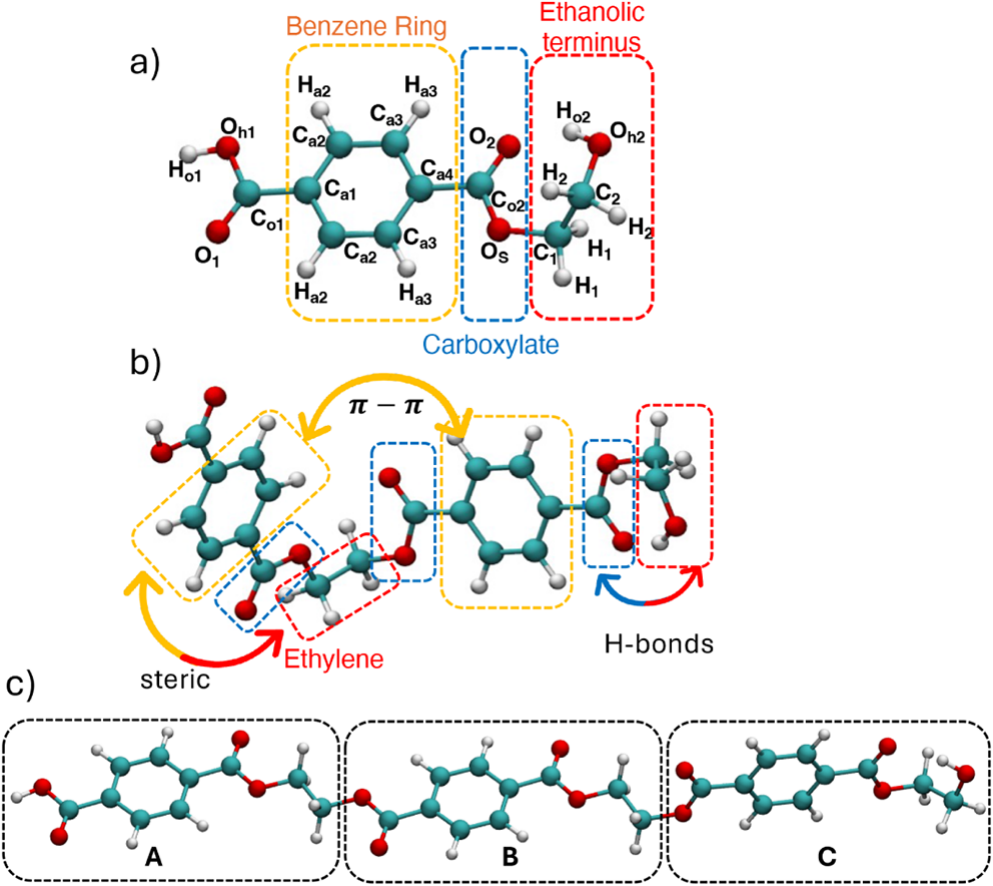
QM equilibrium geometries of PET’s monomeric unit (1**RMU**), dimeric (2**RMU**) and trimeric (3**RMU**) units, and atom typing. a) 1**RMU**: the employed QMD-FF atom labels are displayed with the definition of the main building blocks; b) 2**RMU**: building blocks-based fragmentation scheme used to define the intramolecular LJ interactions between moieties (colored arrows) within the dimer; c) 3**RMU**: PET chain fragmentation scheme into **RMU**s.

Once all specific atom types have been assigned to each of the considered oligomers, separate intramolecular parameters were obtained, with the parametrization carried out for each *n* **RMU** chain (*n* = 1–3) as described in the Supporting Information. Panels a–g of Figure 3 show the equilibrium geometries obtained at QM and QMD-FF levels for the three oligomers. The FF-predicted structures align very well with the reference QM geometries, delivering a root mean squared displacement (RMSD) of 0.2, 0.4, and 0.4 Å for 1**RMU**, 2**RMU,** and 3**RMU**, respectively. A further validation was carried out to verify how faithfully the QMD-FF captures the features of the reference QM PES in a local harmonic approximation (LHA), hence ensuring a correct dynamics near the equilibrium. Panels b–h of the same figure indicate very good adherence to the parent QM description, achieved in both the vibrational frequencies (see correlation plots in bottom panels) and their subtending normal modes (histograms, top panels), confirming the accuracy of the harmonic terms of the QMD-FF (see Equation (S5) in the Supporting Information) in describing the QM features in LHA. Interestingly, a slightly worse agreement is registered in the frequency range between 300 and 1200 cm^–1^, corresponding to normal modes delocalized over several internal coordinates, e.g., stiff dihedrals and bending angles. Notwithstanding, a better description of such modes could be achieved by introducing explicit coupling functions in the adopted FF model;^87^ the simpler standard diagonal FF expression (S5) was adopted to enhance both code portability and computational performances. Upon chain elongation, in an even lower frequency range (<200 cm^–1^) corresponding to “soft” modes, the overlap between QM normal modes and those obtained from the QMD-FF also worsens, as such coordinates may be subjected to large amplitude displacements and hence should not be treated within LHA.

**Figure 3:**
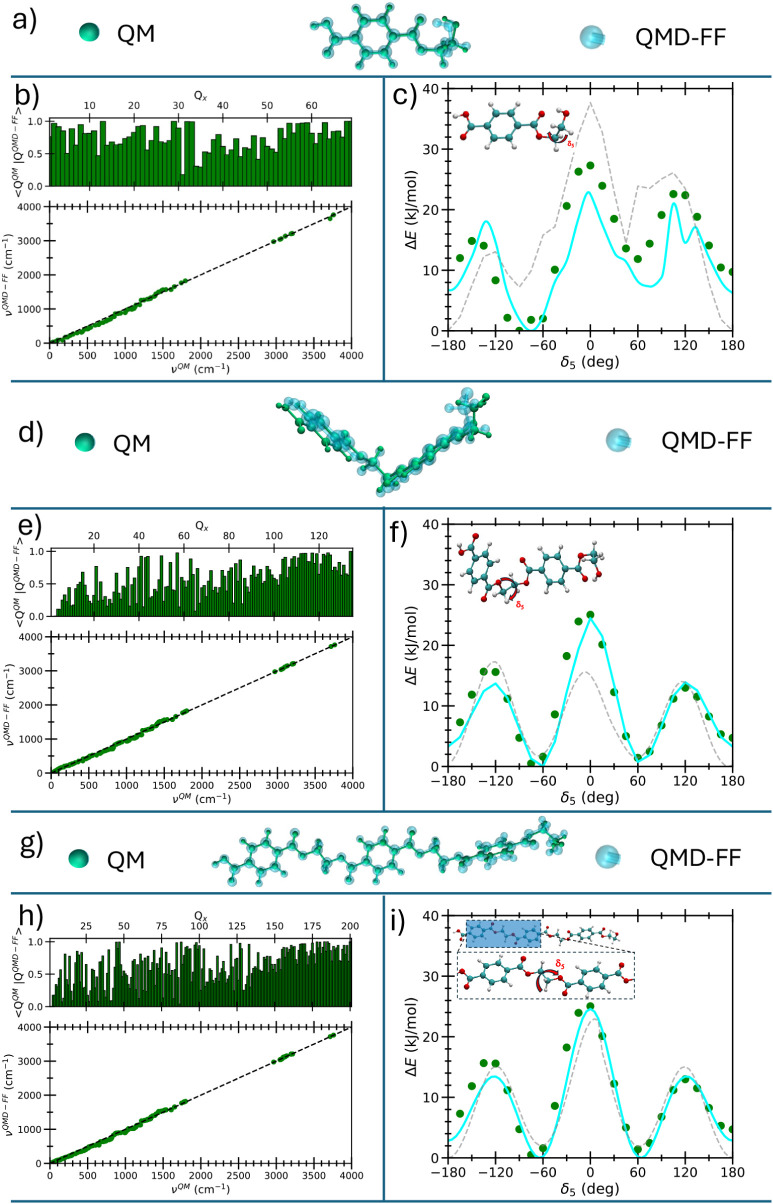
Results of the intramolecular QMD-FF parametrizations displayed for a–c) 1**RMU**; d–f) 2**RMU**; g–i) 3**RMU**. a), d), and g) Overlap of the optimized structures at QM (inner dark green spheres) and QMD-FF (outer transparent cyan spheres) levels. b), e), and h) Top: overlap of QM and QMD-FF normal modes; bottom: correlation plot between QM and QMD-FF vibrational frequencies. c), f), and i) Relaxed energy profiles obtained upon δ_5_ rotation (see insets) at the QM level (solid green circles) and with the Joyce-parametrized QMD-FF (solid cyan lines). Similar scans computed through Tr-FF (OPLS,^73^ dashed gray lines) are also displayed for comparison.

According to the Joyce^35,51^ procedure, soft coordinates are usually identified as the dihedral angles ruling rotation around σ-bonds. As evidenced in Figure S5, 1**RMU** includes five internal torsions, denoted respectively as δ_1_ to δ_5_, plus two terminal −OH torsions. In 2**RMU**, the same torsions could be defined for the A head unit, their equivalent counterparts for the tail unit B labeled $\delta_{1}^{\prime }$ to $\delta_{5}^{\prime }$ , and the dihedrals governing the linkage between the A and B units through the internal rotation in the ethylene/carboxylate bridge denoted as δ_*AC*1_ and δ_*AC*2_. The same labeling is straightforwardly transferred to 3**RMU**, where equivalent dihedrals can be defined for each **RMU** forming the trimer, i.e., δ_*AB*1_, δ_*AB*2_ and δ_*BC*1_, δ_*BC*2_ linking AB and BC units, respectively. It is important to stress that this dihedral labeling strategy allows for maintaining consistency in defining such torsions across different chain extensions, hence prompting a simple transferable procedure for the QMD-FF parameters to larger *n* **RMU**’s. Panels c and f of Figure 3 present the comparison between the QMD-FF energy profiles and the QM training data, computed along the δ_5_ rotation for 1**RMU** and 2**RMU**. The similarity of the 1**RMU** and 2**RMU** QM profiles, found for δ_5_ and for most of the considered dihedrals (see Section S3.1.1 in the Supporting Information), prompted us to transfer the QMD-FF parameters obtained for the flexible coordinates of the dimer to the longer chains, hence avoiding the computational burden associated with the systematic QM torsional scans, limiting the calculations to some selected “control” scans, carried out for the 3**RMU** chain and also shown for validation purposes in Figure 3i. For all three oligomers, QMD-FF profiles are in excellent agreement with the QM reference, demonstrating that the FA model also accurately captures the anharmonic rotational features, essential for describing large-amplitude motions and conformational transitions toward PES regions far from the minimum energy geometry. In the same panels, the comparison with the torsional energy profiles computed with the general-purpose Tr-FF clearly indicates that the best agreement with the QM description is yielded by QMD-FF, even for the data outside the training set. In fact, notwithstanding Tr-FF is able to predict with rather good accuracy many of the considered scans, it misrepresents δ_5_, which drives the flexibility of the ethylene segment, significantly impacts the overall shape of the chain, and hence plays a crucial role in determining important PET’s conformational properties.^88,89^ Indeed, the Tr-FF description appears to predict the *trans*-configuration to be more stable than the *cis*-one, which could bias the conformational dynamics of longer *n* **RMU** chains, predicting overestimated elongations.

Following the main workflow reported in Figure 1, the next module of our protocol toward realistic simulations of polymeric materials requires significantly longer *n* **RMU** chains whose structures and FF parameters were automatically obtained through the ChaMak code, based on the QMD-FFs parametrized for 3**RMU**. In fact, the latter oligomer already contains all the essential building blocks required for the description of longer chains, i.e., a head unit A, a central moiety B, and a tail C (see Figure 2c). According to this scheme, four additional *n* **RMU** were assembled, exploiting the ChaMak code, systematically inserting and replicating B building blocks in an A–B_1_–B_2_–···–B_(*n*‑2)_–C structured chain of different lengths, thus obtaining intramolecular QMD-FFs suitable for 10**RMU**, 20**RMU**, 50**RMU,** and 100**RMU** polymers. Further details can be found in Section S2.1.2 of the Supporting Information.

#### 3.1.2 Intermolecular Term

The next step of the workflow requires modeling the interactions between two separate polymer chains, thus completing the QMD-FF with the intermolecular term. To this end, the Picky procedure^50^ was applied to a model *n* **RMU** chain, made up of four units (4**RMU**). In fact, this oligomer is large enough to capture key chain–chain interactions, including π–π stacking, hydrogen bonding, and long-range conformational effects, yet small enough to remain computationally feasible for sampling the chain–chain IPES through QM calculations. The molecular structure and intramolecular QMD-FF parameters, obtained with the ChaMak code as sketched in Figure 4a, were first completed with intermolecular parameters transferred from the OPLS database.^73^ The resulting “hybrid” FF is initially employed in MD runs to equilibrate a system of 216 4**RMU** chains at 1 atm and 350 K, which yielded the starting configuration to initiate the Picky cycles. A total of four cycles were required to achieve convergence, the difference between the IPES of the two last cycles being less than 1 kJ/mol. The final training set included 350 distinct chain pairs, extracted along the Picky cycles as shown in Figure 4b, whose interaction energies, computed at the QM level, range from −160 to +10 kJ/mol. Figure 4c confirms the broad and effective sampling of the IPES in terms of chain elongation, chain–chain distance, and reciprocal orientation of the selected chain pairs. Panel d of the same figure compares the correlation plot of the chain–chain interaction energies computed at the QM or FF level, with either OPLS or QMD-FF. The latter clearly outperforms the former, as evidenced by the deviation with respect to the QM values, which is reduced from 22.1 to 3.1 kJ/mol. Further details and the complete intermolecular QMD-FF parameter set are available in the Supporting Information.

**Figure 4:**
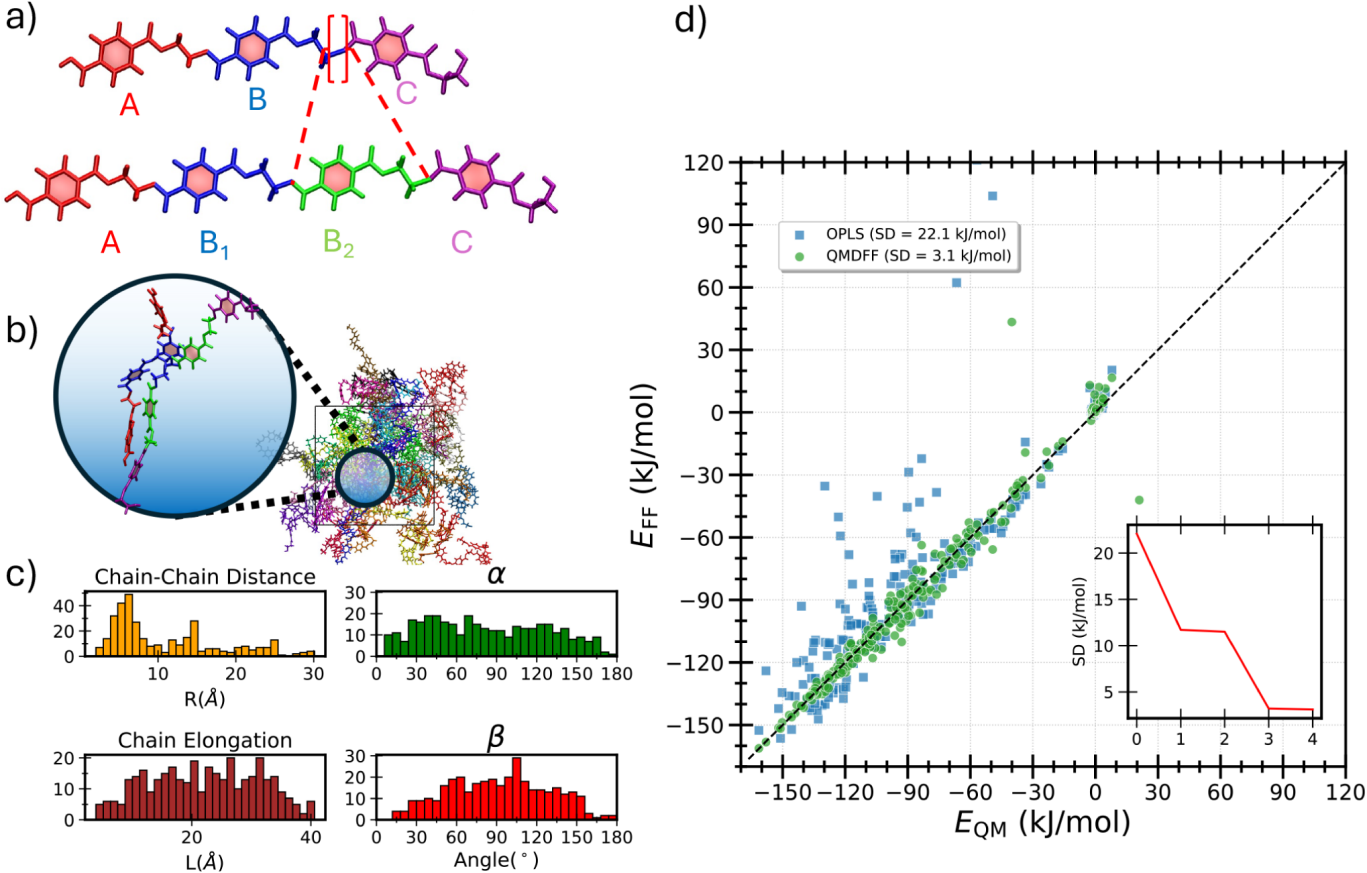
Summary of the Picky procedure applied for the parametrization of the intermolecular QMD-FF term. a) 4**RMU** chain created by ChaMak by inserting a B_2_ unit (green building block) within the 3**RMU** skeleton; b) example of a pair of 4**RMU** chains extracted from an equilibrated configuration of the FAMD trajectory; c) distributions achieved along the Picky sampling: chain–chain distance (orange), chain elongation (brown), α and β orientational angles (green and red, respectively; see Figure S1 for definition); d) correlation plot of the QM (blue) and QMD-FF (green) interaction energies of the sampled chain pairs and convergence (inset) of the Picky cycles.

### 3.2 FAMD on *n* **RMU** Polymer Chains

Four complete QMD-FFs, suitable for FAMD simulations of 10**RMU**, 20**RMU**, 50**RMU,** and 100**RMU** polymer chains, were built by joining the intra- and intermolecular terms, parametrized as described in the previous sections. For each FA model, a system composed of a fixed number (*N*_*c*_) of *n* **RMU** chains (*n* = 10, 20, 50, or 100) was prepared and equilibrated under ambient conditions, as described in Section 2. Several FAMD runs were thereafter carried out at ambient pressure and varied temperatures. To assess the QMD-FF performance, we chose to first analyze the bulk density of the resulting amorphous condensed phase, the glass transition temperature (*T*_*g*_), and the chain conformational behavior, comparing them with the experimental data available for PET and with the same properties computed adopting a general purpose Tr-FF.^73^ Table 2 reports, for all four investigated *n* **RMU** models, the average density observed with FAMD simulations carried out with either Tr-FF or QMD-FF. According to our computations, at room temperature, the system density obtained with Tr-FF is around 1256 kg/m^3^ for shorter chains, while it decreases slightly below 1250 kg/m^3^ for the 100**RMU** system. Conversely, QMD-FF predicts in all cases a density around 1373 kg/m^3^, hence decreasing to ∼2% the error with respect to the experimental range^90,91^ for amorphous PET (1335–1345 kg/m^3^). Interestingly, the density variation upon chain elongation is minimal in both models, thus suggesting that the basic physics leading to the condensed phase structure is already caught by the smaller model. For this reason, the following discussion will be limited, for simplicity, to the 10**RMU** system, whereas further details on the similar results obtained for the longer chains can be found in the Supporting Information. A second validation test carried out to evaluate QMD-FF reliability and accuracy concerns the prediction of the glass transition temperature (*T*_*g*_). This property is indeed crucial in polymer science, as it plays a central role in determining thermal, mechanical, and processing properties of soft-matter-based materials, which demand dimensional stability, rigidity, or resistance to deformation at elevated temperatures. To compute *T*_*g*_, several FAMD runs were carried out as described in Section S4.2 of the Supporting Information, varying the temperature at 1 atm in the 50 to 550 K range and recording the system density under the imposed thermodynamic conditions. The resulting density–temperature curves, shown in Figure 5 for the 10**RMU** system, exhibit two distinct linear regions with different slopes: one corresponding to the glassy state at lower temperatures, where the material is rigid and the density changes slowly with temperature, and another corresponding to the rubbery or liquid-like state at higher temperatures, characterized by a steeper slope due to increased molecular mobility. In this framework, *T*_*g*_ can be defined as the intersection point of these two regimes and therefore estimated, as displayed in Figure 5, through a bilinear fitting of the density–temperature (ρ–T) curves obtained from the FAMD runs. Applying this approach, *T*_*g*_ delivered by Tr-FF for 100**RMU** is slightly higher than 445 K, thus severely overestimating the experimental range (343–349 K).^92^ Conversely, QMD-FF predicts a *T*_*g*_ of ∼365 K, thus significantly reducing the gap with respect to experimental measures, from the ∼30% deviation obtained with the Tr-FF to less than 6%.

**Table 2:** Comparison between the *n* **RMU** Densities (kg m^–3^) Computed with FAMD Runs at 1 Atm and 298 K with the Tr-FF and QMD-FF Models^a^

*n* **RMU**	*N*_*c*_	Tr-FF	QMD-FF
10	36	1256	1373
20	18	1256	1371
50	8	1257	1373
100	4	1249	1374
		exp: 1335–1345 ^90,91^	

a The experimental value measured for PET under the same thermodynamic conditions is reported at the bottom for comparison.

**Figure 5:**
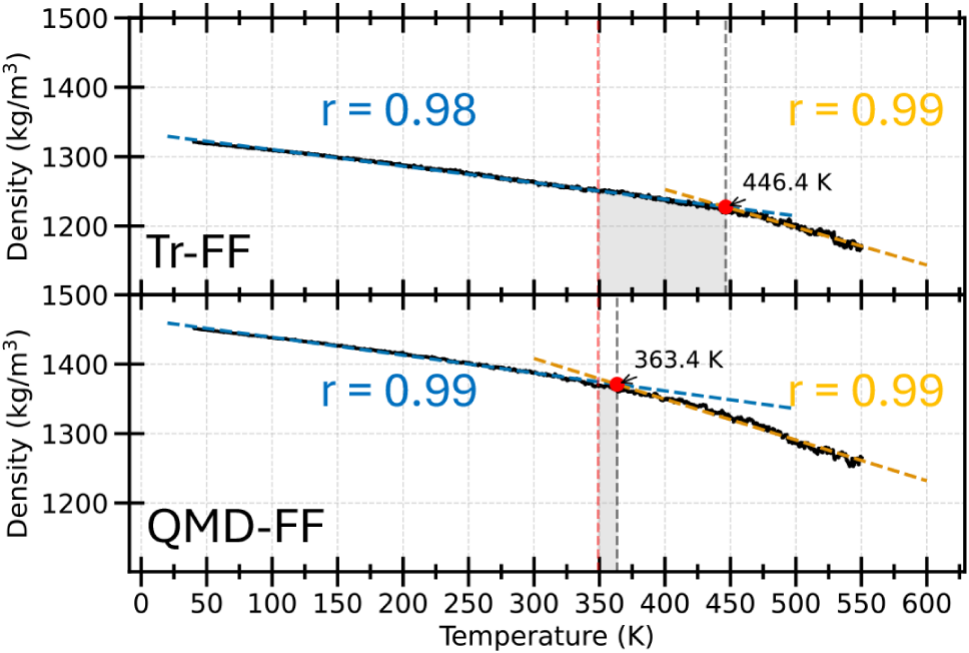
ρ–T curves compare both Tr-FF (top) and QMD-FF (bottom). The ρ­(T) curves computed along the MD trajectories are displayed with black solid lines, whereas their linear fits are shown with blue and orange dashed lines with the corresponding correlation coefficients r. In both panels, the experimental and computed *T_g_*’s are evidenced with red and black vertical dashed lines, while shaded areas highlight the error.

The different performances delivered by the two investigated models in assessing both PET’s density and *T*_*g*_ might stem from the different descriptions of the polymer conformational landscape given by the two FFs. In fact, a more flexible polymer chain packs more efficiently and requires lower temperatures to undergo the glass transition. Since the better agreement found with the experiment for QMD-FF results from a density increase and a *T*_*g*_ decrease with respect to the general purpose description, a larger degree of entanglement and a more rich conformational variety within the chains should be expected with respect to Tr-FF. Indeed, FT-IR studies^92,93^ aimed to elucidate the relaxation behavior of PET at various temperatures in relation to its conformational energy landscape revealed pronounced temperature-dependent variations in the conformational properties of PET. More specifically, several authors^88,89,92,93^ report an abrupt change in the *trans*–*gauche* population of the O–CH_2_–CH_2_–O dihedral (herein denoted as δ_5_) occurring at *T*_g_. This observation demonstrates that the conformational and relaxation dynamics evolve concomitantly, thereby demonstrating a direct coupling between the glass transition and the intramolecular flexibility. To verify this hypothesis, we exploited the 10**RMU** FAMD trajectories to gain a deeper insight into the conformational dynamics within each chain, concretely by i) estimating chain elongation by considering the gyration radius (*R*_*g*_; see Figure 6a for definition); ii) monitoring the average population of the most flexible dihedrals. The first four panels of Figure 6 compare *R*_*g*_’s distributions obtained at ambient conditions along the Tr-FF and QMD-FF trajectories. Significant differences appear between the two models, with more grooved conformations populated by QMD-FF (hence leading to the registered density increase), while more elongated, thus less packed, chains are found with Tr-FF. This is illustrated by the representative MD snapshots in panels b) and d). To shed light onto the origin of such differences, we display in panel e the population distribution of two selected chain dihedrals, namely, δ_2_ and δ_4_, evidenced in the insets. Noticeably, in contrast with QMD-FF, which predicts a non-negligible δ_2_ population between 0° and 180°, Tr-FF-based dynamics miss the occurrence of such nonplanar terephthalate conformations, hence resulting in a stiffer backbone, which in turn leads to more elongated (larger *R*_*g*_) and less grooved chain configurations. Also, the δ_4_ population obtained with the Tr-FF exhibits shifted peaks with respect to the refined QMD-FF and, more importantly, a significant increase in the *trans* conformation, again pointing to more elongated segments. A final validation of the quality of the parametrized QMD-FF comes from Table 3, where the conformational distribution of the O–CH_2_–CH_2_–O dihedral (δ_5_) is analyzed in terms of *trans*/*gauche* percentage, delivered by the two models or measured by experiment.^88^ By considering the results obtained for different chain lengths, in contrast with the considered general purpose description, the QMD-FF model appears to be more stable upon chain elongation. More importantly, the populations registered by QMD-FF for the two conformers quantitatively agree with the experimental range of values, again correcting Tr-FF, which instead predicts a slightly overestimated *trans* population, and confirming that both the chain conformational dynamics and the polymer supramolecular structure are accurately described by the QMD model.

**Figure 6:**
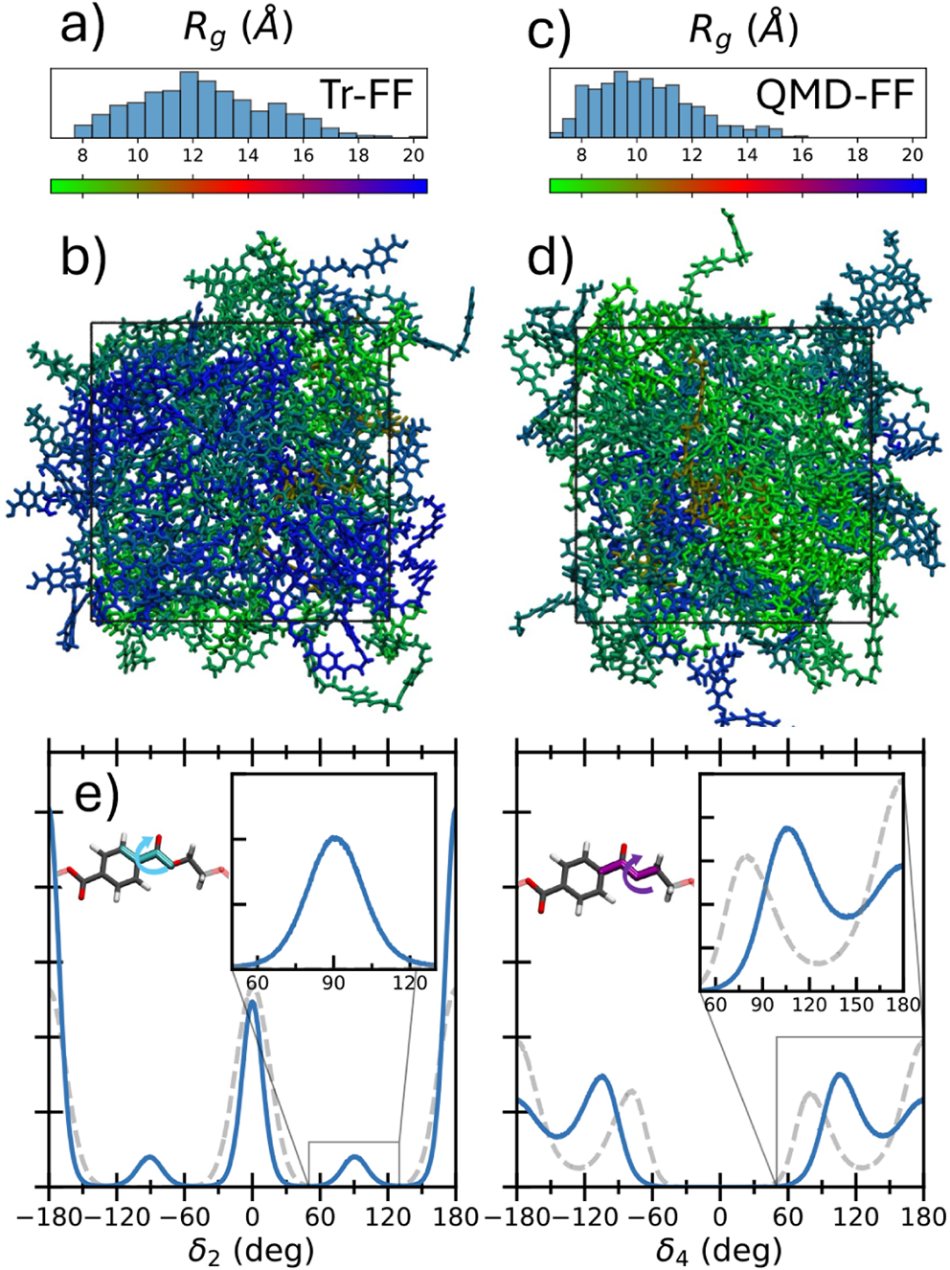
Distributions of the radius of gyration (*R_g_*) and representative FAMD snapshots of polymer chains colored according to their respective *R_g_* values for Tr-FF (a, b) and QMD-FF (c, d). Panel (e) shows the distributions of selected flexible-chain dihedral angles (δ_2_, left and δ_4_, right), obtained with QMD-FF (blue) and Tr-FF (gray).

**Table 3:** Comparison between the *n* **RMU** δ_5_ Populations (in %) Computed with FAMD Runs at 1 atm and 298 K with the Tr-FF and QMD-FF Models^a^

	*trans*		*gauche*	
*n* **RMU**	Tr-FF	QMD-FF	Tr-FF	QMD-FF
4	42	8	58	92
10	17	10	83	90
20	9	9	91	91
50	10	9	90	91
100	17	8	83	92
exp.^88,89^	5–11		89–95	

a The experimental value measured for PET in the same thermodynamic conditions is reported at the bottom for comparison.

### 3.3 CGMD of *n* **RMU** Polymer Chains

#### 3.3.1 CG-FF Parametrization

Module *V* of the workflow sketched in Figure 1 concerns coarse-graining. As discussed in Section 2, the use of well-validated reference FAMD trajectories is essential since any inaccuracy present in the atomistic FF is inherited by the CG model. It is therefore interesting to examine how much of the structural details contained in the FA description is passed to the lower level CG-FF. To this end, we assess the ability of three different CG models, sketched in Figure 7, to reproduce local structural properties within each **RMU** as described by the parent FA model. The distributions of bond lengths, bond angles, and dihedral angles, as well as intramolecular conformational populations, describe in fact the internal geometry and flexibility of each unit within the polymer chain and thus serve as the fundamental structural basis for developing the CG force field. For representing one **RMU**, each of the considered CG mappings employs a different combination of beads, summarized in Figure 7c for schemes A, B, and C, respectively. Scheme A employs the smaller number of beads (4) and hence constitutes our reference.^84^ In this mapping, the phenylene ring is represented by a single CG bead, the carboxyl moieties on both sides of the ring are each mapped into separate beads, and the ethylene moiety forms the fourth bead (see the right panel of Figure 7c). Scheme B is obtained by further partitioning the CG site assigned to the aromatic moiety in scheme A into three smaller beads, resulting in a total of six units. In scheme C, composed of five beads, the ethylene-glycol moiety is instead divided into two distinct beads, namely, O–CH_2_ and C=O. Each mapping scheme is designed to emphasize different local structural features, which may influence the overall packing behavior. For instance, approximating the phenylene rings as spherical beads may allow them to slide more easily, thus reducing the extent of π–π stacking and leading to an overestimation of chain mobility. Scheme B, by introducing a more detailed representation of the aromatic moiety, might partially recover a more realistic description of the ring planarity. Similarly, in scheme C, the explicit treatment of the rotation around the δ_5_ dihedral of the ethylene glycol segment could provide an additional degree of flexibility in the chain backbone.

**Figure 7:**
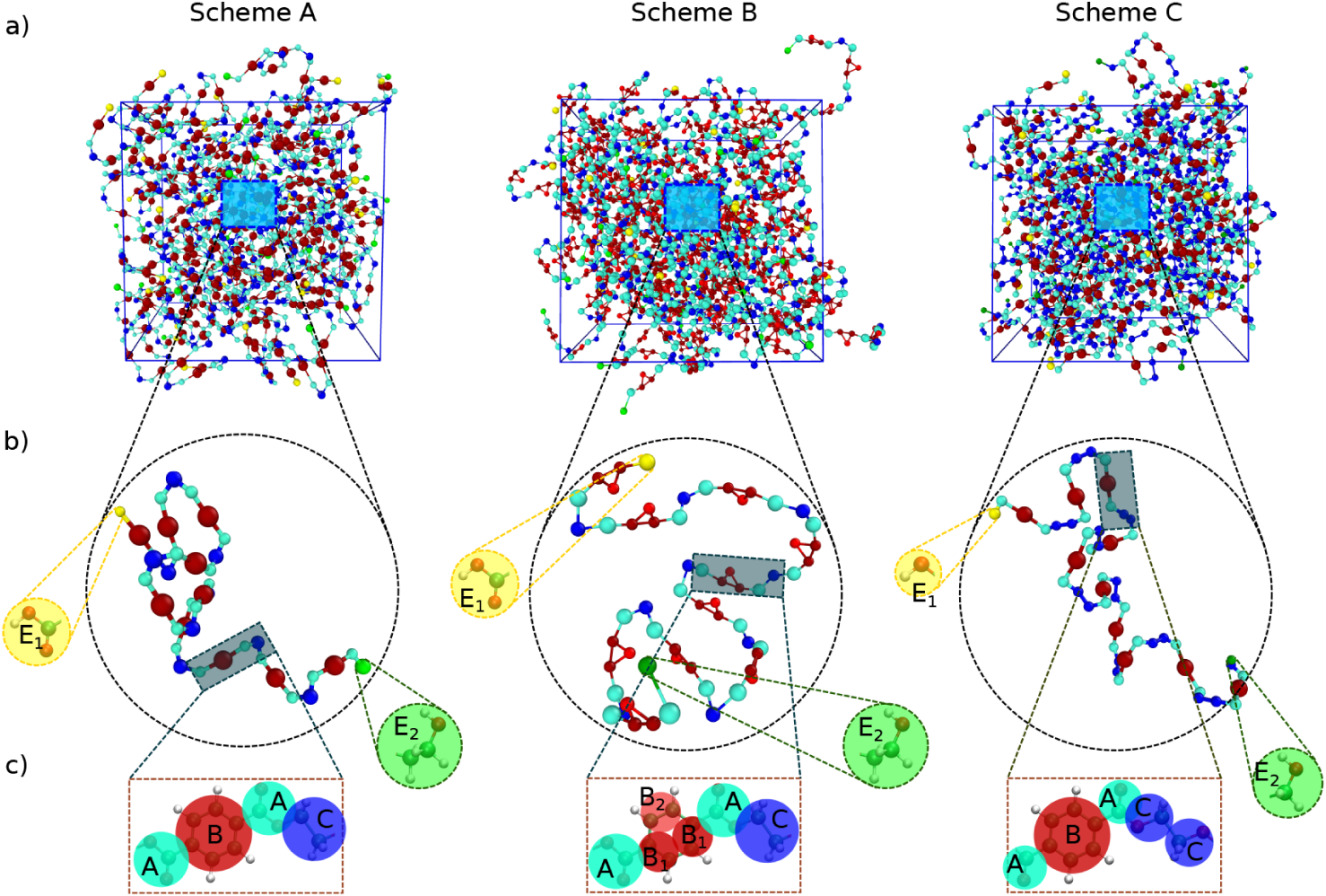
Coarse-grained (CG) mapping schemes A to C adopted in this work: a) snapshots of the full simulation box, b) snapshots of a single chain, and c) detailed chemical structure of CG-mapped beads of the **RMU** as well as the end groups.

Among the three mapping schemes, Scheme A is the coarsest and allows the largest time step (5 fs), resulting in the greatest computational speed-up and making it the most efficient choice for long-time-scale simulations. Scheme C permits a time step of 3 fs, whereas Scheme B is limited to 2 fs due to the presence of stiffer bonded potentials involving aromatic beads, which constrain the achievable acceleration. The derivation of the CG parameters was performed using as reference two different atomistic force fields: for each of the three mapping schemes, two independent CG parametrizations were carried out based on the distribution functions computed from FAMD trajectories generated with either the Tr-FF or the QMD-FF model, resulting in the six distinct CG-FFs summarized in Table 4. Since Scheme A represents the coarsest and most computationally efficient model, with improved numerical stability, we focus our detailed discussion on the CG-QMD-A and CG-Tr-A models, while the results for mappings B and C are reported in Section S6.2.

**Table 4:** CG Models Parametrized in the Present Work over the FA Trajectories Obtained with either Tr-FF or the QMD-FF

reference FA	CG mapping	number of beads	label
Tr-FF	A	4	CG-Tr-A
	B	6	CG-Tr-B
	C	5	CG-Tr-C
QMD-FF	A	4	CG-QMD-A
	B	6	CG-QMD-B
	C	5	CG-QMD-C

According to CG parametrization scheme A, 4 bonds, 5 angles, and 4 dihedrals (including E_1_ and E_2_ edges) are required to describe the chain’s conformational flexibility. The distributions of bond stretching, angle bending, and dihedral rotation along these CG coordinates were obtained from the two FAMD trajectories and used as reference for the CG-Tr-A and CG-QMD-A parametrizations, together with the radial distribution functions between the beads' centers. Such bonded and nonbonded conformational distributions contain all the information about the local arrangement of the CG beads relative to the others. Figure 8 shows the population distributions for selected CG stretching and bending CG coordinates computed along the two FAMD trajectories, the effective potentials obtained by their Boltzmann inversion, and the same functions retrieved with the resulting CG-Tr-A and CG-QMD-A models. Overall, a very good agreement is observed between the CG models and their atomistic reference curves. Some of the resulting profiles, e.g., P_*bond*_(*l*_*AB*_) and P_*angle*_(θ_*ABA*_), exhibit sharp peaks and well-defined Gaussian shapes, meaning that the underlying potentials can indeed be modeled as harmonic springs. Such stiff modes are negligibly correlated to the other DoFs, but they are efficient to preserve important local structural features. For instance, the single peak at 180° found for the θ_*ABA*_ angle indicates that the carbonyl groups tend to remain nearly coplanar, reflecting the rigidity of the ester linkage and the planarity of the terephthalate backbone. Conversely, P_*bond*_(*l*_*AC*_) and P_*angle*_(θ_*ACA*_) show a broader shape, characterized by multiple maxima and shoulders. This broader shape arises because, during coarse-graining, several distinct atomistic dihedral conformations are effectively projected onto a single CG bond or angle. It is hence interesting to compare the P_*bond*_(*l*_*AC*_), P_*angle*_(θ_*ACA*_), and P_*angle*_(θ_*BAC*_) profiles (see Section S6.2), obtained with either the CG-Tr-A or CG-QMD-A model, because these distributions provide insights into the atomistic δ_*OCCO*_ (δ_5_) dihedral. At the FA level, changes in the δ_5_ population between *gauche* and *trans* conformations are known to affect the CG bond distances and angles between the corresponding beads.^84^ The resulting differences between the CG-Tr-A and CG-QMD-A models therefore arise from varied relative populations of the *gauche* and *trans* conformations at the atomistic scale, which in turn descend from the different adherence to the QM torsional profile (see Figure 3) delivered by the two FA-FFs.

**Figure 8:**
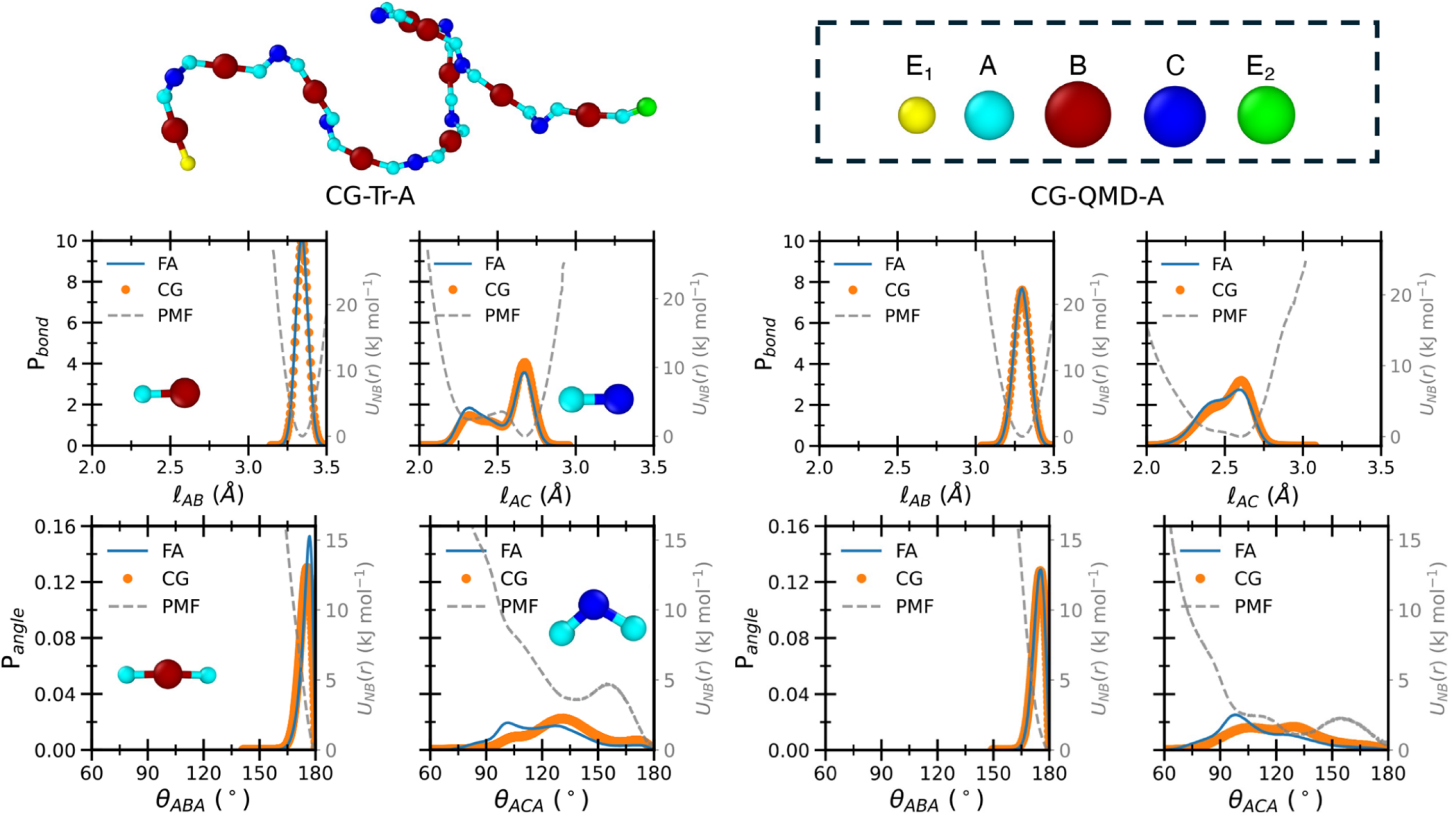
Probability distribution functions computed within CG-Tr-A (left) or CG-QMD-A (right) schemes for selected bond stretching (P*_bond_* (*l*), top row) and angle bending (P*_angle_* (θ), bottom row) coordinates (see insets) of the 10**RMU** system at FA (blue lines) and CG levels (orange circles), using mapping scheme A, shown in the top right corner. In all panels, the corresponding CG effective potentials are displayed with gray dashed lines.

As explained in detail in Section S6.2 of the Supporting Information, the local structure of different chain regions is primarily determined by the stretching and bending coordinates between neighboring beads, whereas the overall shape and conformational dynamics of the entire chain are mainly controlled by the CG dihedral angles. By looking at the top panels of Figure 9, it is apparent that the behavior of these coordinates also reflects the differences between the Tr-FF and QMD-FF atomistic trajectories. For example, in line with the results previously reported by Golmohammadi et al.,^84^ CG-Tr-A adopts significantly nonplanar conformations, exhibiting two maxima at ±60° for all dihedrals except ϕ_*ACAB*_ (see Section S6.2.3 in the Supporting Information for additional details). Such behavior can be traced back to a less accurate description of the chain conformations achieved at the FA level with the parent general-purpose description. As displayed in Figure 3, the QMD-FF corrects the Tr-FF profiles, and it is pivotal that the CG modeling proves to be able to encode this improvement. As a matter of fact, all torsional distributions obtained with CG-QMD-A are centered at 0°, i.e., in a planar conformation, thus correcting the inaccurate profiles delivered by the CG-Tr model. The CG-Tr distribution of ϕ_*ACAB*_ displays a lower barrier with respect to CG-QMD, which might reduce the rigidity of the chains, hence affecting the chain coiling, as discussed in the following. Overall, this analysis proves that despite averaging some degrees of freedom, key differences at the atomistic scale are successfully encoded into the CG model since the results show that the CG-QMD-A potentials accurately reproduce the distributions of dihedral DoFs, in close agreement with those obtained from the underlying atomistic model.

**Figure 9:**
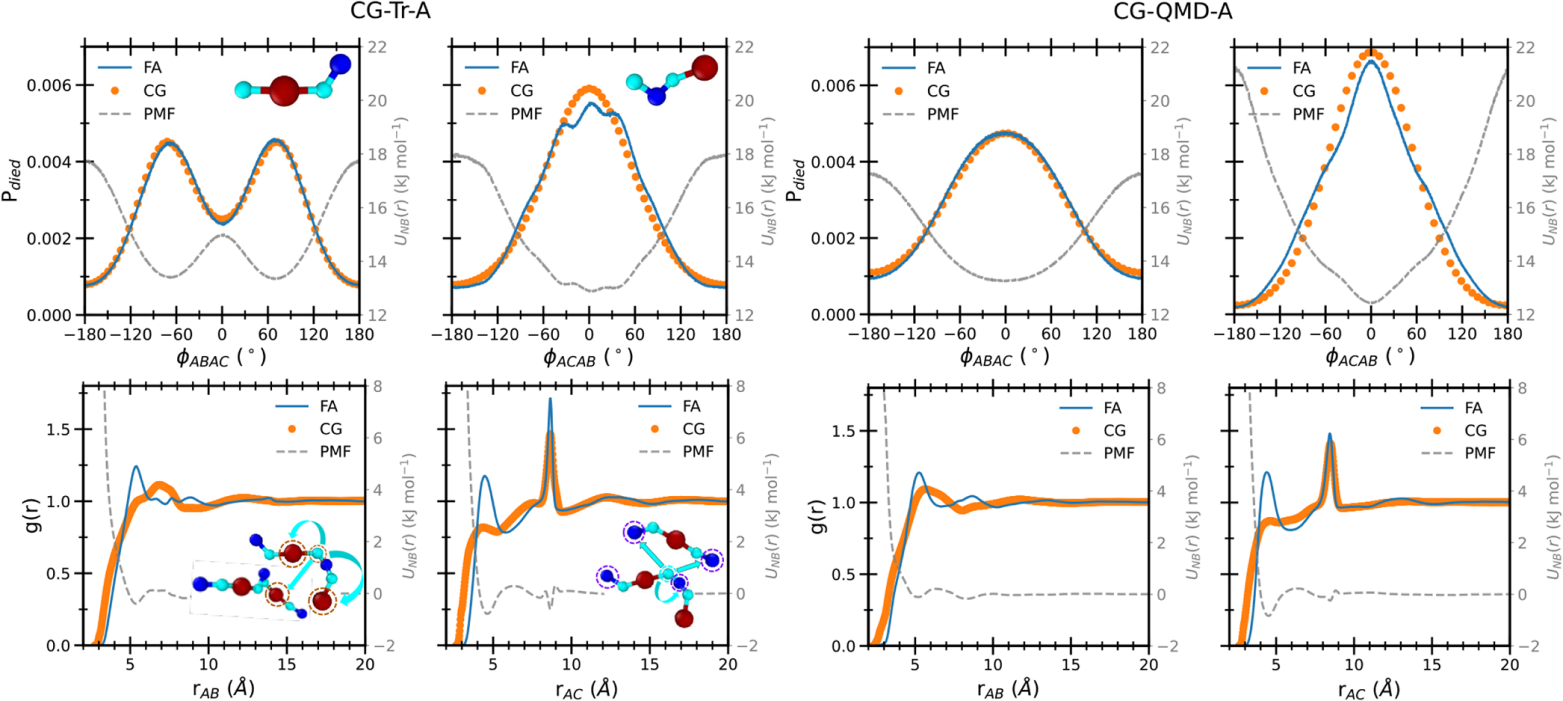
Dihedral probability (P*_died_*, top row) and radial distribution functions (g­(r), bottom row) computed for selected coordinates (see insets) of the 10**RMU** system at FA (blue lines) and CG levels (orange circles), using mapping scheme A. In all panels, the corresponding CG effective potentials are displayed with gray dashed lines. Parameterization results for both CG-Tr-A (left) and CG-QMD-FF (right) are shown for comparison.

The information concerning the interaction with the neighboring **RMUs** is instead passed to the CG model through the nonbonded distributions, g­(r), displayed for all models in the bottom rows of Figure 9. It appears that despite visible differences with the reference distributions, all the most significant features are well preserved. As in fact expected in a CG model, some details of the atomistic structure are inevitably lost: the sharp signals present at the atomistic level appear smoothed, particularly for the first peak of g_*AC*_(*r*) and g_*CC*_(*r*) and *g*(*r*)’s involving edges (see Section S6.2). To better understand the performance of the CG-FFs, it might hence be useful to recall that the total g­(r) arises from three different contributions: i) an intrachain term accounting for bead pairs lying less than three CG bonds apart, ii) nonbonded beads within the same chain, at larger distances, and iii) the bead pairs pertaining to different chains. By looking at Figure 9, it appears that both CG-FFs yield overall quite similar RDFs, all characterized by multiple peak profiles. The first peak (at *r* = 5–6 Å) corresponds to intramolecular interactions within the same **RMU**, while the next two, respectively, placed at *r* = 7–10 Å and *r* = 12–14 Å correspond mainly to interactions with the beads in the left and right RMUs within the same chain. Some of the profiles also have a small shoulder before the first peak, which corresponds to bonded 1–2 pairs. The effect of the interchain interactions on the recorded radial distributions is instead more subtle since the presence of neighboring chains may lead to a *plethora* of bead–bead arrangements, which may cover several distances, hence contributing to the overall broadening of the signal. Based on these findings, we can conclude that the CG parametrization successfully reproduces the local packing of each **RMU**, as described in the reference FAMD trajectories. In fact, both CG-QMD and CG-Tr CG-FFs inherit the atomistic structural features, proving the versatility of the adopted IBI method to capture changes in the **RMU** conformation predicted by different FA-FFs.

#### 3.3.2 CG Validation

The final validation of the whole protocol is achieved in the last module (VI), i.e., by analyzing the results of preliminary CGMD simulations, carried out with the parametrized CG-FFs on systems of *n* **RMU** chains (*n* = 10–100). Indeed, for a robust assessment of the CG model’s reliability, a careful comparison with the reference atomistic system should be carried out in terms of properties not considered in the CG-FF parametrization process described in the previous section. Therefore, to validate the transferability of the CG models developed above, we have extended the CG-FFs to a system consisting of 40 100**RMU** chains (16,000–24,000 beads), computing several global structural properties for comparison with those obtained from the fully atomistic model, as well as from those of the smaller systems. In this framework, the global structure of a polymer in its condensed phase can be described by static properties, such as the end-to-end distance (*R*_ee_) or radius of gyration (*R*_*g*_). While the former is analyzed in section S6.2.5 of the Supporting Information, Figure 10 shows, for all the six CG models, the comparison between the distributions of *R*_*g*_, extracted from CGMD runs or computed along the reference FAMD trajectories for the 100**RMU** system. In all cases, a very good match is found for *R*_*g*_ for both Tr- and QMD- derived CG-FFs, confirming the success of the models in reproducing the global structure of longer chains.

**Figure 10:**
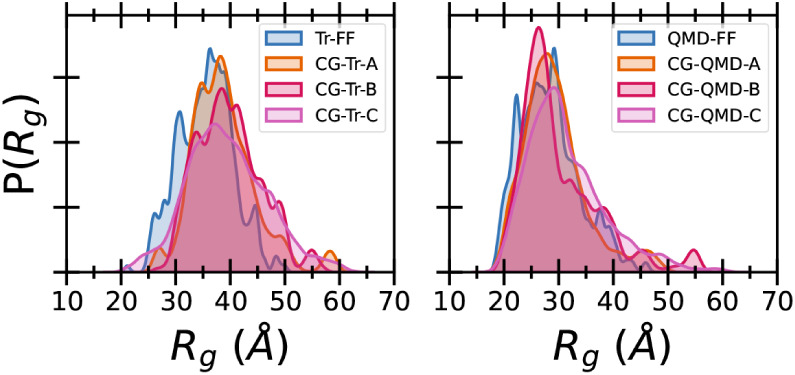
Comparison between FA and CG distributions of the radius of gyration, P­(*R_g_*), of the 100**RMU** system obtained by using the different mapping schemes. Parameterization results for both CG-Tr (left) and CG-QMD (right) are shown for comparison.

An additional macroscopic observable, which is key in characterizing the phase behavior of the polymer assembly, is the system bulk density. To this end, we compare for all investigated systems the densities computed with the different CG mappings with those delivered by the atomistic models. The data were averaged in the NPT ensemble along 50 ns runs, and the results are summarized in Table 5. As far as the adherence of the CG model to the parent FA description is concerned, the PET density predicted by both CG-Tr and CG-QMD is in close agreement with the one predicted by the corresponding atomistic model, the standard deviation being less than 1.5%. Consequently, the CG models based on Tr-FF trajectories suffer a similar underestimation of the experimental value registered with such FA-FF. This suggests that the CG models inherit the most relevant atomistic structural features, including any systematic error suffered by the FA-FF, which underlines the importance of carefully validating the model at every level in multiscale modeling. The differences among the A, B, and C mapping schemes are of the same order as the standard error, suggesting that, as far as the properties studied herein are concerned, the inclusion of additional CG beads to capture the effect of ring rigidity (scheme B) or flexibility of the ethylene glycol moiety (scheme C) has no noticeable impact on the density with respect to the more computationally efficient scheme A. These results confirm that all CG-QMD models can be confidently transferred to longer chains, preserving the accuracy of the original QMD-FF atomistic description yet allowing for a considerable computational benefit.

**Table 5:** Comparison of Density, in kg m^–3^, Computed for the 10**RMU** and 100**RMU** Systems along FA- and CGMD Runs, Carried Out in the NPT Ensemble under Standard Conditions Employing Different Models^a^

level	model	10**RMU**	100**RMU**
FA	Tr-FF	1256 ± 6.8	1251 ± 3.0
CG	Tr-A	1240 ± 19.2	1253 ± 5.7
	Tr-B	1259 ± 15.4	1253 ± 4.7
	Tr-C	1246 ± 17.8	1246 ± 5.4
FA	QMD-FF	1373 ± 6.7	1374 ± 2.2
CG	QMD-A	1372 ± 24.2	1363 ± 7.0
	QMD-B	1366 ± 17.0	1374 ± 5.2
	QMD-C	1377 ± 21.4	1376 ± 6.4
exp.^90,91^		1335–1345	

a In the last row, the experimental range is also reported for comparison.

## 4 Conclusions

We presented a computational protocol of general applicability that integrates information retrieved at different levels of theory in a modular workflow, eventually leading to accurate and consistent FA and CG force fields, suitable for an accurate modeling of polymer structure and dynamics. The whole procedure is rooted in first-principles training data and, as such, does not require the use of any experimental input, hence standing as a promising tool for the rational design of novel polymer materials. The proposed workflow is structured through different modules, where the resolution is gradually lowered yet maintaining the essential features of the parent reference description, hence allowing for building accurate FF models at the desired level of theory. The protocol is of wide applicability, rooted in the possibility to specifically tailor FA against the most appropriate reference, more detailed methodology, since QMD-FFs can be parametrized over any level of QM theory. The successive CG modeling, even if in the current implementation it follows a sequential strategy, can be applied to any FA distribution, and the IBI parametrization is targeted for refinement of specific interaction terms when needed. Noticeably, this would provide flexibility in practical applications, enabling selective updates of preexisting interaction potentials without requiring the entire CG model to be redefined from scratch.

For a first validation, we chose here the well-known PET polymer, exploiting the availability of a wide range of experimental and theoretical literature data for a thorough comparison. Besides controlling the quality of the achieved description along the whole parametrization process with a careful comparison of selected relevant properties measured on the target polymeric material, the information flow throughout consecutive modules is monitored by comparing their outcomes. First, the accurate description achieved at the QM level of PET’s potential energy surface and supramolecular interactions is successfully mapped onto the FA model through reliable QMD-FFs, parametrized with the Joyce/Picky procedure for small oligomers and successively extended to longer chains through the ChaMak tool. Lowering the level of resolution, the essential improvements achieved with the QMD description at the FA level with respect to a “blind” choice of a general-purpose transferable FF are successfully passed to the CG models, which proved to be capable of preserving the significant differences registered between their FA references. Noticeably, with respect to general-purpose models, both QMD-FF and CG-QMD delivered better agreement with the experiment in key properties such as density, glass transition temperature, and chain conformational features. Such an improvement can be traced back to the scarce adherence of general-purpose models based on transferability to the more accurate and reliable description achievable on smaller building blocks at a QM level. Finally, as far as PET is concerned, the achieved validation and the enhanced accuracy of the results delivered by both QMD-FF and CG-QMD models encourage their application in more extended FA and CGMD, which would allow for accessing additional key polymer properties, such as Young’s modulus or barrier and rheological properties. As a matter of fact, work in this direction is already in progress in our group.

From a more general perspective, the workflow proposed here brings in three major advancements: i) the integration in a consistent and unique workflow of robust and widely employed techniques such as DFT, QMD-FFs, FAMD, and CG modeling ensures reproducibility at all levels of theory; ii) it is completely general, thus in principle applicable to any polymer chain, as only the structure of its constitutional repeating unit is required as input; and iii) it is highly tunable and supports systematic refinement, making it well-suited for data-driven polymer discovery and *in silico* design. Despite the promising results achieved here for PET, the wide applicability of the protocol and its performance should be evaluated on more extended samples, targeting other polymers with varied structural properties designed for different applications. On the one hand, in the present implementation, our protocol is expected to transfer straightforwardly to other linear homopolymers, including 1**RMU**’s containing both aliphatic and aromatic moieties. Indeed, as indicated by the initial DFT benchmarking, the proposed reference QM level appears to be well calibrated and suited for building blocks also containing aromatic units and hence possibly subject to π-stacking interactions. Extension to copolymers featuring multiple repeating units arranged either in ordered architectures (e.g., block or alternating) or statistical sequences (e.g., random or gradient copolymers) is in principle feasible with slight modifications to the ChaMak procedure, for instance, considering larger building blocks as repeating units or randomized insertions. Additional developments are, on the other hand, required to tackle more complex yet interesting polymer architectures, such as those characterized by branched chains or “decorated” with functionalized additional groups. For these reasons, a near-future perspective will consist in further validating our protocol on other homopolymers, applying our workflow to poly­(ethylene furanoate) (PEF) and poly­(lactic acid) (PLA), two of the most promising biobased alternatives to PET, owing to their potentially reduced environmental impact over the full material life cycle. Assessing such systems will allow us to verify the capability of the workflow to support the development of more sustainable polymeric materials for packaging and, more generally, contribute to future strategies for plastic waste management. Nonetheless, prompted by the growing interest in advanced functional materials, such as those used in organic optoelectronics and photovoltaic devices,^94^ the ChaMak procedure could be extended to “decorated” and branched polymers, thus paving the route toward a more reliable and robust modeling in innovative advanced materials, where a highly accurate classical-level description is essential for integrations in novel mixed quantum-classical approaches, in turn fundamental for rationalizing the device functions and guiding innovative material design strategies.^95,96^

In this context, the integration with automated or data-driven schemes, e.g., based on machine-learning potentials, could further enhance both the applicability and computational efficiency of high-throughput virtual screening protocols.^97^ Reliable physical descriptors are indeed required by machine-learning procedures to make accurate predictions,^98^ and the potential predictivity and interpretability of the proposed protocol, both rooted in the physically grounded description retrieved from first-principles, make the latter a promising candidate for building such reference data. Moreover, the controlled multiscale consistency achieved across the different modules of the workflow addresses the current lack of consolidated computational strategies capable of coherently bridging multiple resolution levels.^10,98^ By preserving physical consistency from the quantum-mechanical scale up to coarser representations, the present procedures can provide hierarchically coherent data sets to complement data-driven approaches, ultimately improving their reliability and extending their predictive capability to genuinely novel classes of materials.

## Supplementary Material



## Notes

Additional computational details on DFT benchmark against CCSD­(T), QMD-FF models, and FAMD implementations; further information on CG-FF parametrization and simulations; supplementary validation results; a complete list of QMD-FF parameters for PET nRMU chains (PDF)

## References

[1] Sangroniz A., Zhu J.-B., Tang X., Etxeberria A., Chen E. Y., Sardon H. (2019). Packaging materials with desired
mechanical and barrier properties and full chemical recyclability. Nat. Commun..

[2] Sun H., Klok H.-A., Zhong Z. (2018). Polymers from
Nature and for Nature. Biomacromolecules.

[3] Wang W., Li P., Xie R., Ju X., Liu Z., Chu L. (2022). Designable
Micro-/Nano-Structured Smart Polymeric Materials. Adv. Mater..

[4] Makki H., Burke C., Nielsen C. B., Troisi A. (2025). Mapping the
structure-function
landscape of semiconducting polymers. Mater.
Horiz..

[5] Liu Y., Zhao J., Li Z., Mu C., Ma W., Hu H., Jiang K., Lin H., Ade H., Yan H. (2014). Aggregation
and morphology control enables multiple cases of high-efficiency polymer
solar cells. Nat. Commun..

[6] Wei Q., Ge Z., Voit B. (2019). Thermally Activated Delayed Fluorescent Polymers: Structures,
Properties, and Applications in OLED Devices. Macromol. Rapid Commun..

[7] Thompson R. C., Courtene-Jones W., Boucher J., Pahl S., Raubenheimer K., Koelmans A. A. (2024). Twenty years of microplastic pollution research—what
have we learned?. Science.

[8] Singh N., Walker T. R. (2024). Plastic recycling: A panacea or environmental pollution
problem. Npj Mater. Sustainability.

[9] Li Z., Tolba S. A., Wang Y., Alesadi A., Xia W. (2024). Modeling-driven
materials by design for conjugated polymers: Insights into optoelectronic,
conformational, and thermomechanical properties. Chem. Commun..

[10] Burke C., Troisi A. (2025). Models connecting microstructure and charge transport
in disordered semiconducting polymers: From theories to digital design. Mater. Horiz..

[11] Turney H. N., Matta M. (2025). Atomistic Polymer Modeling: Recent Advances and Challenges in Building
and Parametrization Workflows. Macromolecules.

[12] Cerezo J., Gierschner J., Santoro F., Prampolini G. (2024). Explicit Modelling
of Spectral Bandshapes by a Mixed Quantum-Classical Approach: Solvent
Order and Temperature Effects in the Optical Spectra of Distyrylbenzene. ChemPhyschem.

[13] Fang C.-E., Tsai Y.-C., Scheurer C., Chiu C.-C. (2021). Revised Atomic Charges
for OPLS Force Field Model of Poly­(Ethylene Oxide): Benchmarks and
Applications in Polymer Electrolyte. Polymers.

[14] Davel C. M., Bernat T., Wagner J. R., Shirts M. R. (2024). Parameterization
of General Organic Polymers within the Open Force Field Framework. J. Chem. Inf. Model.

[15] Singh V., Patra S., Murugan N. A., Toncu D.-C., Tiwari A. (2022). Recent trends
in computational tools and data-driven modeling for advanced materials. Mater. Adv..

[16] Müller-Plathe F. (2002). Coarse-Graining
in Polymer Simulation: From the Atomistic to the Mesoscopic Scale
and Back. ChemPhyschem.

[17] Harmandaris V. A., Adhikari N. P., van der
Vegt N. F. A., Kremer K. (2006). Hierarchical Modeling
of Polystyrene: From Atomistic to Coarse-Grained Simulations. Macromolecules.

[18] Kempfer K., Devémy J., Dequidt A., Couty M., Malfreyt P. (2019). Development
of Coarse-Grained Models for Polymers by Trajectory Matching. ACS Omega.

[19] Jin J., Pak A. J., Durumeric A. E. P., Loose T. D., Voth G. A. (2022). Bottom-up
Coarse-Graining: Principles and Perspectives. J. Chem. Theory And Comput..

[20] Reisjalali M., Manurung R., Carbone P., Troisi A. (2022). Development
of hybrid
coarse-grained atomistic models for rapid assessment of local structuring
of polymeric semiconductors. Mol. Syst. Des.
Eng..

[21] Yellam, K. ; Katiyar, R. S. ; Jha, P. K. Current Perspective on Atomistic Force Fields of Polymers. Forcefields for Atomistic-Scale Simulations: Materials and Applications; Springer Nature: Singapore, 2022, pp. 51–79

[22] Gartner T.
E. I., Jayaraman A. (2019). Modeling and
Simulations of Polymers: A Roadmap. Macromolecules.

[23] Schmid F. (2023). Understanding
and Modeling Polymers: The Challenge of Multiple Scales. ACS Polym. Au.

[24] Smith L., Karimi-Varzaneh H. A., Finger S., Giunta G., Troisi A., Carbone P. (2024). Framework for a High-Throughput Screening
Method to
Assess Polymer/Plasticizer Miscibility: The Case of Hydrocarbons in
Polyolefins. Macromolecules.

[25] Behbahani A. F., Schneider L., Rissanou A., Chazirakis A., Bačová P., Jana P. K., Li W., Doxastakis M., Polińska P., Burkhart C., Müller M., Harmandaris V. A. (2021). Dynamics and Rheology of Polymer Melts via Hierarchical
Atomistic, Coarse-Grained, and Slip-Spring Simulations. Macromolecules.

[26] Bellussi F. M., Roscioni O. M., Ricci M., Fasano M. (2021). Anisotropic
Electrostatic
Interactions in Coarse-Grained Water Models to Enhance the Accuracy
and Speed-Up Factor of Mesoscopic Simulations. J. Phys. Chem. B.

[27] Nkepsu
Mbitou R. L., Goujon F., Dequidt A., Latour B., Devémy J., Blaak R., Martzel N., Emeriau-Viard C., Tchoufag J., Garruchet S., Munch E., Hauret P., Malfreyt P. (2022). Consistent and Transferable Force Fields for Statistical
Copolymer Systems at the Mesoscale. J. Chem.
Theory Comput..

[28] Riniker S. (2018). Fixed-Charge
Atomistic Force Fields for Molecular Dynamics Simulations in the Condensed
Phase: An Overview. J. Chem. Inf. Mod..

[29] Jorgensen W. L., Tirado-Rives J. (2005). Potential
Energy Functions for Atomic-Level Simulations
of Water and Organic and Biomolecular Systems. Proc. Natl. Acad. Sci. U. S. A..

[30] Maier J. A., Martinez C., Kasavajhala K., Wickstrom L., Hauser K. E., Simmerling C. (2015). ff14SB: Improving
the Accuracy of
Protein Side Chain and Backbone Parameters from ff99SB. J. Chem. Theory Comput..

[31] Rukmani S. J., Kupgan G., Anstine D. M., Colina C. M. C. (2019). A molecular
dynamics
study of water-soluble polymers: Analysis of force fields from atomistic
simulations. Mol. Sim..

[32] Du
Bay K. H., Hall M. L., Hughes T. F., Wu C., Reichman D. R., Friesner R. A. (2012). Accurate Force Field Development
for Modeling Conjugated Polymers. J. Chem. Theory
Comput..

[33] Mohanty S., Stevenson J., Browning A. R., Jacobson L., Leswing K., Halls M. D., Afzal M. A. F. (2023). Development of scalable and generalizable
machine learned force field for polymers. Sci.
Rep..

[34] Semmeq A., Del Galdo S., Chiarini M., Daidone I., Casieri C. (2024). Structural
and dynamic behaviour of concentrated aqueous solutions of (poly)­ethylene
glycols: Insight into the impact of hydrophobicity, hydrogen bonding
and chain length. J. Mol. Liq..

[35] Giannini S., Martinez P. M., Semmeq A., Galvez J. P., Piras A., Landi A., Padula D., Vilhena J. G., Cerezo J., Prampolini G. (2025). JOYCE3.0:
A General Protocol for the Specific Parametrization
of Accurate Intramolecular Quantum Mechanically Derived Force Fields. J. Chem. Theory Comput..

[36] Lu C., Wu C., Ghoreishi D., Chen W., Wang L., Damm W., Ross G. A., Dahlgren M. K., Russell E., Von Bargen C. D., Abel R., Friesner R. A., Harder E. D. (2021). OPLS4:
Improving
Force Field Accuracy on Challenging Regimes of Chemical Space. J. Chem. Theory Comput..

[37] Semmeq A., Del Galdo S., Chiarini M., Daidone I., Casieri C. (2024). Macromolecular
vs molecular crowding in aqueous solutions: A comparative study of
PEG400 and ethylene glycol. J. Mol. Liq..

[38] Burke C., Makki H., Troisi A. (2024). From Chemical
Drawing to Electronic
Properties of Semiconducting Polymers in Bulk: A Tool for Chemical
Discovery. J. Chem. Theory Comput..

[39] Landi A., Reisjalali M., Elliott J. D., Matta M., Carbone P., Troisi A. (2023). Simulation
of polymeric mixed ionic and electronic
conductors with a combined classical and quantum mechanical model. J. Mater. Chem. C.

[40] Sutton C., Körzdörfer T., Gray M. T., Brunsfeld M., Parrish R. M., Sherrill C. D., Sears J. S., Brédas J.-L. (2014). Accurate
description of torsion potentials in conjugated polymers using density
functionals with reduced self-interaction error. J. Chem. Phys..

[41] Kania A., Sarapata K., Gucwa M., Wójcik-Augustyn A. (2021). Optimal Solution
to the Torsional Coefficient Fitting Problem in Force Field Parametrization. J. Phys. Chem. A.

[42] Wildman J., Repiščák P., Paterson M. J., Galbraith I. (2016). General Force-Field
Parametrization Scheme for Molecular Dynamics Simulations of Conjugated
Materials in Solution. J. Chem. Theory Comput..

[43] König G., Riniker S. (2020). On the faithfulness
of molecular mechanics representations
of proteins towards quantum-mechanical energy surfaces. Interface Focus.

[44] Csizi K.-S., Reiher M. (2023). Universal QM/MM approaches
for general nanoscale applications. WIRES Comp.
Mol. Sci..

[45] Odinokov A., Yakubovich A., Son W.-J., Jung Y., Choi H. (2021). Exploiting
the quantum mechanically derived force field for functional materials
simulations. Npj Comput. Mater..

[46] Prampolini G., Silveira L. G. D., Vilhena J. G., Livotto P. R. (2022). Predicting
Spontaneous
Orientational Self-Assembly: In Silico Design of Materials with Quantum
Mechanically Derived Force Fields. J. Phys.
Chem. Lett..

[47] Pawlak R., Vilhena J. G., D’Astolfo P., Liu X., Prampolini G., Meier T., Glatzel T., Lemkul J. A., Häner R., Decurtins S., Baratoff A., Pérez R., Liu S.-X., Meyer E. (2020). Sequential Bending and Twisting around
C–C Single Bonds by Mechanical Lifting of a Pre-Adsorbed Polymer. Nano Lett..

[48] D’Astolfo P., Vilhena J. G., Rothenbühler S., Drechsel C., Gutiérrez-Varela O., Häner R., Decurtins S., Liu S.-X., Prampolini G., Pawlak R., Meyer E. (2025). On-Surface Synthesis and Cryogenic
Exfoliation of Sterically Frustrated Atropisomers. ACS Nano.

[49] Yelishala S. C., Zhu Y., Martinez P. M., Chen H., Habibi M., Prampolini G., Cuevas J. C., Zhang W., Vilhena J. G., Cui L. (2025). Phonon interference
in single-molecule junctions. Nat. Mater..

[50] Vilhena J. G., Greff da Silveira L., Livotto P. R., Cacelli I., Prampolini G. (2021). Automated
Parameterization of Quantum Mechanically Derived Force Fields for
Soft Materials and Complex Fluids: Development and Validation. J. Chem. Theory Comput..

[51] Cacelli I., Prampolini G. (2007). Parametrization and Validation of
Intramolecular Force
Fields Derived from DFT Calculations. J. Chem.
Theory Comput..

[52] Cacelli I., Cimoli A., Livotto P. R., Prampolini G. (2012). An Automated
Approach for the Parameterization of Accurate Intermolecular Force-Fields:
Pyridine as a Case Study. J. Comput. Chem..

[53] Sangkhawasi M., Remsungnen T., Vangnai A. S., Poo-Arporn R. P., Rungrotmongkol T. (2022). All-Atom Molecular
Dynamics Simulations on a Single
Chain of PET and PEV Polymers. Polymers.

[54] Lazarenko D., Schmidt G. P., Crowley M. F., Beckham G. T., Knott B. C. (2025). Molecular
Details of Polyester Decrystallization via Molecular Simulation. Macromolecules.

[55] Polêto M. D., Lemkul J. A. (2025). Structural and Electronic Properties
of Poly­(ethylene
terephthalate) (PET) from Polarizable Molecular Dynamics Simulations. Macromolecules.

[56] Guillard V., Gaucel S., Fornaciari C., Angellier-Coussy H., Buche P., Gontard N. (2018). The Next Generation
of Sustainable
Food Packaging to Preserve Our Environment in a Circular Economy Context. Front. Nutr..

[57] Bauer A.-S., Tacker M., Uysal-Unalan I., Cruz R. M. S., Varzakas T., Krauter V. (2021). Recyclability and Redesign
Challenges in Multilayer
Flexible Food Packaging—A Review. Foods.

[58] Zhao S., Kvale K. F., Zhu L., Zettler E. R., Egger M., Mincer T. J., Amaral-Zettler L. A., Lebreton L., Niemann H., Nakajima R., Thiel M., Bos R. P., Galgani L., Stubbins A. (2025). The distribution of
subsurface microplastics in the
ocean. Nature.

[59] Frisch, M. J. ; Trucks, G. W. ; Schlegel, H. B. ; Scuseria, G. E. ; Robb, M. A. ; Cheeseman, J. R. ; Scalmani, G. ; Barone, V. ; Petersson, G. A. ; Nakatsuji, H. , Gaussian16 Revision D.02; Gaussian Inc.: Wallingford CT, 2016.

[60] Prampolini, G. ; Cerezo, J. ; Giannini, S. ; De Mitri, N. ; Cacelli, I. ; Joyce3.0, intra-molecular force field parameterization software, 2024. http://www.iccom.cnr.it/en/joyce-2/;.

[61] Semmeq, A. ; Prampolini, G. ; Chamak: A topology buiolkder for polymers, 2025. https://github.com/ASEMMEQ/ChaMak;.

[62] Abraham M. J., Murtola T., Schulz R., Páll S., Smith J. C., Hess B., Lindahl E. (2015). GROMACS: High
performance
molecular simulations through multi-level parallelism from laptops
to supercomputers. SoftwareX.

[63] Prampolini, G. A. C. ; Cacelli, I. ; Picky3.0, a Fortran 77 code for inter-molecular force field parameterization; 2020. http://www.iccom.cnr.it/en/picky-en/.

[64] Mirzoev A., Lyubartsev A. P. (2013). MagiC: Software Package for Multiscale Modeling. J. Chem. Theory Comput..

[65] Thompson A. P., Aktulga H. M., Berger R., Bolintineanu D. S., Brown W. M., Crozier P. S., in’t Veld P. J., Kohlmeyer A., Moore S. G., Nguyen T. D., Shan R., Stevens M. J., Tranchida J., Trott C., Plimpton S. J. (2022). LAMMPS
- a flexible simulation tool for particle-based materials modeling
at the atomic, meso, and continuum scales. Comput.
Phys. Commun..

[66] Rezáč J., Hobza P. (2013). Describing Noncovalent Interactions beyond the Common Approximations:
How Accurate Is the “Gold Standard,” CCSD­(T) at the
Complete Basis Set Limit?. J. Chem. Theory Comput..

[67] Rezáč J., Hobza P. (2016). Benchmark Calculations
of Interaction Energies in Noncovalent Complexes
and Their Applications. Chem. Rev..

[68] Prampolini G., Livotto P. R., Cacelli I. (2015). Accuracy of
Quantum Mechanically
Derived Force-Fields Parameterized from Dispersion-Corrected DFT Data:
The Benzene Dimer as a Prototype for Aromatic Interactions. J. Chem. Theory Comput..

[69] Grimme S., Ehrlic S., Goerigk L. (2011). Effect of the Damping
Function in
Dispersion Corrected Density Functional Theory. J. Comput. Chem..

[70] Martinez, P. M. ; Piras, A. ; Cerezo, J. ; Galvez, J. P. ; Giannini, S. ; Landi, A. ; Padula, D. ; Semmeq, A. ; Vilhena, J. G. ; Prampolini, G. ; Joyce; https://joyce-documentation.gitlab.io/;. 2024. 10.1021/acs.jctc.5c00010 40066838

[71] Amovilli C., Cacelli I., Campanile S., Prampolini G. (2002). Calculation
of the Intermolecular Energy of Large Molecules by a Fragmentation
Scheme: Application to the 4-*n*-Pentyl-4’-
Cyanobiphenyl (5CB) Dimer. J. Chem. Phys..

[72] Lightfoot J., Buchard A., Castro-Dominguez B., Parker S. (2022). Comparative Study of
Oxygen Diffusion in Polyethylene Terephthalate and Polyethylene Furanoate
ff Using Molecular Modeling: Computational Insights into the Mechanism
for Gas Transport in Bulk Polymer Systems. Macromolecules.

[73] Dodda L. S., Cabeza de Vaca I., Tirado-Rives J., Jorgensen W. L. (2017). LigParGen
web server: An automatic OPLS-AA parameter generator for organic ligands. Nucleic Acids Res..

[74] Hess B., Bekker B., Berendsen H., Fraaije J. (1997). LINCS: A linear constraint
solver for molecular simulations. J. Comput.
Chem..

[75] Essmann U., Perera L., Berkowitz M. L., Darden T., Lee H., Pedersen L. G. (1995). A smooth particle
mesh Ewald method. J. Chem. Phys..

[76] Bussi G., Donadio D., Parrinello M. (2007). Canonical
sampling through velocity
rescaling. J. Chem. Phys..

[77] Bernetti M., Bussi G. (2020). Pressure control using
stochastic cell rescaling. J. Chem. Phys..

[78] Giulini M., Menichetti R., Shell M. S., Potestio R. (2020). An Information-Theory-Based
Approach for Optimal Model Reduction of Biomolecules. J. Chem. Theory Comput..

[79] Chakraborty M., Xu J., White A. D. (2020). Is preservation
of symmetry necessary for coarse-graining?. Phys. Chem. Chem. Phys..

[80] Choudhury R. P., Lee J. S., Kriegel R. M., Koros W. J., Beckham H. W. (2012). Chain Dynamics
in Antiplasticized and Annealed Poly­(ethylene terephthalate) Determined
by Solid-State NMR and Correlated with Enhanced Barrier Properties. Macromolecules.

[81] Reith D., Meyer H., Müller-Plathe F. (2001). Mapping Atomistic to
Coarse-Grained Polymer Models Using Automatic Simplex Optimization
To Fit Structural Properties. Macromolecules.

[82] Reith D., Pütz M., Müller-Plathe F. (2003). Deriving effective
mesoscale potentials from atomistic simulations. J. Comput. Chem..

[83] Mirzoev A., Nordenskiöld L., Lyubartsev A. (2019). Magic v.3: An integrated software
package for systematic structure-based coarse-graining. Comput. Phys. Commun..

[84] Golmohammadi N., Boland-Hemmat M., Barahmand S., Eslami H. (2020). Coarse-grained molecular
dynamics simulations of poly­(ethylene terephthalate). J. Chem. Phys..

[85] Tuckerman M., Berne B. J., Martyna G. J. (1992). Reversible multiple time scale molecular
dynamics. J. Chem. Phys..

[86] Isele-Holder R. E., Mitchell W., Ismail A. E. (2012). Development
and application of a
particle-particle particle-mesh Ewald method for dispersion interactions. J. Chem. Phys..

[87] Cerezo J., Prampolini G., Cacelli I. (2018). Developing accurate intramolecular
force fields for conjugated systems through explicit coupling terms. Theor. Chem. Acc..

[88] Schmidt-Rohr K., Hu W., Zumbulyadis N. (1998). Elucidation
of the Chain Conformation in a Glassy Polyester,
PET, by Two-Dimensional NMR. Science.

[89] Joo S., Cho I. J., Seo H., Son H. F., Sagong H.-Y., Shin T. J., Choi S. Y., Lee S. Y., Kim K.-J. (2018). Structural
insight into molecular mechanism of poly­(ethylene terephthalate) degradation. Nat. Commun..

[90] Thompson A. B., Woods D. W. (1955). Density of Amorphous
Polyethylene Terephthalate. Nature.

[91] Zekriardehani S., Joshi A. S., Jabarin S. A., Gidley D. W., Coleman M. R. (2018). Effect
of Dimethyl Terephthalate and Dimethyl Isophthalate on the Free Volume
and Barrier Properties of Poly­(ethylene terephthalate) (PET): Amorphous
PET. Macromolecules.

[92] Zhang Y., Zhang J., Lu Y., Duan Y., Yan S., Shen D. (2004). Glass Transition Temperature
Determination of Poly­(ethylene terephthalate)
Thin Films Using Reflection-Absorption FT-IR. Macromolecules.

[93] Qian R., Shen D., Sun F., Wu L. (1996). The effects of physical
ageing on conformational changes of poly­(ethylene terephthalate) in
the glass transition region. Macromol. Chem.
Phys..

[94] Zhang G., Lin F., Qi F., Heumüller T., Distler A., Egelhaaf H., Li N., Chow P., Brabec C. J., Jen A., Yip H. (2022). Renewed Prospects
for Organic Photovoltaics. Chem. Rev..

[95] Giannini S., Cerdá J., Prampolini G., Santoro F., Beljonne D. (2024). Dissecting
the nature and dynamics of electronic excitations in a solid-state
aggregate of a representative non-fullerene acceptor. J. Mater. Chem. C.

[96] Giannini S., Segalina A., Padula D., Cantina M., Pastore M., Prampolini G., Santoro F. (2026). Delocalization versus Coherence under
Vibrational and Environmental Disorder in Photoexcited Supramolecular
Aggregates. J. Am. Chem. Soc..

[97] Omar Ö. H., Del Cueto M., Nematiaram T., Troisi A. (2021). High-throughput virtual
screening for organic electronics: A comparative study of alternative
strategies. J. Mater. Chem. C.

[98] Zhao Z.-W., Del Cueto M., Troisi A. (2022). Limitations of machine learning models
when predicting compounds with completely new chemistries: Possible
improvements applied to the discovery of new non-fullerene acceptors. Digital Discovery.

