# Supplementary material for: From Quantum Mechanics to Coarse-Grained Models: Bridging the Gap toward Polymer Rational Design
Source: J Chem Theory Comput. 2026 Apr 1;22(10):5210–26. doi: 10.1021/acs.jctc.5c02092 (PMC13217569; doi:10.1021/acs.jctc.5c02092)
Supplement: Supplementary file 1 [file ct5c02092_si_001.pdf]

SUPPORTING INFORMATION

From Quantum Mechanics to Coarse Grained Models: Bridging the Gap toward Polymer Rational Design

Abderrahmane Semmeq,[a,†] Andoni Ugartemendia, [b,c,†], Alessandro Mossa,[b] Serena Coiai,[a] Giorgia Brancolini,[b,*] Giacomo Prampolini[a,*]

[a]*Istituto di Chimica dei Composti OrganoMetallici,* Consiglio Nazionale delle Ricerche (ICCOM-CNR), Area della Ricerca, via G. Moruzzi 1, I-56124 Pisa, Italy

[b]*Center S3, Istituto di Nanoscienze,* Consiglio Nazionale delle Ricerche (Nano-CNR), via Campi 213/A, 41125 Modena, Italy

[c]*Polimero eta Material Aurreratuak: Fisika, Kimika eta Teknologia Saila*, Kimika Fakultatea, Euskal Herriko Unibertsitatea (UPV/EHU) and Donostia International Physics Center (DIPC), M. de Lardizabal Pasealekua 3, Donostia, Euskadi, Spain

[*]email: giorgia.brancolini@nano.cnr.it, giacomo.prampolini@pi.iccom.cnr.it

[†] contributed equally to this work

Contents

S1 DFT benchmark against CCSD(T) **S4**

S1.1 CCSD(T)/CBS S4

S1.2 DFT benchmarks S6

S2 QMD-FF Parameterization **S8**

S2.1 Intramolecular Term S8

S2.1.1 Small oligomers S8

S2.1.2 Polymer chains S10

S2.2 Intermolecular Term S12

S3 QMD-FF parameters and additional validation **S13**

S3.1 Intramolecular S13

S3.1.1 1**RMU** Dihedral scans S13

S3.1.2 1**RMU** QMD-FF parameters S13

S3.1.3 2**RMU** QMD-FF parameters S15

S3.1.4 3**RMU** QMD-FF parameters S20

S3.2 Intermolecular S23

S3.2.1 PICKY parameterization S23

S3.2.2 *n***RMU** QMD-FF parameters S26

S4 FAMD simulations **S29**

S4.1 Equilibration procedure S29

S4.2 T_g calculation S29

S4.3 Additional FAMD results S30

S5 CG-FF parameterization **S33**

S5.1 Iterative Boltzmann inversion S33

S5.2 Postprocessing for CGMD simulations S36

S6 CGMD simulations **S37**

S6.1 Convergence analysis of CG models S37

S6.2 Structural properties comparison of CG models S39

S6.2.1 Bond distributions S39

S6.2.2 Angle distributions S45

S6.2.3 Dihedral distributions S51

S6.2.4 Radial distribution functions S57

S6.2.5 Global structural properties S69

S1 DFT benchmark against CCSD(T)

S1.1 CCSD(T)/CBS

The QM interaction energy ΔE^{QM} between two single repeating monomer units (RMUs), $R1$ and $R2$, is computed as

$$\Delta E^{QM} = E_{R1\cdots R2} - E_{R1} - E_{R2} \tag{S1}$$

where the E_k is the total energy of either the non-covalent dimer $R1\cdots R2$ or the isolated single units ($R1$ or $R2$), computed at the selected level of theory. To benchmark DFT accuracy in describing the non-covalent interaction between two single PET's RMUs, a highly accurate reference database was specifically built by computing ΔE^{QM} at CCSD(T)/CBS level for several geometrical chain pair arrangements, built by displacing one 1**RMU** with respect to the other as shown in Figure S1, along a displacement vector **R**, or through a rotation around a definite axis (β). Two specific arrangements of non covalent stacked dimers (anti-parallel face-to-face,

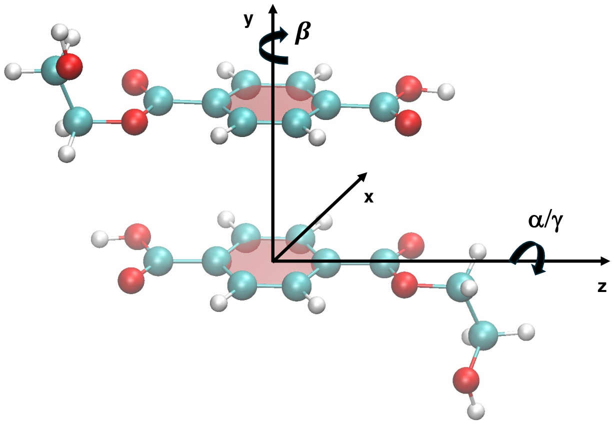

Figure S1: Definition of chain pair descriptors (x,y,z,α,β and γ).

AFF, and rotated face-to-face, RFF) are considered to benchmarks a series of different DFT functionals, to ascertain the possible differences arising among them.

The CCSD(T) interaction energies at the basis set limit [1] are recovered from the MP2/CBS limit (ΔU_{CBS}^{MP2}), and applying a correction, $\delta_{CCSD(T)}$, which can be estimated from the difference between the MP2 and CCSD(T) values, computed at the same geometry with a smaller basis

set B, i.e.

$$\Delta E_{CBS}^{CCSD(T)} = \Delta E_{CBS}^{MP2} + \delta_{CCSD(T)} \tag{S2}$$

where

$$\delta_{CCSD(T)} = \Delta E_B^{CCSD(T)} - \Delta E_B^{MP2} \tag{S3}$$

Following previous works, [2–4] the MP2 interaction energy at CBS was estimated by the Halkier extrapolation scheme [5] employing the cc-pVDZ and cc-pVTZ basis sets, whereas the $\Delta CCSD(T)$ correction was computed exploiting the former basis set. Indeed, it was found [3,4] that this choice does not introduce appreciable differences (<0.05 kJ/mol for benzene) with respect to more refined yet computationally expensive schemes. [6] In both CCSD(T) and MP2 calculations, all carried out with the GAUSSIAN16 [7] software, the basis set superposition error (BSSE) was taken into account by the standard Counterpoise (CP) correction. [8] In Table S1, the CCSD(T)/CBS computed energies are reported for all considered dimer arrangements, together with all data required for the estimation of the CBS.

Geom	R (Å)	ΔE^{MP2} (kJ/mol)			$\Delta E^{CCSD(T)}$ (kJ/mol)	
	β (degr)	cc-pvDz	cc-pvTz	CBS	cc-pvDz	CBS
	3.25	-7.11	-25.48	-38.87	13.85	-17.91
AFF	3.50	-20.29	-33.64	-43.30	-5.98	-29.04
	5.00	-7.53	-10.13	-11.92	-5.10	-9.46
	0.50	-25.37	-39.25	-49.14	-12.68	-36.45
APD	1.25	-30.33	-44.62	-54.73	-18.66	-43.06
	3.00	-25.69	-38.55	-47.48	-16.36	-38.15
	4.50	-3.38	-13.23	-20.41	4.85	-12.17
TS	4.75	-12.53	-19.08	-24.01	-6.86	-18.35
	5.25	-12.30	-15.52	-18.00	-9.37	-15.08
	45.00	-21.63	-34.52	-43.68	-8.95	-31.00
RFF	90.00	-19.00	-30.27	-38.24	-6.07	-25.31
	165.00	-22.26	-35.71	-45.34	-9.33	-32.42

Table S1: CCSD(T) interaction energies and thier contributions ΔU^k (kJ/mol), computed at different geometrical arrangements for two interacting 1**RMU**'s (see the insets in Figure S2).

S1.2 DFT benchmarks

A wide range of DFT functionals is explored, benchmarking their accuracy against the reference CCSD(T)/CBS data discussed in the previous section. Concretely, for all the four arrangements considered for the reference calculations, an interaction energy curve is obtained for each tested functional, as displayed in Figure S2.

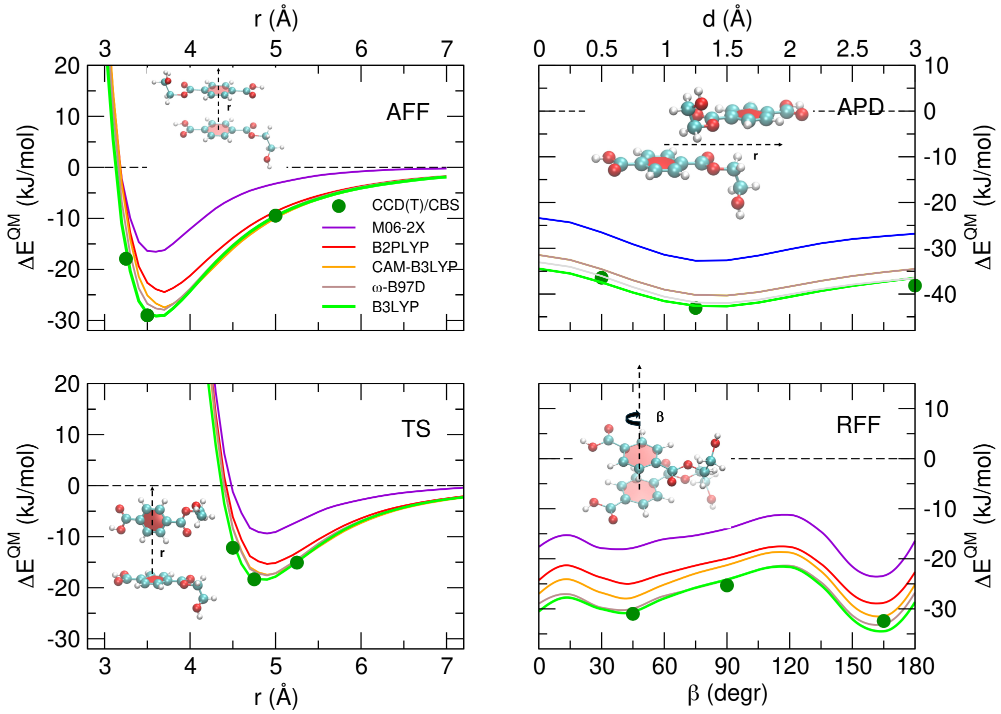

Figure S2: Benchmark of different DFT functional against CCSD(T)/CBS reference, computed for the four arrangements of a 1**RMU** pair shown on the insets. All DFT calculations were carried out with the 6-311G(d,p) basis set.

In all cases, the best agreement with the reference values was obtained with the B3LYP functional, taking into account the dispersion correction through the D3BJ scheme. [9] Figure S3 confirms the excellent agreement found between B3LYP/D3BJ and the reference data in terms of non-covalent interaction energies for all the considered arrangements (see also the insets of Figure S2). Indeed, the average unsigned error registered using B3LYP-D3BJ on the sampled configurations with respect to CCSD(T)/CBS is around 1 kJ/mol, with a gain in computational cost of almost three orders of magnitude (around 4 hours against $\sim$ 4000 hours

for each geometry on a single Xeon(R)@2.20GHz processor). Considering that the number

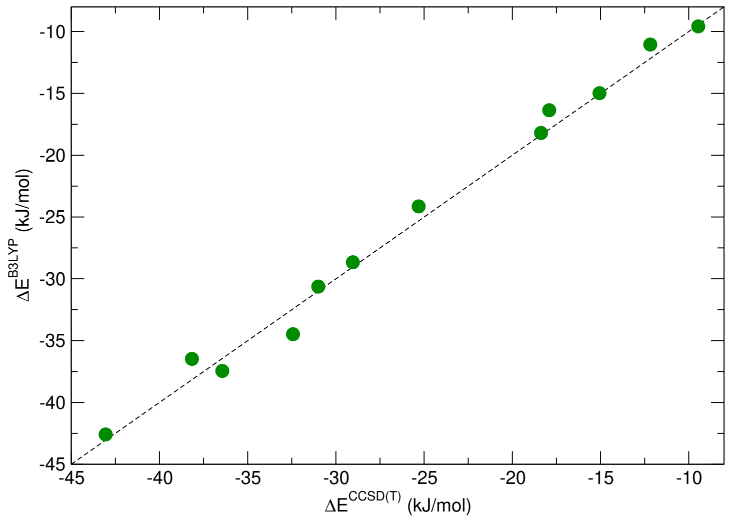

Figure S3: Correlation plot of the interaction energies ΔE computed for a pair of 1**RMU** in the considered arrangements either ar CCSD(T)/CBS or at B3LYP-D3BJ/6-311G(d,p) level.

of 4**RMU** pairs expected to be sampled during the QMD-FF parameterization of the intermolecular term (*vide infra*) is more 300, and that the loss in accuracy with respect the higher level description is seemingly negligible, we deemed B3LYP-D3BJ a reliable enough reference for the subsequent steps of the workflow.

S2 QMD-FF Parameterization

The two potential energy terms entering the total QMD-FF partition given in the main text can be be further being defined as

$$E_{intra}^{QMD-FF} = \sum_{\alpha=1}^{N_c} u_{\alpha}^{intra} \; ; \; E_{inter}^{QMD-FF} = \sum_{i=1}^{N_{at}} \sum_{j=1}^{N_{at}} u_{ij}^{inter} \tag{S4}$$

where α runs over the polymer chains composing the system and i, j are dummy indexes indicating the sites of two different interacting chains of N_{at} atoms. Standard expressions for both u^{intra} and u^{inter} were adopted both to preserve compatibility with most popular MD engines and in view of the computational effort required by the extensive FAMD runs.

S2.1 Intramolecular Term

The QMD-FF intra-molecular term, u^{intra}, is modeled by the standard class I expression

$$\begin{aligned} u^{intra} = \frac{1}{2}\sum_{s}^{N_s} k_s (d - d^0)^2 + \frac{1}{2}\sum_{b}^{N_b} k_b (\theta - \theta^0)^2 + \frac{1}{2}\sum_{st}^{N_{st}} k_{st} (\phi - \phi^0)^2 + \\ \sum_{\mu}^{N_{ft}} \sum_{j}^{N_{cos\mu}} k_{j\mu}^{ft} \left[1 + cos(n_j^{\mu} \delta_{\mu} - \gamma_j^{\mu})\right] + \\ \sum_{i=1}^{N_{pairs}} \sum_{j=1}^{N_{pairs}} \left(4\varepsilon_{ij}^{intra} \left[\left(\frac{\sigma_{ij}^{intra}}{r_{ij}} \right)^{12} - \left(\frac{\sigma_{ij}^{intra}}{r_{ij}} \right)^{6} \right] \right) + \left(\frac{[q_i q_j]}{r_{ij}} \right) \end{aligned} \tag{S5}$$

where the internal energy of the system is expressed as a function of polymer chain's internal coordinates (ICs), i.e. bond length (d), angles (θ), dihedrals (ϕ, δ) and non-bonded distances (r_{ij}) defined between atom pairs within each chain.

S2.1.1 Small oligomers

Based on the Hessian matrices specifically computed for each target oligomer and stored in the QM database, the IC set can be further partitioned into "stiff" and "soft" modes [10, 11] as discussed in the main text and detailed in Figure S4. Stiff ICs (i.e. bond lengths, bending angles and "improper" dihedrals) are parameterized at once through the harmonic terms of equation

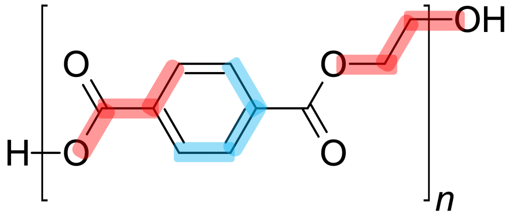

Figure S4: Molecular structure of the PET repeating monomeric unit (1**RMU**). Blue highlight shows an example of "stiff" internal coordinates, red highlight shows an example of "soft" internal coordinates

(S5), by minimizing the reduced JOYCE objective function [11]:

$$I^H_{g_0} = \sum_K^{3N-6} \sum_{K \leq L}^{3N-6} \frac{2W''_{gKL}}{C''} \left[H_{KL} - \left(\frac{\partial^2 u^{intra}}{\partial Q_K \partial Q_L} \right) \right]^2_{g_0} \tag{S6}$$

where the double sum runs over QM normal modes K and L, C'' is a normalization factor and W''_{gKL} a normalized weight, while $\mathbf{H}$ is the QM Hessian matrix. Despite the stiff coordinates parameterization only requires the QM equilibrium geometry and its Hessian matrix, an accurate modeling of the rotations around σ-bonds of the dihedrals highlighted in red in Figure S4 requires reference QM relaxed scans, carried out along each dihedral. Such soft modes are described by last two terms of equation (S5), i.e. periodic model functions and a minimal set of specific Lennard-Jones (LJ) potentials. To parameterize these latter terms, and complete the intramolecular QMD-FF defined in equation (S5), we minimize the full JOYCE objective function: [11]

$$I = I^H_{g_0} + \sum_{g=0}^{N_{geom}} W_g \left[\Delta U - u^{intra} \right]^2_g \tag{S7}$$

where g runs over the N_{geom} geometries considered along the relaxed scans, and W_g are the standard JOYCE weights [11, 12] Finally, it is here important noticing that, at difference with transferable FFs, which often include all non-bonded intramolecular interactions by default, the JOYCE procedure allows for avoiding such generalized corrections. [11,13] Indeed, the sampled QM PES can be reliably described by solely harmonic functions and/or periodic series, while keeping the contribution of the non-bonded intramolecular term minimal. In the case of flexible polymer chains, however, the reference QM conformational landscape can result quite complex and in some cases driven by intramolecular non-bonded effects, such as internal hydrogen

bonds, π–π stacking, and H–π interactions, that must be explicitly modeled to reproduce the correct QM profile. To this end, a selected and minimal set of specific (LJ) terms is introduced between key atom pairs identified both *via* chemical intuition and QM inspection. This strategy, hereafter referred as specifically selected LJ (*ss*LJ), has been recently introduced [11] in the JOYCE fitting procedure (see the Methods section of Ref. [11], Route II), and found to minimize the structural mismatch with respect to the QM data.

S2.1.2 Polymer chains

Once the QMD-FF for a three unit chain (3**RMU**) is parameterized through the JOYCE procedure as described in the main text and in the previous section, the structure and QMD-FF parameters for an arbitrarily long polymer chain (*n***RMU**) are generated with the CHAMAK code. The software automatically replicates the central unit within the chain, transfers most parameters from the 3**RMU** model and eventually completes the set with possible missing terms. More specifically, CHAMAK generates a GROMACS-compatible topology, containing the *n***RMU** chain structure in the form

$$\mathrm{A}–\mathrm{B}_1–\mathrm{B}_2–\ldots–\mathrm{B}_{(n-2)}–\mathrm{C}$$

where n is the total number of **RMU**s composing the chain, while B_2–…–B_{n-2} are the new building blocks inserted within the A–B_1–C trimer structure (see panel a) of Figure 6 in the main text). Based on the reference 3**RMU** topology, CHAMAK retrieves the QMD-FF parameters for *i*) all harmonic terms of the target *n***RMU**; *ii*) Fourier-like terms for all the flexible dihedrals, including those between two consecutive units; *iii*) the *ss*LJ intramolecular terms describing the interactions between the A–B_1 and B_{n-2}–C pairs. Notably, two important terms, possibly present in longer chains, are instead missing in the above list, namely the interaction between adjacent B units (B_k—B_{k+1}), and the ones among **RMU**s lying far apart in the same chain, whose number of course increases upon chain elongation.

To address these shortcomings, CHAMAK introduces an additional fragmentation layer, distinguishing between two classes of interacting **RMU**s: First Neighboring Units (FNU) or Far-Lying Units (FLU), which respectively indicate adjacent building blocks (A-B_1, B_k—B_{k+1} or

B_{n-2}–C) and units located further apart (e.g. A–B_{k+2}, B_k–B_{k+3}, etc). In FNU, the conformational behavior is driven by a combination of two key contributions, both already tuned along the JOYCE parameterization of the smaller oligomers: the periodic model potentials adopted for flexible dihedrals and the minimal set of *ss*LJ parameters, introduced to capture specific details of the nonbonded interactions established between two adjacent units. To address the first of the two lacking terms in the 3**RMU** model, CHAMAK allows the user to supply the missing data through an external file, which contains ss-LJ parameters transferred from the QMD-FF of the smaller oligomers, or specifically tuned against purposely computed QM data. As the polymer chain grows, the number of specific non-bonded atom pairs that should be identified and managed increases rapidly, making the ss-LJ approach impractical for *n***RMU**'s longer than a decamer. To address this limitation, an alternative methodology is implemented in CHAMAK for handling FLU, which drops the ssLJ strategy in favour of a more straightforward transferability, hence enabling broader applicability to large polymeric systems. In fact, in contrast to FNU, the distinction between intra- and inter-molecular interactions becomes weaker for FLU, and the detail of the local chemical environment less important. Therefore, a more standard strategy was adopted when handling the nonbonded intramolecular term between a pair of FLU contributing to the last term of equation (S5), i.e.

$$u_{ij}^{FLU} = 4\varepsilon_{ij}^{inter}\left[\left(\frac{\sigma_{ij}^{inter}}{r_{ij}}\right)^{12} - \left(\frac{\sigma_{ij}^{inter}}{r_{ij}}\right)^{6}\right] + \frac{q_i q_j}{r_{ij}} \tag{S8}$$

where both LJ parameters and point charges coincide with those adopted in the intermolecular term and i/j run on all atoms of two separate FLU, hence removing the requirement of specific selection. This treatment preserves QMD-FF's specificity for FNU pairs, while enables reliable modeling of polymers of arbitrary length, without the combinatorial explosion of specific pairwise terms. Additionally, the chemically driven fragmentation scheme into FNU and FLU is directly adaptable to CG representations, facilitating consistent control over model accuracy even at reduced resolution.

S2.2 Intermolecular Term

The intermolecular term takes the standard 12-6 Lennard-Jones (LJ) plus charge-charge interactions, i.e.

$$u_{ij}^{inter} = 4\varepsilon_{ij}^{inter}\left[\left(\frac{\sigma_{ij}^{inter}}{r_{ij}}\right)^{12} - \left(\frac{\sigma_{ij}^{inter}}{r_{ij}}\right)^{6}\right] + \frac{q_i q_j}{r_{ij}} \tag{S9}$$

where ε_{ij}^{inter} and σ_{ij}^{inter} are the LJ 12-6 parameters and q_i and q_j the point charges. It is important to stress that the intra- and intermolecular LJ parameter sets $\varepsilon_{ij}^{intra}/\sigma_{ij}^{intra}$ and $\varepsilon_{ij}^{inter}/\sigma_{ij}^{inter}$ may take different values, [10, 13–15] as they describe the interaction of the i and j atoms within the same polymer chain or between two separate chains, respectively. As discussed above, this has important consequences in the parameterization protocol, allowing for more flexible fitting functions, hence potentially increasing the accuracy of the refined FF.

S3 QMD-FF parameters and additional validation

S3.1 Intramolecular

S3.1.1 1RMU Dihedral scans

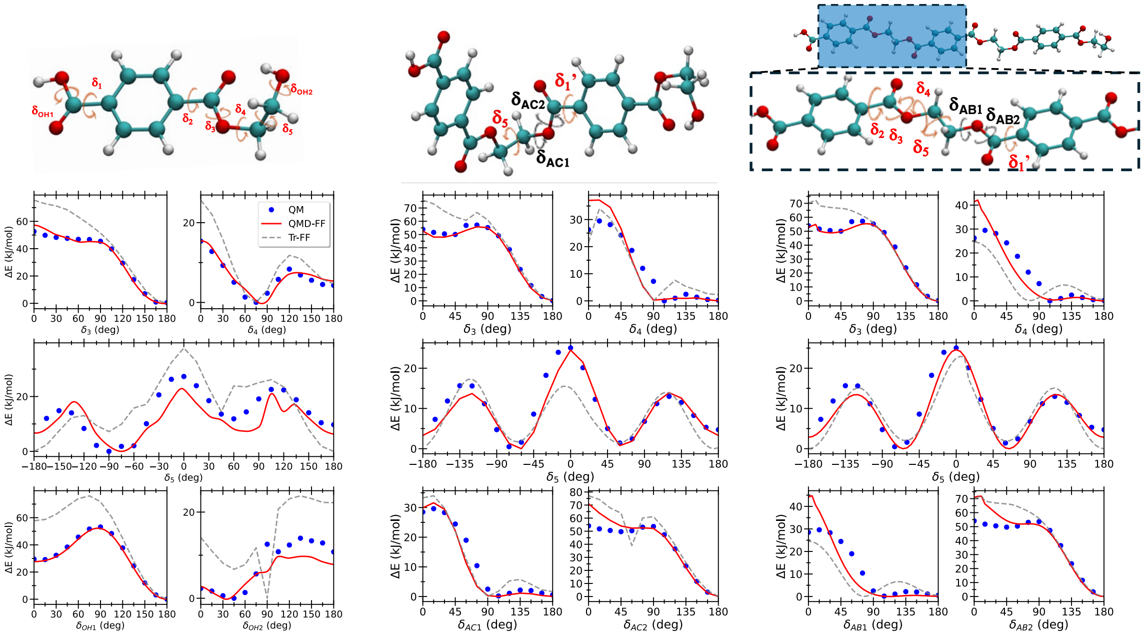

Figure S5: Relaxed dihedral scans of 1**RMU**, 2**RMU**, 3**RMU**

S3.1.2 1RMU QMD-FF parameters

stretching	r^0	k^s	stretching	r^0	k^s
O_{h1}-H_{o1}	0.969	4755.49	O_{h1}-C_{o1}	1.354	3052.01
C_{o1}-O_1	1.206	6407.07	C_{o1}-C_{a1}	1.489	2378.84
C_{a1}-C_{a2}	1.396	2792.95	C_{a2}-C_{a3}	1.385	3181.93
C_{a3}-C_{a4}	1.397	2796.18	C_{a2}-H_{a2}	1.082	3361.80
C_{a3}-H_{a3}	1.081	3372.77	C_{a4}-C_{o2}	1.491	2020.74
C_{o2}-O_2	1.214	6120.81	C_{o2}-O_s	1.338	2715.58
O_s-C_1	1.456	2086.11	C_1-C_2	1.524	2142.57
C_1-H_1	1.091	3150.49	C_2-H_2	1.094	2968.30
C_2-O_{h2}	1.410	2467.33	O_{h2}-H_{o2}	0.969	4476.76

Table S2: Intramolecular QMD-FF stretching parameters obtained for 1**RMU**: equilibrium distances r^0 are in Å and force constants k^s in kJ/mol Å^{-2}.

bending	θ^0	k^b	bending	θ^0	k^b
H_{o1}-O_{h1}-C_{o1}	105.9	499.82	O_{h1}-C_{o1}-O_1	122.3	593.13
O_{h1}-C_{o1}-C_{a1}	113.0	593.13	O_1-C_{o1}-C_{a1}	124.7	593.13
C_{o1}-C_{a1}-C_{a2}	117.9	505.94	C_{a1}-C_{a2}-C_{a3}	120.2	741.76
C_{a2}-C_{a1}-C_{a2}	120.0	541.17	C_{a1}-C_{a2}-H_{a2}	118.6	302.66
C_{a2}-C_{a3}-C_{a4}	119.9	733.43	C_{a3}-C_{a2}-H_{a2}	121.2	348.51
C_{a2}-C_{a3}-H_{a3}	120.5	364.74	C_{a3}-C_{a4}-C_{a3}	119.9	653.23
C_{a4}-C_{a3}-H_{a3}	119.7	294.64	C_{a3}-C_{a4}-C_{o2}	122.2	460.74
C_{a4}-C_{o2}-O_2	123.2	522.29	C_{a4}-C_{o2}-O_s	112.7	522.29
O_2-C_{o2}-O_s	124.1	522.29	C_{o2}-O_s-C_1	118.3	762.46
O_s-C_1-C_2	114.4	1034.90	O_s-C_1-H_1	108.9	495.75
C_2-C_1-H_1	110.1	347.87	C_1-C_2-H_2	107.7	380.84
C_1-C_2-O_{h2}	114.4	818.73	H_1-C_1-H_1	109.8	334.11
H_2-C_2-H_2	107.2	345.96	H_2-C_2-O_{h2}	106.4	461.48
C_2-O_{h2}-H_{o2}	106.9	561.72			

Table S3: Intramolecular QMD-FF bending parameters obtained for 1**RMU**: equilibrium angles θ^0 are in degree force constants k^b in kJ/mol rad^{-2}.

S3.1.3 2RMU QMD-FF parameters

stretching	r^0	k^s	stretching	r^0	k^s
O_{ha}-H_{oa}	0.969	4756.08	O_{ha}-C_{o1a}	1.354	2979.18
C_{o1a}-O_{1a}	1.206	6399.41	C_{o1a}-C_{a1a}	1.489	2390.26
C_{a1a}-C_{a2a}	1.396	2800.60	C_{a2a}-C_{a3a}	1.385	3172.65
C_{a3a}-C_{a4a}	1.397	2825.20	C_{a2a}-H_{a2a}	1.082	3360.21
C_{a3a}-H_{a3a}	1.081	3373.41	C_{a4a}-C_{o2a}	1.492	1974.61
C_{o2a}-O_{2a}	1.206	6403.63	C_{o2a}-O_{sa}	1.354	2620.86
O_{sa}-C_{1a}	1.440	2263.23	C_{1a}-C_{2a}	1.509	2192.82
C_{1a}-H_{1a}	1.089	3164.25	C_{2a}-H_{2a}	1.093	3098.87
C_{2a}-O_{sc}	1.436	2508.09	O_{sc}-C_{o1c}	1.350	2719.91
C_{o1c}-O_{1c}	1.207	6423.20	C_{o1c}-C_{a1c}	1.492	2055.13
C_{a1c}-C_{a2c}	1.396	2932.19	C_{a2c}-C_{a3c}	1.385	3234.13
C_{a3c}-C_{a4c}	1.397	2800.33	C_{a2c}-H_{a2c}	1.082	3348.48
C_{a3c}-H_{a3c}	1.081	3378.30	C_{a4c}-C_{o2c}	1.490	2106.85
C_{o2c}-O_{2c}	1.214	6118.27	C_{o2c}-O_{sc}	1.339	2772.22
O_{sc}-C_{1c}	1.456	1999.18	C_{1c}-C_{2c}	1.524	2124.09
C_{1c}-H_{1c}	1.091	3148.80	C_{2c}-H_{2c}	1.094	2967.11
C_{2c}-O_{hc}	1.410	2478.37	O_{hc}-H_{oc}	0.969	4635.99

Table S4: Intramolecular QMD-FF stretching parameters obtained for 2**RMU**: equilibrium distances r^0 are in Å and force constants k^s in kJ/mol Å^{-2}.

**bending**	θ^0	$\mathbf{k^b}$	**bending**	θ^0	$\mathbf{k^b}$
H_{oa}-O_{ha}-C_{o1a}	105.9	499.26	O_{ha}-C_{o1a}-O_{1a}	122.3	607.15
O_{ha}-C_{o1a}-C_{a1a}	113.0	607.15	O_{1a}-C_{o1a}-C_{a1a}	124.7	607.15
C_{o1a}-C_{a1a}-C_{a2a}	117.9	500.92	C_{a1a}-C_{a2a}-C_{a3a}	120.2	744.39
C_{a2a}-C_{a1a}-C_{a2a}	120.0	505.49	C_{a1a}-C_{a2a}-H_{a2a}	118.6	308.13
C_{a2a}-C_{a3a}-C_{a4a}	119.9	717.71	C_{a3a}-C_{a2a}-H_{a2a}	121.2	345.27
C_{a2a}-C_{a3a}-H_{a3a}	120.4	352.46	C_{a3a}-C_{a4a}-C_{a3a}	119.9	620.80
C_{a4a}-C_{a3a}-H_{a3a}	119.7	304.15	C_{a3a}-C_{a4a}-C_{o2a}	122.5	486.44
C_{a4a}-C_{o2a}-O_{2a}	124.0	488.96	C_{a4a}-C_{o2a}-O_{sa}	112.0	488.96
O_{2a}-C_{o2a}-O_{sa}	124.1	488.96	C_{o2a}-O_{sa}-C_{1a}	117.5	888.93
O_{sa}-C_{1a}-C_{2a}	110.5	773.39	O_{sa}-C_{1a}-H_{1a}	109.4	472.37
C_{2a}-C_{1a}-H_{1a}	111.2	403.41	C_{1a}-C_{2a}-H_{2a}	110.2	370.24
C_{1a}-C_{2a}-O_{sc}	107.6	774.11	H_{1a}-C_{1a}-H_{1a}	110.1	318.06
H_{2a}-C_{2a}-H_{2a}	108.3	321.34	H_{2a}-C_{2a}-O_{sc}	109.7	486.37
C_{2a}-O_{sc}-C_{o1c}	116.0	789.62	O_{sc}-C_{o1c}-O_{1c}	123.3	520.31
O_{sc}-C_{o1c}-C_{a1c}	112.1	520.31	O_{1c}-C_{o1c}-C_{a1c}	124.6	520.31
C_{o1c}-C_{a1c}-C_{a2c}	117.8	514.32	C_{a1c}-C_{a2c}-C_{a3c}	120.2	693.36
C_{a2c}-C_{a1c}-C_{a2c}	120.0	593.19	C_{a1c}-C_{a2c}-H_{a2c}	118.6	293.73
C_{a2c}-C_{a3c}-C_{a4c}	119.8	757.14	C_{a3c}-C_{a2c}-H_{a2c}	121.2	346.67
C_{a2c}-C_{a3c}-H_{a3c}	120.5	363.86	C_{a3c}-C_{a4c}-C_{a3c}	119.9	604.23
C_{a4c}-C_{a3c}-H_{a3c}	119.7	299.40	C_{a3c}-C_{a4c}-C_{o2c}	122.2	351.91
C_{a4c}-C_{o2c}-O_{2c}	123.3	544.09	C_{a4c}-C_{o2c}-O_{sc}	112.8	544.09
O_{2c}-C_{o2c}-O_{sc}	124.0	544.09	C_{o2c}-O_{sc}-C_{1c}	118.3	740.10
O_{sc}-C_{1c}-C_{2c}	114.4	991.25	O_{sc}-C_{1c}-H_{1c}	108.9	497.79
C_{2c}-C_{1c}-H_{1c}	110.1	346.54	C_{1c}-C_{2c}-H_{2c}	107.8	380.60
C_{1c}-C_{2c}-O_{hc}	114.4	773.84	H_{1c}-C_{1c}-H_{1c}	109.7	333.65
H_{2c}-C_{2c}-H_{2c}	107.2	346.59	H_{2c}-C_{2c}-O_{hc}	106.4	460.65
C_{2c}-O_{hc}-H_{oc}	106.9	594.02			

Table S5: Intramolecular bending parameters obtained for 2**RMU**: equilibrium angles θ^0 are in degree force constants k^b in kJ/mol rad^{-2}.

dihedral	ϕ^0	k^t	dihedral	ϕ^0	k^t
C_{a1a}-C_{a2a}-C_{a3a}-C_{a4a}	0.0	84.00	C_{a2a}-C_{a1a}-C_{a2a}-C_{a3a}	0.0	84.00
C_{a2a}-C_{a3a}-C_{a4a}-C_{a3a}	0.0	84.00	C_{a1c}-C_{a2c}-C_{a3c}-C_{a4c}	0.0	84.00
C_{a2c}-C_{a1c}-C_{a2c}-C_{a3c}	0.0	84.00	C_{a2c}-C_{a3c}-C_{a4c}-C_{a3c}	0.0	84.00
C_{o1a}-C_{a1a}-C_{a2a}-C_{a3a}	180.0	84.00	C_{a2a}-C_{a3a}-C_{a4a}-C_{o2a}	180.0	84.00
C_{o1c}-C_{a1c}-C_{a2c}-C_{a3c}	180.0	84.00	C_{a2c}-C_{a3c}-C_{a4c}-C_{o2c}	180.0	84.00
C_{a1a}-C_{a2a}-C_{a3a}-H_{a3a}	180.0	70.37	C_{a2a}-C_{a1a}-C_{a2a}-H_{a2a}	180.0	70.37
C_{a3a}-C_{a4a}-C_{a3a}-H_{a3a}	180.0	70.37	C_{a4a}-C_{a3a}-C_{a2a}-H_{a2a}	180.0	70.37
C_{a2c}-C_{a1c}-C_{a2c}-H_{a2c}	180.0	70.37	H_{a2c}-C_{a2c}-C_{a3c}-C_{a4c}	180.0	70.37
H_{a3c}-C_{a3c}-C_{a4c}-C_{a3c}	180.0	70.37	C_{a1c}-C_{a2c}-C_{a3c}-H_{a3c}	180.0	70.37
C_{o1a}-C_{a1a}-C_{a2a}-H_{a2a}	0.0	33.76	H_{a3a}-C_{a3a}-C_{a4a}-C_{o2a}	0.0	33.76
H_{a2a}-C_{a2a}-C_{a3a}-H_{a3a}	0.0	33.76	C_{o1c}-C_{a1c}-C_{a2c}-H_{a2c}	0.0	33.76
H_{a3c}-C_{a3c}-C_{a2c}-H_{a2c}	0.0	33.76	H_{a3c}-C_{a3c}-C_{a4c}-C_{o2c}	0.0	33.76
C_{a1a}-C_{o1a}-C_{a2a}-C_{a2a}	0.0	84.00	C_{o2a}-C_{a3a}-C_{a3a}-C_{a4a}	0.0	84.00
C_{a1c}-C_{o1c}-C_{a2c}-C_{a2c}	0.0	84.00	C_{o2c}-C_{a3c}-C_{a3c}-C_{a4c}	0.0	84.00
C_{o1a}-O_{ha}-O_{1a}-C_{a1a}	0.0	84.00	H_{a2a}-C_{a1a}-C_{a3a}-C_{a2a}	0.0	70.37
H_{a3a}-C_{a2a}-C_{a4a}-C_{a3a}	0.0	70.37	C_{o2a}-C_{a4a}-O_{2a}-O_{sa}	0.0	84.00
C_{o1c}-O_{sc}-O_{1c}-C_{a1c}	0.0	84.00	H_{a2c}-C_{a1c}-C_{a3c}-C_{a2c}	0.0	70.37
H_{a3c}-C_{a2c}-C_{a4c}-C_{a3c}	0.0	70.37	C_{o2c}-C_{a4c}-O_{2c}-O_{sc}	0.0	84.00

Table S6: Intramolecular parameters for stiff torsions obtained for 2**RMU**: equilibrium dihedral angles ϕ^0 are in degrees and force constants k^t in kJ/mol rad^{-2}.

dihedral	N_{cos}	n	k^d (kJ/mol)	γ (degr)
H_{oa}-O_{ha}-C_{o1a}-C_{a1a}	6	0	4.721	0.00
		1	11.627	0.00
		2	-19.119	0.00
		3	2.107	0.00
		4	1.537	0.00
		5	0.155	0.00
O_{ha}-C_{o1a}-C_{a1a}-C_{a2a}	4	0	4.721	0.00
		2	-7.014	0.00
		4	0.440	0.00
		6	0.154	0.00
O_{1a}-C_{o1a}-C_{a1a}-C_{a2a}	4	0	4.721	0.00
		2	-7.014	0.00
		4	0.440	0.00
		6	0.154	0.00
C_{a3a}-C_{a4a}-C_{o2a}-O_{sa}	4	0	4.721	0.00
		2	-6.850	0.00
		4	0.506	0.00
		6	0.137	0.00

C_{a3a}-C_{a4a}-C_{o2a}-O_{2a}	4	0	4.721	0.00
		2	-6.850	0.00
		4	0.506	0.00
		6	0.137	0.00
C_{a4a}-C_{o2a}-O_{sa}-C_{1a}	6	0	4.721	0.00
		1	16.072	0.00
		2	-18.805	0.00
		3	0.936	0.00
		4	1.325	0.00
		5	-0.356	0.00
C_{o2a}-O_{sa}-C_{1a}-C_{2a}	6	0	4.721	0.00
		1	9.989	0.00
		2	4.610	0.00
		3	0.917	0.00
		4	-0.385	0.00
		5	0.413	0.00
O_{sa}-C_{1a}-C_{2a}-O_{sc}	6	0	4.721	0.00
		1	3.648	0.00
		2	4.244	0.00
		3	8.231	0.00
		4	0.886	0.00
		5	0.097	0.00
C_{1a}-C_{2a}-O_{sc}-C_{o1c}	6	0	4.721	0.00
		1	10.317	0.00
		2	5.810	0.00
		3	2.013	0.00
		4	0.387	0.00
		5	0.253	0.00
C_{2a}-O_{sc}-C_{o1c}-C_{a1c}	6	0	4.721	0.00
		1	22.026	0.00
		2	-12.761	0.00
		3	3.608	0.00
		4	1.634	0.00
		5	-0.329	0.00
O_{sc}-C_{o1c}-C_{a1c}-C_{a2c}	4	0	4.721	0.00
		2	-6.487	0.00
		4	0.401	0.00
		6	0.149	0.00
O_{1c}-C_{o1c}-C_{a1c}-C_{a2c}	4	0	4.721	0.00
		2	-6.487	0.00
		4	0.401	0.00
		6	0.149	0.00
C_{a3c}-C_{a4c}-C_{o2c}-O_{sc}	4	0	4.721	0.00

		2	-6.929	0.00
		4	0.495	0.00
		6	0.140	0.00
C_{a3c}-C_{a4c}-C_{o2c}-O_{2c}	4	0	4.721	0.00
		2	-6.929	0.00
		4	0.495	0.00
		6	0.140	0.00
C_{a4c}-C_{o2c}-O_{sc}-C_{1c}	6	0	4.721	0.00
		1	14.007	0.00
		2	-15.009	0.00
		3	1.722	0.00
		4	1.707	0.00
		5	-1.077	0.00
C_{o2c}-O_{sc}-C_{1c}-C_{2c}	6	0	4.721	0.00
		1	5.279	0.00
		2	2.252	0.00
		3	2.481	0.00
		4	0.964	0.00
		5	0.519	0.00
O_{sc}-C_{1c}-C_{2c}-O_{hc}	6	0	4.721	0.00
		1	5.429	0.00
		2	4.463	0.00
		3	5.439	0.00
		4	-0.643	0.00
		5	0.657	0.00
C_{1c}-C_{2c}-O_{hc}-H_{oc}	6	0	4.721	0.00
		1	0.024	0.00
		2	-1.199	0.00
		3	0.566	0.00
		4	0.577	0.00
		5	0.886	0.00

Table S7: Intramolecular parameters for flexible torsions obtained for 2**RMU**: number of cosines n, γ (degrees) and force constants k^d in kJ/mol.

S3.1.4 3**RMU** QMD-FF parameters

stretching	r^0	k^s	stretching	r^0	k^s
O_{ha}-H_{oa}	0.969	4756.06	O_{ha}-C_{o1a}	1.354	3137.98
C_{o1a}-O_{1a}	1.206	6572.11	C_{o1a}-C_{a1a}	1.489	2215.38
C_{a1a}-C_{a2a}	1.396	2756.85	C_{a2a}-C_{a3a}	1.385	3136.08
C_{a3a}-C_{a4a}	1.397	2828.47	C_{a2a}-H_{a2a}	1.082	3362.28
C_{a3a}-H_{a3a}	1.081	3369.13	C_{a4a}-C_{o2a}	1.491	2007.94
C_{o2a}-O_{2a}	1.206	6452.88	C_{o2a}-O_{sa}	1.352	2657.61
O_{sa}-C_{1a}	1.438	2358.56	C_{1a}-C_{2a}	1.514	2255.96
C_{1a}-H_{1a}	1.091	3123.11	C_{2a}-H_{2a}	1.091	3123.42
C_{2a}-O_{sb}	1.438	2402.11	O_{sb}-C_{o1b}	1.352	2754.72
C_{o1b}-O_{1b}	1.206	6422.53	C_{o1b}-C_{a1b}	1.491	2067.67
C_{a1b}-C_{a2b}	1.396	2820.10	C_{a2b}-C_{a3b}	1.385	3160.55
C_{a3b}-C_{a4b}	1.397	2811.74	C_{a2b}-H_{a2b}	1.082	3364.97
C_{a3b}-H_{a3b}	1.081	3367.42	C_{a4b}-C_{o2b}	1.491	2024.68
C_{o2b}-O_{2b}	1.206	6440.78	C_{o2b}-O_{sb}	1.352	2705.68
O_{sb}-C_{1b}	1.439	2314.90	C_{1b}-C_{2b}	1.518	2240.73
C_{1b}-H_{1b}	1.090	3126.75	C_{2b}-H_{2b}	1.089	3181.42
C_{2b}-O_{sc}	1.439	2388.37	O_{sc}-C_{o1c}	1.355	2581.76
C_{o1c}-O_{1c}	1.206	6432.96	C_{o1c}-C_{a1c}	1.491	2012.99
C_{a1c}-C_{a2c}	1.396	2802.69	C_{a2c}-C_{a3c}	1.386	3131.94
C_{a3c}-C_{a4c}	1.396	2816.29	C_{a2c}-H_{a2c}	1.081	3366.82
C_{a3c}-H_{a3c}	1.081	3370.29	C_{a4c}-C_{o2c}	1.491	2102.90
C_{o2c}-O_{2c}	1.214	6109.27	C_{o2c}-O_{sc}	1.338	2792.95
O_{sc}-C_{1c}	1.456	2004.51	C_{1c}-C_{2c}	1.524	2090.45
C_{1c}-H_{1c}	1.091	3150.87	C_{2c}-H_{2c}	1.094	2967.66
C_{2c}-O_{hc}	1.410	2506.21	O_{hc}-H_{oc}	0.969	4646.73

Table S8: Intramolecular stretching parameters obtained for 3**RMU**: equilibrium distances r^0 are in Å and force constants k^s in kJ/mol Å^{-2}.

bending	θ^0	k^b	bending	θ^0	k^b
H_{oa}-O_{ha}-C_{o1a}	105.9	495.98	O_{ha}-C_{o1a}-O_{1a}	122.3	1095.13
O_{ha}-C_{o1a}-C_{a1a}	113.0	131.90	O_{1a}-C_{o1a}-C_{a1a}	124.7	131.90
C_{o1a}-C_{a1a}-C_{a2a}	117.9	770.89	C_{a1a}-C_{a2a}-C_{a3a}	120.2	668.14
C_{a2a}-C_{a1a}-C_{a2a}	120.0	517.29	C_{a1a}-C_{a2a}-H_{a2a}	118.6	291.55
C_{a2a}-C_{a3a}-C_{a4a}	119.9	768.39	C_{a3a}-C_{a2a}-H_{a2a}	121.2	360.24
C_{a2a}-C_{a3a}-H_{a3a}	120.3	364.99	C_{a3a}-C_{a4a}-C_{a3a}	119.9	641.65
C_{a4a}-C_{a3a}-H_{a3a}	119.8	292.51	C_{a3a}-C_{a4a}-C_{o2a}	122.5	432.17
C_{a4a}-C_{o2a}-O_{2a}	124.5	518.17	C_{a4a}-C_{o2a}-O_{sa}	112.5	518.17
O_{2a}-C_{o2a}-O_{sa}	123.0	518.17	C_{o2a}-O_{sa}-C_{1a}	115.2	913.47
O_{sa}-C_{1a}-C_{2a}	106.0	617.24	O_{sa}-C_{1a}-H_{1a}	109.8	483.91
C_{2a}-C_{1a}-H_{1a}	111.5	395.58	C_{1a}-C_{2a}-H_{2a}	111.5	396.53
C_{1a}-C_{2a}-O_{sb}	106.0	626.14	H_{1a}-C_{1a}-H_{1a}	108.2	326.74
H_{2a}-C_{2a}-H_{2a}	108.2	326.41	H_{2a}-C_{2a}-O_{sb}	109.8	485.28
C_{2a}-O_{sb}-C_{o1b}	115.3	885.91	O_{sb}-C_{o1b}-O_{1b}	123.0	518.70
O_{sb}-C_{o1b}-C_{a1b}	112.5	518.70	O_{1b}-C_{o1b}-C_{a1b}	124.4	518.70
C_{o1b}-C_{a1b}-C_{a2b}	117.7	465.16	C_{a1b}-C_{a2b}-C_{a3b}	120.2	732.64
C_{a2b}-C_{a1b}-C_{a2b}	119.9	615.00	C_{a1b}-C_{a2b}-H_{a2b}	118.6	301.50
C_{a2b}-C_{a3b}-C_{a4b}	119.9	729.18	C_{a3b}-C_{a2b}-H_{a2b}	121.2	361.17
C_{a2b}-C_{a3b}-H_{a3b}	120.3	362.00	C_{a3b}-C_{a4b}-C_{a3b}	119.8	620.56
C_{a4b}-C_{a3b}-H_{a3b}	119.7	301.44	C_{a3b}-C_{a4b}-C_{o2b}	122.5	463.37
C_{a4b}-C_{o2b}-O_{2b}	124.4	523.15	C_{a4b}-C_{o2b}-O_{sb}	112.5	523.15
O_{2b}-C_{o2b}-O_{sb}	123.1	523.15	C_{o2b}-O_{sb}-C_{1b}	115.3	905.27
O_{sb}-C_{1b}-C_{2b}	105.9	619.85	O_{sb}-C_{1b}-H_{1b}	109.7	488.10
C_{2b}-C_{1b}-H_{1b}	111.3	373.19	C_{1b}-C_{2b}-H_{2b}	110.8	427.12
C_{1b}-C_{2b}-O_{sc}	109.8	792.74	H_{1b}-C_{1b}-H_{1b}	108.7	325.86
H_{2b}-C_{2b}-H_{2b}	110.1	319.65	H_{2b}-C_{2b}-O_{sc}	105.5	466.54
C_{2b}-O_{sc}-C_{o1c}	116.3	931.01	O_{sc}-C_{o1c}-O_{1c}	123.5	494.07
O_{sc}-C_{o1c}-C_{a1c}	112.3	494.07	O_{1c}-C_{o1c}-C_{a1c}	124.2	494.07
C_{o1c}-C_{a1c}-C_{a2c}	122.4	496.05	C_{a1c}-C_{a2c}-C_{a3c}	120.0	722.24
C_{a2c}-C_{a1c}-C_{a2c}	119.9	600.50	C_{a1c}-C_{a2c}-H_{a2c}	119.7	305.25
C_{a2c}-C_{a3c}-C_{a4c}	120.0	737.12	C_{a3c}-C_{a2c}-H_{a2c}	120.3	359.41
C_{a2c}-C_{a3c}-H_{a3c}	120.3	360.44	C_{a3c}-C_{a4c}-C_{a3c}	119.9	613.77
C_{a4c}-C_{a3c}-H_{a3c}	119.6	303.52	C_{a3c}-C_{a4c}-C_{o2c}	122.2	460.44
C_{a4c}-C_{o2c}-O_{2c}	123.2	523.08	C_{a4c}-C_{o2c}-O_{sc}	112.7	523.08
O_{2c}-C_{o2c}-O_{sc}	124.1	523.08	C_{o2c}-O_{sc}-C_{1c}	118.3	761.01
O_{sc}-C_{1c}-C_{2c}	114.4	1016.51	O_{sc}-C_{1c}-H_{1c}	108.9	497.67
C_{2c}-C_{1c}-H_{1c}	110.1	345.33	C_{1c}-C_{2c}-H_{2c}	107.7	381.18

C_{1c}-C_{2c}-O_{hc}	114.4	865.48	H_{1c}-C_{1c}-H_{1c}	109.8	333.60
H_{2c}-C_{2c}-H_{2c}	107.2	345.99	H_{2c}-C_{2c}-O_{hc}	106.4	461.80
C_{2c}-O_{hc}-H_{oc}	107.0	592.07			

Table S9: Intramolecular bending parameters obtained for 3**RMU**: equilibrium angles θ^0 are in degree force constants k^b in kJ/mol rad^{-2}.

dihedral	ϕ^0	k^t	dihedral	ϕ^0	k^t
C_{a1a}-C_{a2a}-C_{a3a}-C_{a4a}	0.0	114.77	C_{a2a}-C_{a1a}-C_{a2a}-C_{a3a}	0.0	114.77
C_{a2a}-C_{a3a}-C_{a4a}-C_{a3a}	0.0	114.77	C_{a1b}-C_{a2b}-C_{a3b}-C_{a4b}	0.0	114.77
C_{a2b}-C_{a1b}-C_{a2b}-C_{a3b}	0.0	114.77	C_{a2b}-C_{a3b}-C_{a4b}-C_{a3b}	0.0	114.77
C_{a1c}-C_{a2c}-C_{a3c}-C_{a4c}	0.0	114.77	C_{a2c}-C_{a3c}-C_{a4c}-C_{a3c}	0.0	114.77
C_{a2c}-C_{a1c}-C_{a2c}-C_{a3c}	0.0	114.77	C_{o1a}-C_{a1a}-C_{a2a}-C_{a3a}	180.0	114.77
C_{a2a}-C_{a3a}-C_{a4a}-C_{o2a}	180.0	114.77	C_{o1b}-C_{a1b}-C_{a2b}-C_{a3b}	180.0	114.77
C_{a2b}-C_{a3b}-C_{a4b}-C_{o2b}	180.0	114.77	C_{o1c}-C_{a1c}-C_{a2c}-C_{a3c}	180.0	114.77
C_{a2c}-C_{a3c}-C_{a4c}-C_{o2c}	180.0	114.77	C_{a1a}-C_{a2a}-C_{a3a}-H_{a3a}	180.0	75.59
C_{a2a}-C_{a1a}-C_{a2a}-H_{a2a}	180.0	75.59	C_{a3a}-C_{a4a}-C_{a3a}-H_{a3a}	180.0	75.59
C_{a4a}-C_{a3a}-C_{a2a}-H_{a2a}	180.0	75.59	C_{a1b}-C_{a2b}-C_{a3b}-H_{a3b}	180.0	75.59
C_{a2b}-C_{a1b}-C_{a2b}-H_{a2b}	180.0	75.59	C_{a3b}-C_{a4b}-C_{a3b}-H_{a3b}	180.0	75.59
C_{a4b}-C_{a3b}-C_{a2b}-H_{a2b}	180.0	75.59	C_{a2c}-C_{a1c}-C_{a2c}-H_{a2c}	180.0	75.59
H_{a2c}-C_{a2c}-C_{a3c}-C_{a4c}	180.0	75.59	H_{a3c}-C_{a3c}-C_{a4c}-C_{a3c}	180.0	75.59
H_{a3c}-C_{a3c}-C_{a2c}-C_{a1c}	180.0	75.59	C_{o1a}-C_{a1a}-C_{a2a}-H_{a2a}	0.0	28.23
H_{a3a}-C_{a3a}-C_{a4a}-C_{o2a}	0.0	28.23	H_{a2a}-C_{a2a}-C_{a3a}-H_{a3a}	0.0	28.23
C_{o1b}-C_{a1b}-C_{a2b}-H_{a2b}	0.0	28.23	H_{a3b}-C_{a3b}-C_{a4b}-C_{o2b}	0.0	28.23
H_{a2b}-C_{a2b}-C_{a3b}-H_{a3b}	0.0	28.23	C_{o1c}-C_{a1c}-C_{a2c}-H_{a2c}	0.0	28.23
H_{a3c}-C_{a3c}-C_{a2c}-H_{a2c}	0.0	28.23	H_{a3c}-C_{a3c}-C_{a4c}-C_{o2c}	0.0	28.23
C_{a1a}-C_{o1a}-C_{a2a}-C_{a2a}	0.0	114.77	C_{o2a}-C_{a3a}-C_{a3a}-C_{a4a}	0.0	114.77
C_{a1c}-C_{o1c}-C_{a2c}-C_{a2c}	0.0	114.77	C_{o2c}-C_{a3c}-C_{a3c}-C_{a4c}	0.0	114.77
C_{a1b}-C_{o1b}-C_{a2b}-C_{a2b}	0.0	114.77	C_{o2b}-C_{a3b}-C_{a3b}-C_{a4b}	0.0	114.77
C_{o1a}-O_{ha}-O_{1a}-C_{a1a}	0.0	114.77	H_{a2a}-C_{a1a}-C_{a3a}-C_{a2a}	0.0	75.59
H_{a3a}-C_{a2a}-C_{a4a}-C_{a3a}	0.0	75.59	C_{o2a}-C_{a4a}-O_{2a}-O_{sa}	0.0	114.77
C_{o1b}-O_{sb}-O_{1b}-C_{a1b}	0.0	114.77	H_{a2b}-C_{a1b}-C_{a3b}-C_{a2b}	0.0	75.59
H_{a3b}-C_{a2b}-C_{a4b}-C_{a3b}	0.0	75.59	C_{o2b}-C_{a4b}-O_{2b}-O_{sb}	0.0	114.77
C_{o1c}-O_{sc}-O_{1c}-C_{a1c}	0.0	114.77	H_{a2c}-C_{a1c}-C_{a3c}-C_{a2c}	0.0	75.59
H_{a3c}-C_{a2c}-C_{a4c}-C_{a3c}	0.0	75.59	C_{o2c}-C_{a4c}-O_{2c}-O_{sc}	0.0	114.77

Table S10: Intramolecular parameters for stiff torsions obtained for 3**RMU**: equilibrium dihedral angles ϕ^0 are in degrees and force constants k^t in kJ/mol rad^{-2}.

S3.2 Intermolecular

S3.2.1 PICKY parameterization

The PICKY procedure [2, 14–16] is an automated iterative parameterization protocol, based on the optimization of an objective function similar to the one defined for JOYCE, i.e.

$$I^{inter} = \frac{1}{C}\sum_{i}^{N_{dim}} W_i \left[\Delta U^{QM} - E^{FF}_{inter}\right]^2_i \; ; \; C = \sum_{i}^{N_{dim}} W_i \tag{S10}$$

where N_{dim} is the number of sampled geometrical arrangements of two noncovalently interacting molecules, W_i a Boltzmann-like weight and ΔU^{QM} the intermolecular energy computed at the selected geometry at a proper QM level (here B3LYP-D3BJ/6-311G(d,p), see section S1). A QM reference database, containing the sampled geometries of a pair of 4**RMU** chains and their interaction energy, is progressively enlarged across successive PICKY's cycles. Each cycle **c** consists in the five steps:

c.0) A system composed by 216 4**RMU** chains is equilibrated through NPT-MD simulations, initially carried out with the Hy-FF.

c.I) The PICKY selection algorithm [14] is employed to extract 50 to 100 different chain pairs from the MD trajectory carried out in the previous cycle.

c.II) The interaction energy ΔE^{QM} of all 4**RMU** pairs, selected in step **c**.I, is computed at B3LYP-D3BJ/6-311G(d,p) level. The FRM scheme, [17,18] implemented in the PICKY3.0 version of the code [19] is employed to speed up the calculations. Details on the applied FRM scheme and its accuracy are dispalyed in Figure S6.

c.III) The inter-molecular parameters are obtained by minimizing the objective function I^{inter}.

c.IV) The convergence of the iterative protocol is monitored by means of ΔP, which measures the difference between the QMD-FF IPESs obtained in two consecutive cycles as

$$\Delta P_c = \left(\frac{1}{N_{conf}}\sum_{i}^{N_{conf}} \left[(\Delta E^{FF}_{inter})_c - (\Delta E_i^{FF_{inter}})_{c-1}\right]^2_i\right)^{\frac{1}{2}} \tag{S11}$$

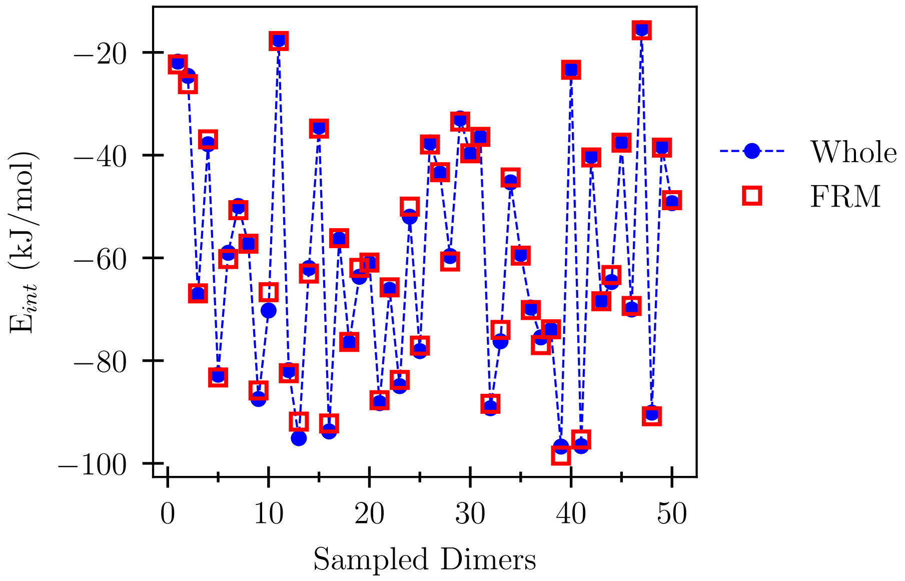

Figure S6: Comparison of the interaction energy if the 4**RMU** pairs computed considering the the whole chain pairs (blue line and symbols) and the one obtained using the FRM method (red squares), the mean absolute error is estimated to be 0.85 kJ/mol

where the index i runs over a grid (with $N_{conf} > 10^6$) in the six dimensional configurational space spanned by translating and rotating one chain with respect to the other and ΔE_{inter}^{FF} is the FF dimer interaction energy computed using the PICKY inter-molecular parameters obtained at cycle **c** and **c-1**, respectively.

c.V) If the procedure has not yet converged, the inter-molecular parameters found in step **c**.III) are employed in a further MD run, carried out again in the NPT ensemble, starting from the final configuration of the simulation performed in step **c**I). The trajectory of this run is then used to extract new dimers, thus repeating step I in the next PICKY's cycle (**c+1**).

The detailed protocol adopted for the PICKY parameterization of a 4**RMU** is shown in detail in Table S11.

Cycle	Step	Details
**0**	V	$\sim$ 100 ns JOYCE/OPLS run at 380 K, isotropic phase
**1**	I	50 dimers selected
		R_{max}=7, E_{max}=10, E_{min}=-100, ΔI_{tr}=5, ΔI_w=25/25/25/25
	II	50 FRM reconstructed energies with CP correction
	III	fit with 10% max variance on LJ selected parameters and α=0.05
	IV	ΔP evaluated on $>10^6$ points, on a 7 Å radius sphere
	V	1 ns MD run at 300 K with cycle **1** parameters
**2**	I	100 dimers
		A: R_{max}=10, E_{max}=-1, E_{min}=-200, ΔI_{tr}=10, ΔI_w=25/25/25/25
		R: R_{max}=15-18, E_{max}=100, E_{min}=-1, ΔI_{tr}=5, ΔI_w=25/25/25/25
	II	100 FRM reconstructed energies with CP correction
	V	1 ns MD run at 300 K with with cycle **2** parameters
**3**	I	100 dimers
		A: R_{max}=9, E_{max}=-1, E_{min}=-200, ΔI_{tr}=10-8, ΔI_w=50/0/20/30
		R: R_{max}=10, E_{max}=100, E_{min}=-1, ΔI_{tr}=4-8, ΔI_w=50/0/40/10
	V	1 ns MD run at 350 K with with cycle **3** parameters
**4**	I	100 dimers
		A: R_{max}=10, E_{max}=-50, E_{min}=-300, ΔI_{tr}=8, ΔI_w=25/25/0/50
		R: R_{max}=25, E_{max}=100, E_{min}=0.1, ΔI_{tr}=4, ΔI_w=0/25/25/50
	III	fit with 100% max variance on LJ selected parameters and α=0.05
	V	1 ns MD run at 350 K with with cycle **4** parameters

Table S11: Details of the PICKY parameterization protocol applied of 4**RMU** tetramer. In all PICKY cycles, during the sampling step I, dimers were extracted by sampling both attractive (A) and repulsive (R) regions, addressing the search through specific parameters: R_{max} is the maximum allowed distance between the monomer centers of mass, $E_{max/min}$ (in kJ/mol) is the maximum/minimum allowed interaction energy, ΔI_{tr} is the lowest allowed differentiality index (ΔI) [2, 14] and ΔI_w indicates the weights (percentage) for the different contributions [2, 14, 16] In the steps not described, the procedure is identical to the one adopted on the preceding cycle.

S3.2.2 *n*RMU QMD-FF parameters

	Tr-FF			QMD-FF		
Atom type	σ	ε	q	σ	ε	q
O_{ha}	3.120	0.7113	-0.5957	2.779	0.2268	-0.5957
H_{oa}	0.000	0.0000	0.4510	2.000	0.0050	0.4510
C_{o1a}	3.550	0.2929	0.6839	4.683	0.0181	0.6839
O_{1a}	2.960	0.8786	-0.5850	2.817	0.6055	-0.5850
C_{a1a}	3.550	0.2929	-0.0197	2.720	2.4622	-0.0197
C_{a2a}	3.550	0.2929	-0.1091	3.239	1.3571	-0.1091
C_{a3a}	3.550	0.2929	-0.1154	3.519	0.1390	-0.1154
C_{a4a}	3.550	0.2929	-0.1205	2.000	1.8309	-0.1205
H_{a2a}	2.420	0.1255	0.1464	2.480	0.0584	0.1464
H_{a3a}	2.420	0.1255	0.1338	2.000	0.0148	0.1338
C_{o2a}	3.550	0.2929	0.8285	3.795	0.1046	0.8285
O_{2a}	2.960	0.8786	-0.6031	3.201	0.0584	-0.6031
O_{sa}	2.900	0.5858	-0.4626	3.676	0.1578	-0.4626
C_{1a}	3.500	0.2761	0.1144	3.778	0.6432	0.1144
C_{2a}	3.500	0.2761	0.1286	2.871	1.9068	0.1286
H_{1a}	2.500	0.1255	0.0871	2.000	0.0634	0.0871
H_{2a}	2.500	0.1255	0.0862	3.143	0.0050	0.0862

Table S12: Intermolecular parameters for *n*RMU PET chains: head unit (A, see main text). σ (Å), ε (kJ/mol) and q (e^-) Tr-FF parameters (OPLS [20,21]) are reported in the first columns for comparison.

Atom type	Tr-FF			QMD-FF		
	σ	ε	q	σ	ε	q
O_{sb}	2.900	0.5858	-0.4152	2.958	0.6302	-0.4152
C_{o1b}	3.550	0.2929	0.8082	3.097	0.3804	0.8082
O_{1b}	2.960	0.8786	-0.5987	2.987	0.4440	-0.5987
C_{a1b}	3.550	0.2929	-0.1282	3.190	0.6522	-0.1282
C_{a2b}	3.550	0.2929	-0.0888	3.575	0.1470	-0.0888
C_{a3b}	3.550	0.2929	-0.0915	3.243	0.4569	-0.0915
C_{a4b}	3.550	0.2929	-0.1263	3.230	1.1522	-0.1263
H_{a2b}	2.420	0.1255	0.1317	2.639	0.0110	0.1317
H_{a3b}	2.420	0.1255	0.1306	2.578	0.0157	0.1306
C_{o2b}	3.550	0.2929	0.8063	3.868	0.1335	0.8063
O_{2b}	2.960	0.8786	-0.5961	2.605	2.0055	-0.5961
C_{1b}	3.500	0.2761	0.0456	3.399	0.2685	0.0456
C_{2b}	3.500	0.2761	0.0445	3.750	0.6073	0.0445
H_{1b}	2.500	0.1255	0.1096	2.863	0.0050	0.1096
H_{2b}	2.500	0.1255	0.1132	2.769	0.0050	0.1132

Table S13: Intermolecular parameters for *n***RMU** PET chains: central repeating units (B_n, see main text). σ (Å), ε (kJ/mol) and q (e^-) Tr-FF parameters (OPLS [20, 21]) are reported in the first columns for comparison.

	Tr-FF			QMD-FF		
Atom type	σ	ε	q	σ	ε	q
O_{sc}	2.900	0.5858	-0.3602	3.278	0.3538	-0.3602
C_{o1c}	3.550	0.2929	0.6287	2.573	0.0684	0.6287
O_{1c}	2.960	0.8786	-0.5206	2.992	0.5348	-0.5206
C_{a1c}	3.550	0.2929	-0.0530	3.603	0.6300	-0.0530
C_{a2c}	3.550	0.2929	-0.0986	2.586	1.4318	-0.0986
C_{a3c}	3.550	0.2929	-0.1150	3.961	0.1060	-0.1150
C_{a4c}	3.550	0.2929	-0.0931	2.241	2.5582	-0.0931
H_{a2c}	2.420	0.1255	0.1320	2.295	1.1048	0.1320
H_{a3c}	2.420	0.1255	0.1357	2.716	0.0050	0.1357
C_{o2c}	3.550	0.2929	0.7255	4.067	0.0050	0.7255
O_{2c}	2.960	0.8786	-0.5519	3.304	0.1847	-0.5519
C_{1c}	3.500	0.2761	-0.0946	3.254	1.9696	-0.0946
C_{2c}	3.500	0.2761	0.1380	2.000	0.7550	0.1380
H_{1c}	2.500	0.1255	0.1356	2.553	0.3198	0.1356
H_{2c}	2.500	0.1255	0.0799	2.399	0.7344	0.0799
O_{hc}	3.120	0.7113	-0.6865	3.574	0.0949	-0.6865
H_{oc}	0.000	0.0000	0.4213	2.000	0.0050	0.4213

Table S14: Intermolecular parameters for *n***RMU** PET chains: tail unit (C. see main text). σ (Å), ε (kJ/mol) and q (e^-) Tr-FF parameters (OPLS [20, 21]) are reported in the first columns for comparison.

S4 FAMD simulations

Both the OPLS FF, [20] automatically generated with the LigParGen server, [21] and the here parameterized QMD-FFs were employed in lengthy FAMD simulations of polymeric systems made up of a number of 10**RMU**, 20**RMU**, 50**RMU** or 100**RMU** chains. All runs were carried out with the GROMACS engine. [22]

S4.1 Equilibration procedure

To equilibrate each system, we adopted the annealing protocol proposed by Lightfoot et al. [23] as described in the following. Initially, elongated *n***RMU** chains were positioned parallel to each other in a cubic box with periodic boundary conditions (PBCs) in all three directions. The equilibration protocol began with a 10 ns NPT simulation at 100 atm and 750 K to reach realistic densities, followed by a gradual cooling to ambient conditions over 1 ns. Amorphous states were then generated through four annealing cycles, using a temperature gradient from 298 to 1200 to 298 K with a ramp of 0.72 K/ps over 10 ns, and subsequently relaxed by a final 1 ns equilibration under ambient conditions. To overcome kinetic trapping and enhance sampling, 20 independent amorphous states were prepared by assigning different random velocities during the annealing process. All equilibrated systems were eventually used as starting configurations for 100 ns production runs, carried out at ambient conditions as described in the main text.

S4.2 T_g calculation

To compute the T_g the following stepwise procedure was adopted. The systems were equilibrated at 550 K for 10 ns and subsequently a gradient cooling process was conducted within the temperature range of 550 K to 50 K for 100 ns, with a quenching rate of 5 K ns^{-1}. The T_g was determined by linear fitting of the density-temperature relationship in the rubbery and glassy regimes, that is, as the intersection point of the two fitting curves.

S4.3 Additional FAMD results

Figure S7 displays the correlation plots for selected dihedral angles. The comparison highlights distinct conformational sampling between the Tr-FF and QMD-FF force fields: Tr-FF tends to overpopulate certain regions of the dihedral space while under-representing others, reflecting systematic differences in their underlying conformational preferences.

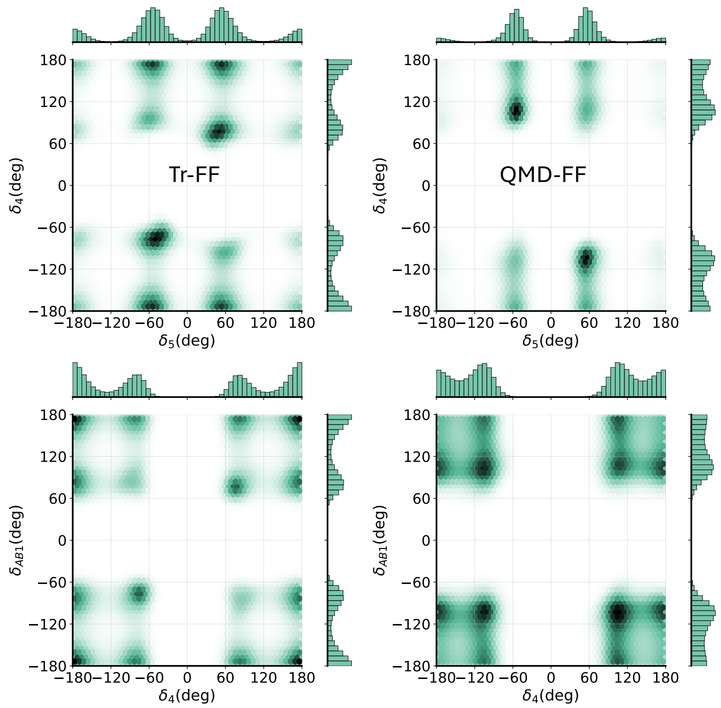

Figure S7: 2D maps highlighting correlation between dihedrals from FA-MD trajectories obtained by Tr-FF (left) and QMD-FF (right). Dihedrals are defined in the main text.

Figure S8 presents two-dimensional distributions of the ring–ring distance as a function of the angular parameters θ and α for both the Tr-FF and QMD-FF force fields. Although Tr-FF reproduces ring–ring angles comparable to those obtained from QMD-FF, it systematically predicts larger inter-ring separations. This deviation originates from differences in the balance between non-bonded dispersion and electrostatic interactions in the two models. In Tr-FF, the weaker dispersion contribution and less accurate representation of π–π stacking interactions reduce the energetic stabilization of closely packed configurations, thereby favoring more expanded conformations. As a result, the Tr-FF force field yields less compact molecular

arrangements and consequently lower bulk densities compared to QMD-FF.

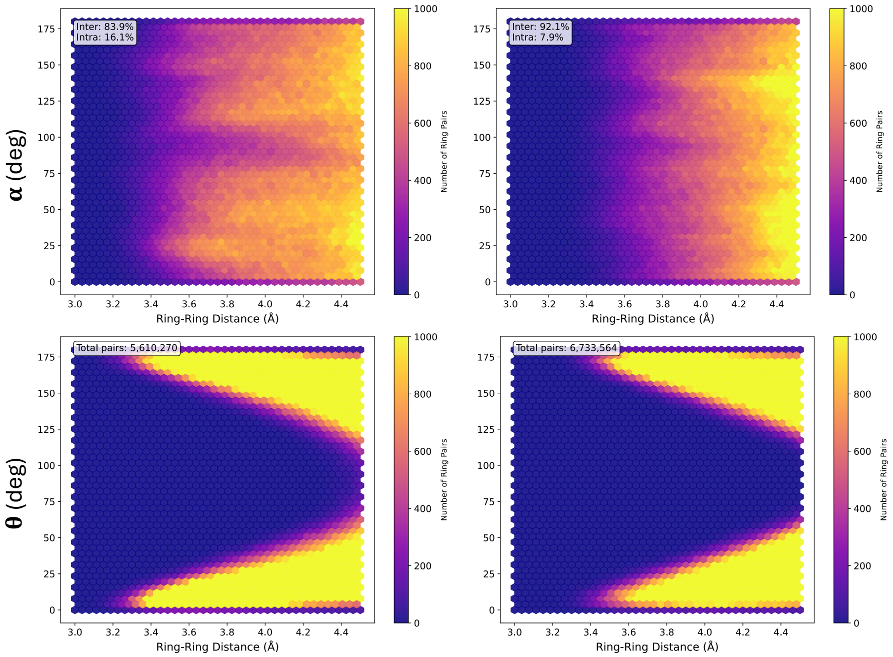

Figure S8: 2D plots of θ (bottom row) and α (top) against the Ring-Ring distance comparing QMD-FF (left column) and Tr-FF (right).

In Figure S9 we compare the R_g of the different PET models. As can be observed, the distribution is shifted to larger R_g values for increasing chain length.

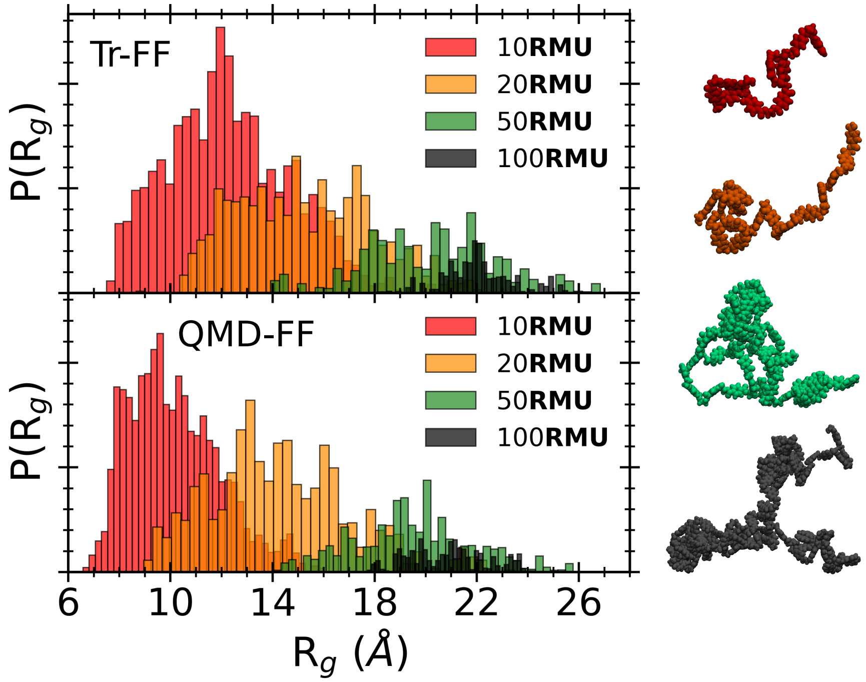

Figure S9: Radius of gyration (R_g) as a function of chain size obtained with Tr-FF and QMD-FF. Snapshots of single chains are also given for each model.

S5 CG-FF parameterization

S5.1 Iterative Boltzmann inversion

The iterative Boltzmann inversion (IBI) method is one of the most widely employed structure-based approaches for developing CG models [24–26]. According to this protocol, the effective pairwise potentials between CG beads in the lower-resolution model are constructed to match the structural distribution functions observed in the high-resolution FA system. The IBI method is based on Henderson's theorem [27] which states that, for a given radial distribution function (RDF), there exists a unique pair interaction potential (up to an additive constant) that produces this RDF. Under the assumption of a lack of correlation among the different degrees of freedom (i.e. the distance between two beads, r, the CG bond length, l, the CG bending angle, θ, and CG dihedral angle, ϕ) the total probability distribution function can be expressed as

$$P_{tot}(q) = \prod P_{bond}(l) \prod P_{angle}(\theta) \prod P_{dihedral}(\phi) \prod P_{NB}(r) \tag{S12}$$

where $P_{bond}(l)$, $P_{angle}(\theta)$ and $P_{dihedral}(\phi)$ are the bond length, angular, and dihedral probability distributions, respectively. $P_{NB}(r)$ is the non-bonding term, which is usually retrieved from the radial distribution function $g(r)$. Similarly, an effective potential can be extracted for each term independently, exploiting the Boltzmann inverse of the reference distributions:

$$\begin{aligned} U_{bond}(l) &= -k_B T \sum \ln\left(P_{bond}(l)/l^2\right), \\ U_{angle}(\theta) &= -k_B T \sum \ln\left(P_{angle}(\theta)/\sin\theta\right), \\ U_{dihedral}(\phi) &= -k_B T \sum \ln P_{dihedral}(\phi), \\ U_{NB}(r) &= -k_B T \sum \ln g(r) \end{aligned} \tag{S13}$$

where k_B is the Boltzmann constant and T is the absolute temperature, and the bond length and angular distribution functions are normalized with their respective volume elements. The total effective potential is then given by

$$U_{tot}(r,l,\theta,\phi) = U_{bond}(l) + U_{angle}(\theta) + U_{dihedral}(\phi) + U_{NB}(r). \tag{S14}$$

Generally, direct Boltzmann inversion does not match the target distribution functions. This

means that the CG potentials have to be modified in an iterative procedure through

$$U_{i+1}^{CG}(\chi) = U_i^{CG}(\chi) + k_B T \ln \left[\frac{P_i^{CG}(\chi)}{P^{ref}(\chi)} \right] \tag{S15}$$

where i is the iteration number and $P_{ref}^{CG}(\chi)$ is the reference atomistic distribution function. This iterative process is continued until the target distribution is reproduced to a sufficient degree, which can be measured with a target function

$$f^i = \frac{1}{r_{max}} \int_0^{r_{max}} (g^{ref}(r) - g^i(r))^2 dr \tag{S16}$$

where r_{max} is the cut-off distance of the CG RDF. Even though convergence is achieved with the target RDFs, the IBI-derived CG-FF cannot accurately reproduce the pressure of the atomistic reference model. Therefore, to resolve this issue, a linear correction term is added to the long-range part of the CG non-bonded potentials to match the true pressure [26, 28]:

$$U_{corrected}^{CG}(r) = U_{original}^{CG}(r) - \alpha \left(1 - \frac{r}{r_{max}} \right) \tag{S17}$$

where α is a suitable constant.

In the initial step, carried out using MagiC's built-in scripts, 10 independent high resolution FA-MD trajectories were concatenated and mapped into a CG one, based on the selected CG mapping. The mass, charge and position of each bead were assigned as the total mass, charge and center of mass of its atomic constituents. In a second stage, the reference bond length and angle distribution functions (equation (S12) as well as the radial distribution functions were computed based on the mapped trajectory. A cutoff of 25 Å was used for the latter. All possible combinations between CG beads, including edges, were considered to describe intermolecular short-range pairwise interactions.

The final step concerns with the solution of the inverse problem, *i.e.*, obtaining the effective CG potentials from the reference distribution functions. This was performed by the MagiC's kernel module which implements IBI, starting with the reference distribution functions and then refining iteratively the potentials (see equation (S15)). At each iteration, a classical NVT Metropolis Monte Carlo (MC) simulation was used to explore the configurational space, hence avoiding a new CGMD simulation at each iteration. During MC runs, three types of moves

were allowed: random atom displacements, molecule translations and molecule rotations. After each iteration, the distributions computed from the MC step were confronted with the reference ones, and appropriate corrections were applied to obtain a new set of potentials. This process was repeated until a given threshold on the objective function (equation S16) was satisfied. In our case, we performed 50 iterations of IBI, with each iteration including a MC simulation of 3×10^7 equilibration steps and 1×10^8 sampling steps, until the deviation between the CG and reference distributions was below 2%.

S5.2 Postprocessing for CGMD simulations

To postprocess the CG-FF for subsequent CGMD simulations, the obtained tabulated intramolecular effective potentials were fitted to the following functional forms. The bond stretching and angle bending potentials were fitted to a multicentered Gaussian-based potential (gaussian bond/angle style in LAMMPS [29]):

$$U_{bond}(l) = -k_B T \ln\left[\sum_{i=1}^{n} \frac{A_i}{\omega_i\sqrt{\pi/2}} \exp\left(\frac{-2(l-l_i)^2}{\omega_i^2}\right)\right], \quad \text{(S18)}$$

$$U_{angle}(\theta) = -k_B T \ln\left[\sum_{i=1}^{n} \frac{A_i}{\omega_i\sqrt{\pi/2}} \exp\left(\frac{-2(\theta-\theta_i)^2}{\omega_i^2}\right)\right]. \quad \text{(S18)}$$

The number of Gaussian terms depended on the complexity of the potentials, oscillating between a single term for the purely harmonic springs to six for the softer potentials. Hence, this functional form is able to describe the different equilibrium states. The torsion angles were instead fitted to a 5-term cosine Fourier series (multi/harmonic dihedral style in LAMMPS):

$$U_{dihedral}(\phi) = \sum_{i=1}^{5} A_i \cos^{i-1}\phi. \quad \text{(S19)}$$

However, for mapping scheme C we had to add two extra Fourier coefficients to the A−C−C−A dihedral due to its more complex shape, changing the dihedral style to anharmonic. Due to the intrinsic difficulty to extract the LJ parameters from the RDFs, we decided to maintain a tabular form for the intermolecular potentials. Short-range potentials were extrapolated with a repulsive wall in the core region using MagiC's built-in tools and further interpolated with Gaussian smoothing to remove kinks.

S6 CGMD simulations

S6.1 Convergence analysis of CG models

The deviations between the CG and FA models in non-bonded RDFs are calculated with the merit function of equation (S16). In Figure S10 we show the convergence of the total RDF during the IBI procedure. The deviation f^i shows a rapid decay in the first 10 cycles. This is followed by an oscillatory behaviour for the next 30 cycles, finally stabilizing below the acceptable error range.

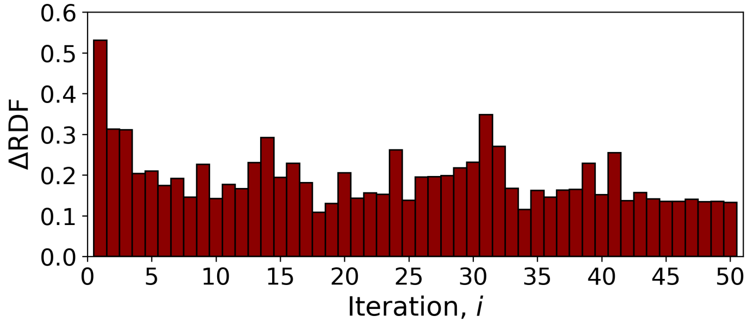

Figure S10: Total deviation between the RDFs obtained in the IBI process after each iteration and the reference RDFs corresponding to mapping scheme A.

To gain further insight, in Figure S11 we deconvolute the deviation of all individual pairwise interactions. All bonded potentials are able to converge within 50 iterations of Boltzmann inversion. The deviation in ABA and BAC angles decays very fast while the others present significant oscillations. It becomes apparent that increasing the number of iterations will not improve convergence. The RDFs involving inner CG beads and only one edge bead also show a relatively fair convergence. However, E_1E_1, E_1E_2 and E_2E_2 fall above the error threshold of 2%.

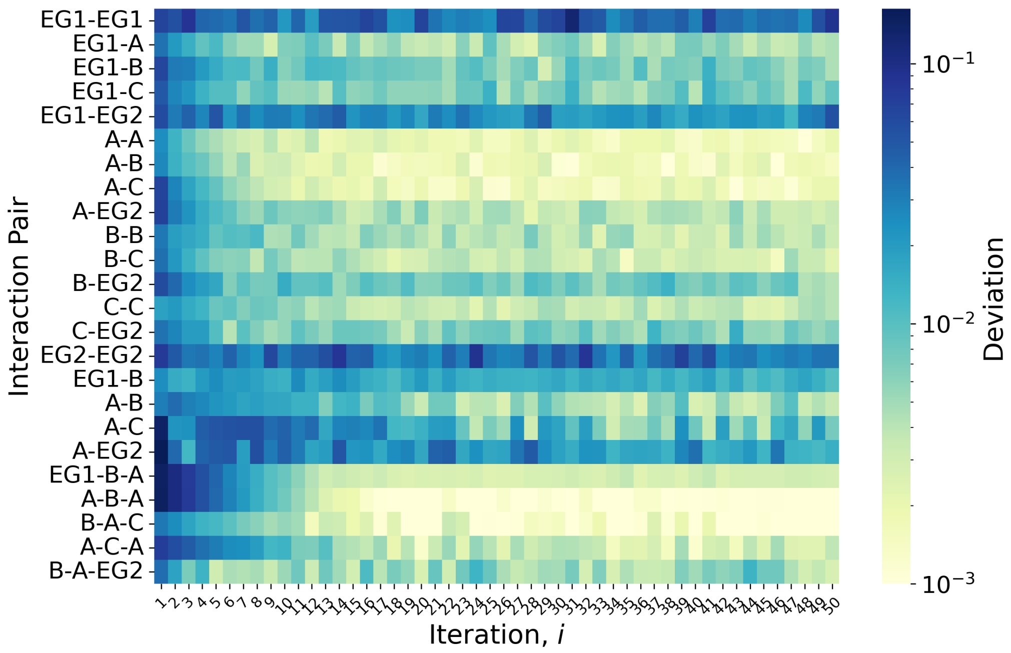

Figure S11: Total deviation between the RDFs, bond stretching and angle bending distribution functions obtained in the IBI process after each iteration and the reference distributions corresponding to scheme mapping A. The distribution functions at each iteration shown herein include raw data without any postprocessing to eliminate noise, hence after postprocessing (see section S5.2) the final distribution functions show a much better agreement.

S6.2 Structural properties comparison of CG models

In the following, we make a detailed comparison of the local structural properties obtained with the different CG mapping schemes, applied either along the Tr-FF or QMD-FF based trajectories. All bonded and non-bonded distributions are provided in Figures S12-S41.

S6.2.1 Bond distributions

In all mapping schemes the E_1X (X = B, B_1, A) and AB and AB_1 bond pairs present Gaussian-type distributions. Their relative position changes because the bead size changes from one mapping to another. It is interesting to note that in scheme C, $P_{bond}(l_{AC})$ becomes localized

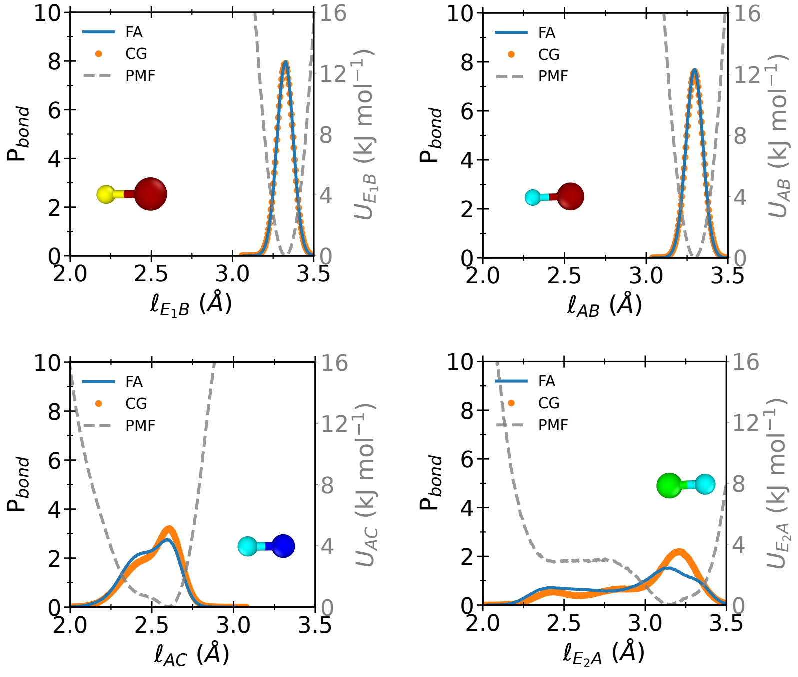

Figure S12: Averaged probability distribution functions for bond stretching of the 10**RMU** system obtained using mapping scheme A on QMD-FF at FA (blue lines) and CG level (orange circles). In all panels, the corresponding CG effective potentials are displayed with grey dashed lines.

because the information on δ_{OCCO} dihedral is lost. However, now we have gained access to the C$-C$ part of δ_{OCCO} through the $P_{bond}(l_{CC})$. Two well-separated peaks are observed at $l = 2.1$ Å and $l = 2.4$ Å corresponding to the *gauche* and *trans* states. As a result of partitioning the

phenol ring into a three-membered cycle in scheme B, a new B_1B_2 bond pair appears with very stiff peaks. The bond distribution of E_2A is very similar in scheme A and B, whereas it becomes

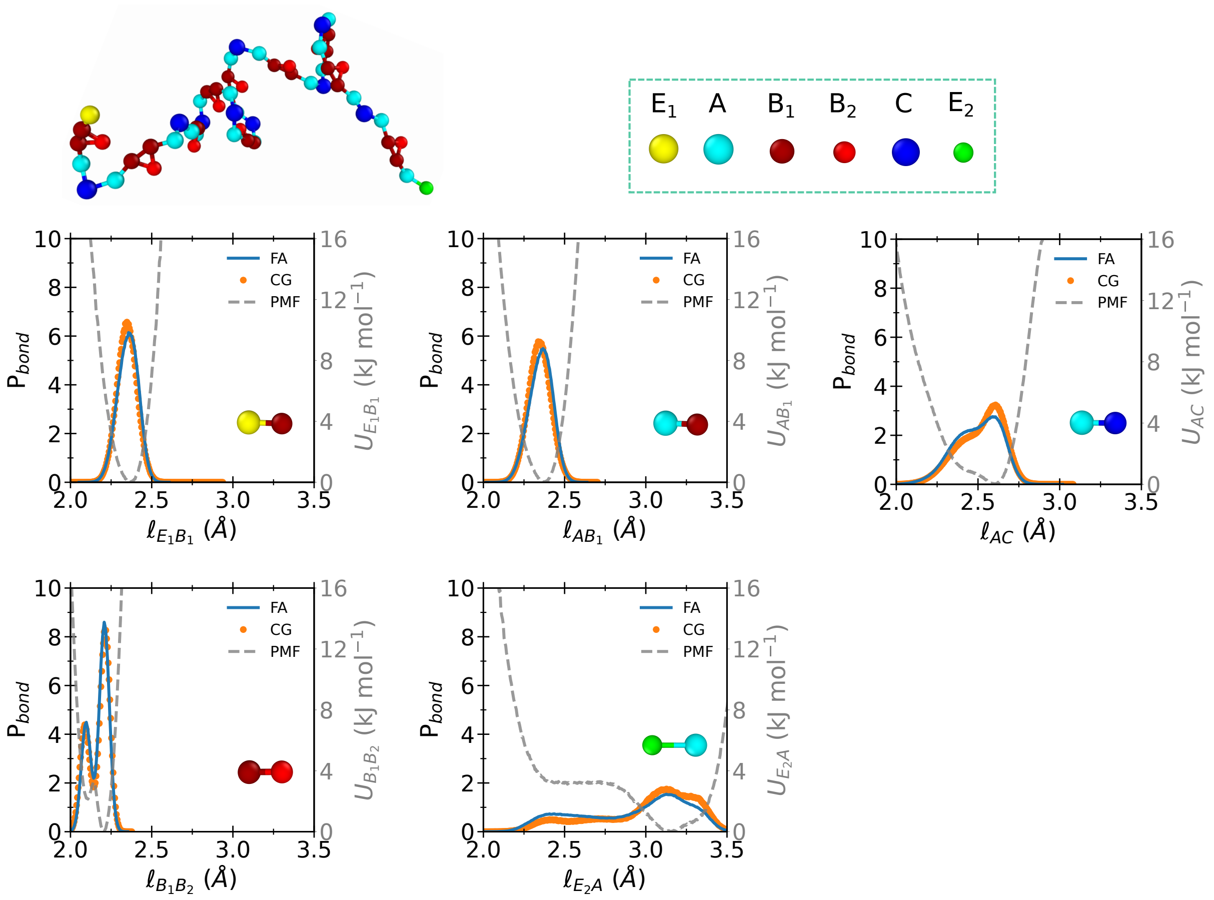

Figure S13: Averaged probability distribution functions for bond stretching of the 10**RMU** system obtained using mapping scheme B on QMD-FF at FA (blue lines) and CG level (orange circles). In all panels, the corresponding CG effective potentials are displayed with grey dashed lines.

more localized in scheme C. The reason is that now bead A is a second neighbor and thus, the structural fingerprints of the hydrogen bonding between the terminal OH and carbonyl are no longer captured by this bond distribution.

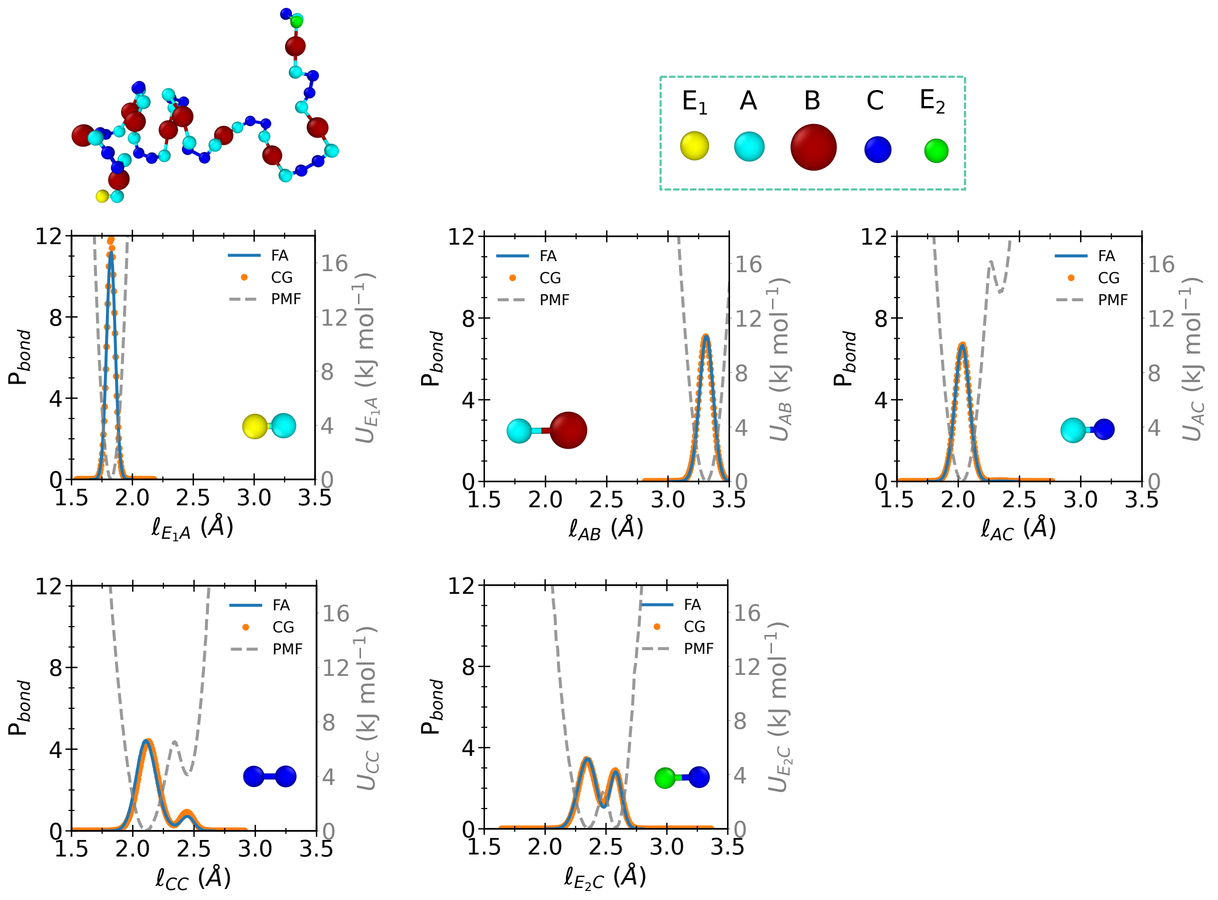

Figure S14: Averaged probability distribution functions for bond stretching of the 10**RMU** system obtained using mapping scheme C on QMD-FF at FA (blue lines) and CG level (orange circles). In all panels, the corresponding CG effective potentials are displayed with grey dashed lines.

Comparing the CG-QMD force fields with CG-Tr, we can see that the shoulder in $P_{bond}(l_{AC})$ is splitted to another peak in scheme B, similar to scheme A. The same behavior is observed for $P_{bond}(l_{E_2A})$. CG-Tr-B also shifts the E_1B band to larger l, while B_1B_2 remains mainly unchanged. Instead, for mapping C both CG-FFs yield very similar distribution functions, the main difference being a shift in $P_{bond}(l_{AB})$ and $P_{bond}(l_{E_2C})$, as well as a population change in $P_{bond}(l_{CC})$.

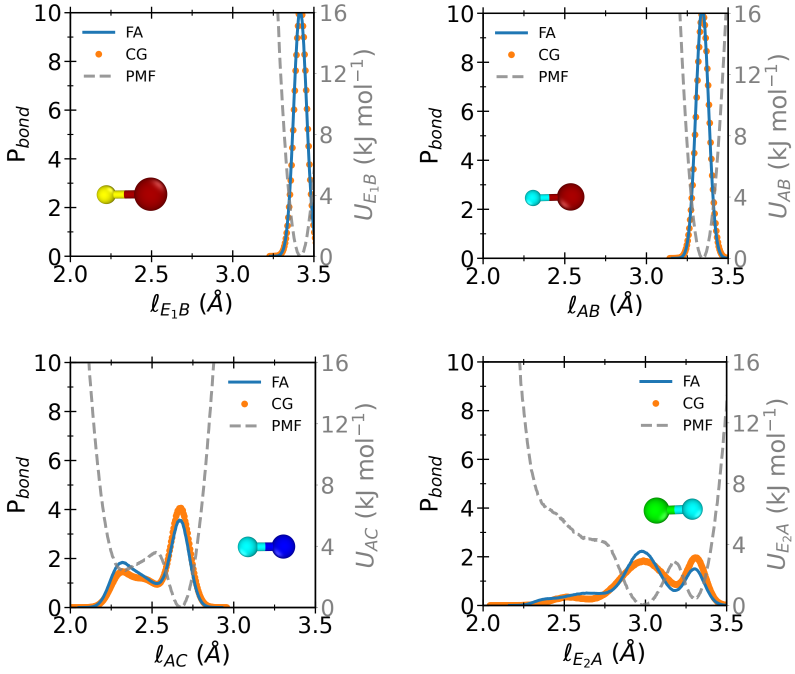

Figure S15: Averaged probability distribution functions for bond stretching of the 10**RMU** system obtained using mapping scheme A on Tr-FF at FA (blue lines) and CG level (orange circles). In all panels, the corresponding CG effective potentials are displayed with grey dashed lines.

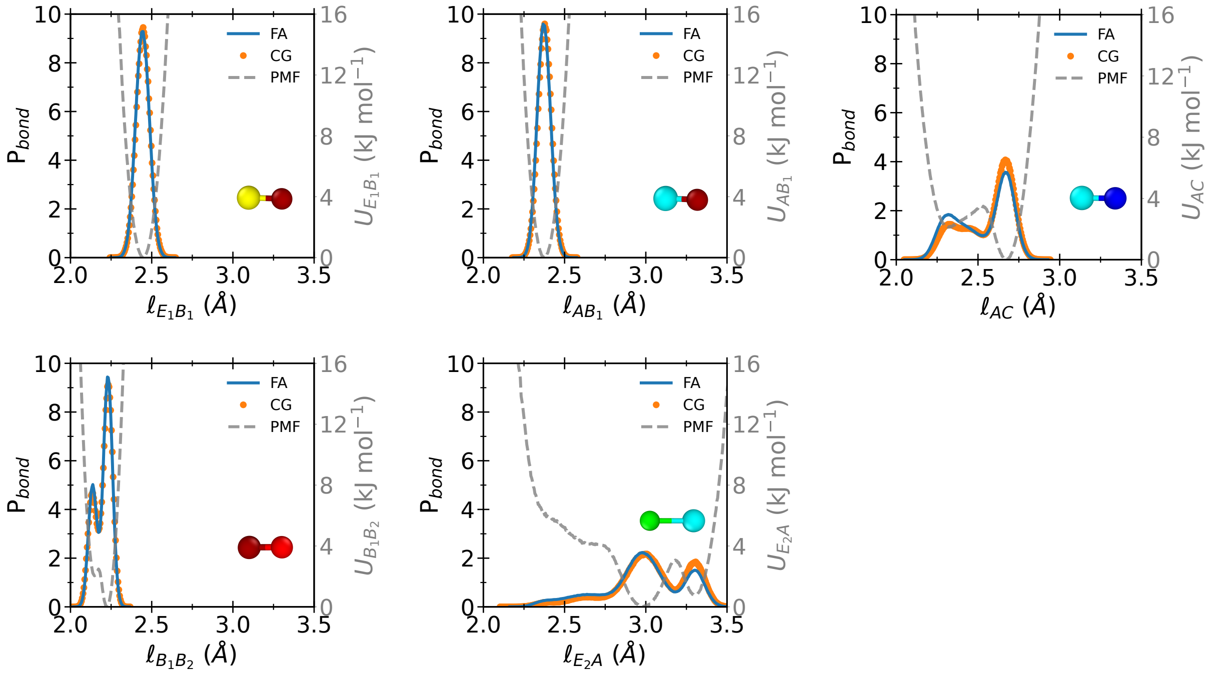

Figure S16: Averaged probability distribution functions for bond stretching of the 10**RMU** system obtained using mapping scheme B on Tr-FF at FA (blue lines) and CG level (orange circles). In all panels, the corresponding CG effective potentials are displayed with grey dashed lines.

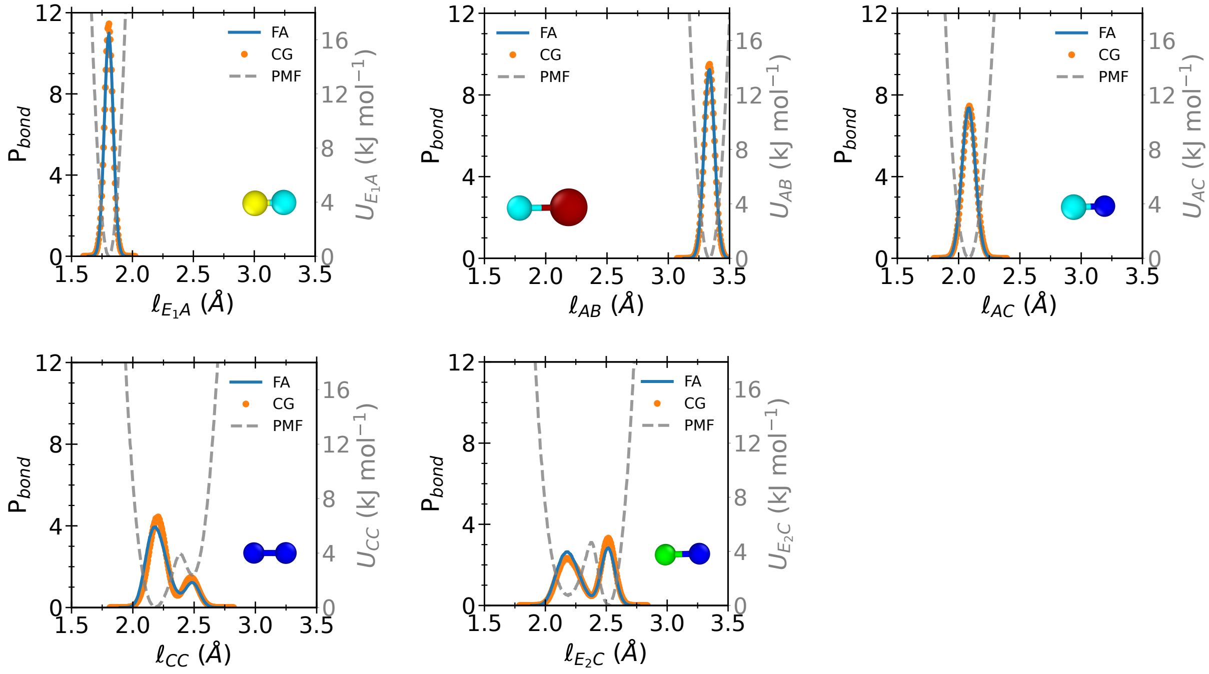

Figure S17: Averaged probability distribution functions for bond stretching of the 10**RMU** system obtained using mapping scheme C on Tr-FF at FA (blue lines) and CG level (orange circles). In all panels, the corresponding CG effective potentials are displayed with grey dashed lines.

S6.2.2 Angle distributions

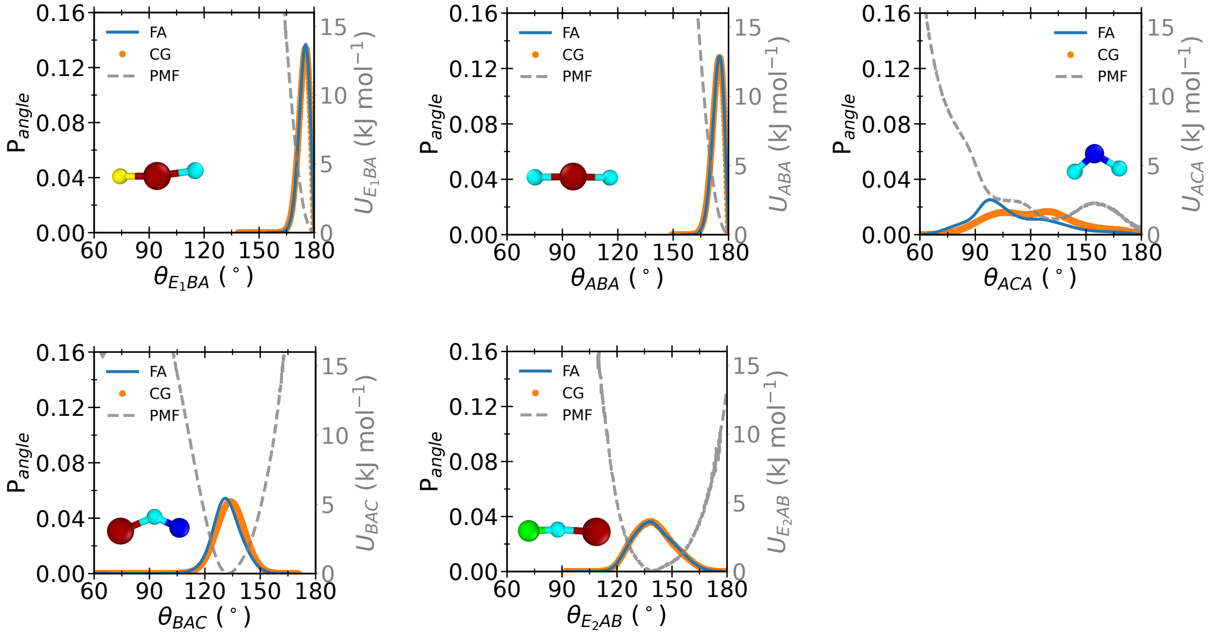

Figure S18: Averaged probability distribution functions for angle bending of the 10**RMU** system obtained using mapping scheme A on QMD-FF at FA (blue lines) and CG level (orange circles). In all panels, the corresponding CG effective potentials are displayed with grey dashed lines.

The angle distribution functions in scheme B are found to be either very stiff or very spread. Such differences are caused by the stiffness of the aromatic beads. A similar pattern is observed for scheme C too, but somewhat less pronounced. $P_{angle}(\theta_{B_1AC})$ broadens in scheme B compared to the corresponding distribution in mapping A, showing two maxima. $P_{angle}(\theta_{E_2AB_1})$ closely follows the same trend, while the opposite occurs in Scheme C. The ester group is partitioned into CG beads A and B, making the BAC angle stiffer. ACC angle shows an extremely soft distribution.

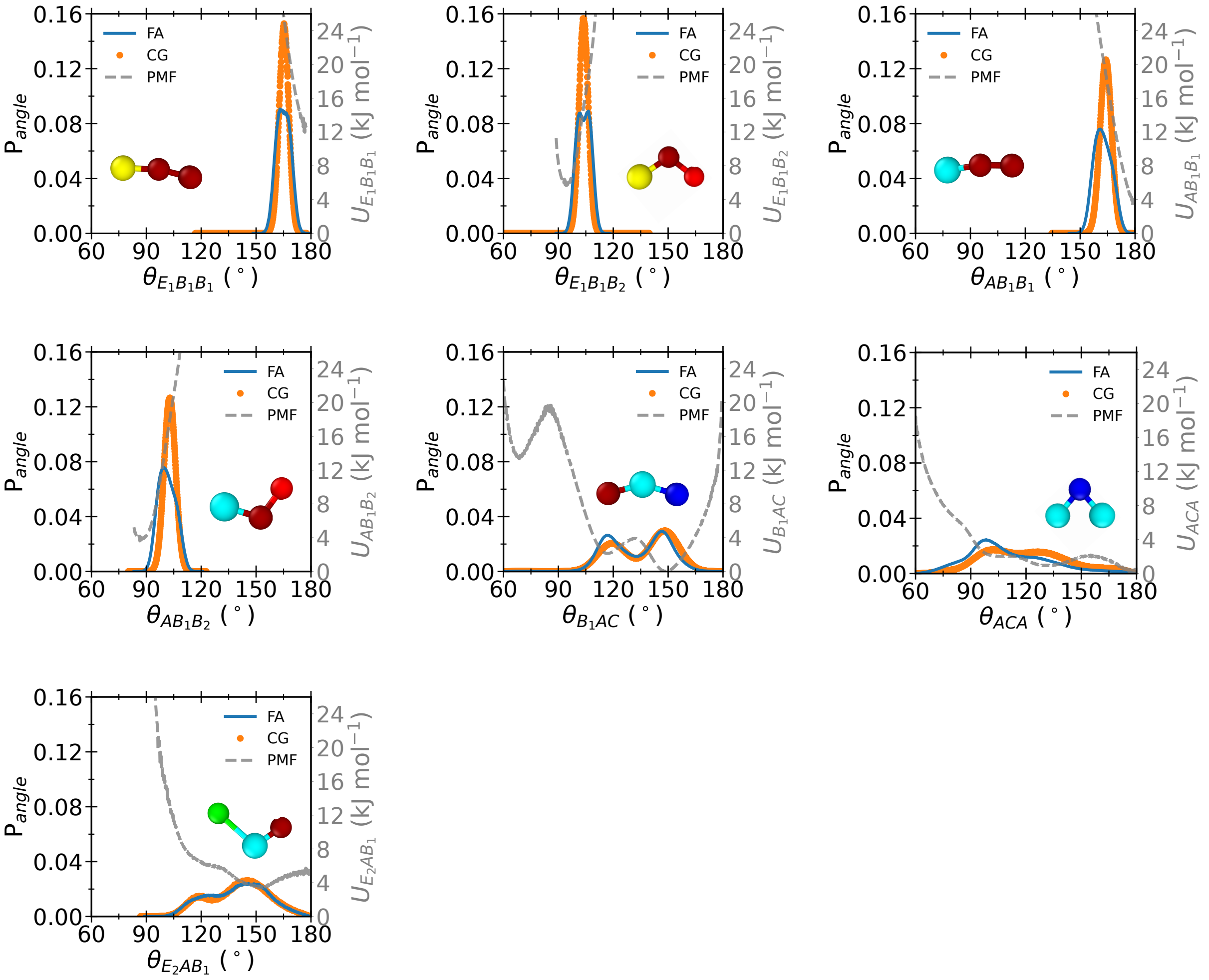

Figure S19: Averaged probability distribution functions for angle bending of the 10**RMU** system obtained using mapping scheme B on QMD-FF at FA (blue lines) and CG level (orange circles). In all panels, the corresponding CG effective potentials are displayed with grey dashed lines.

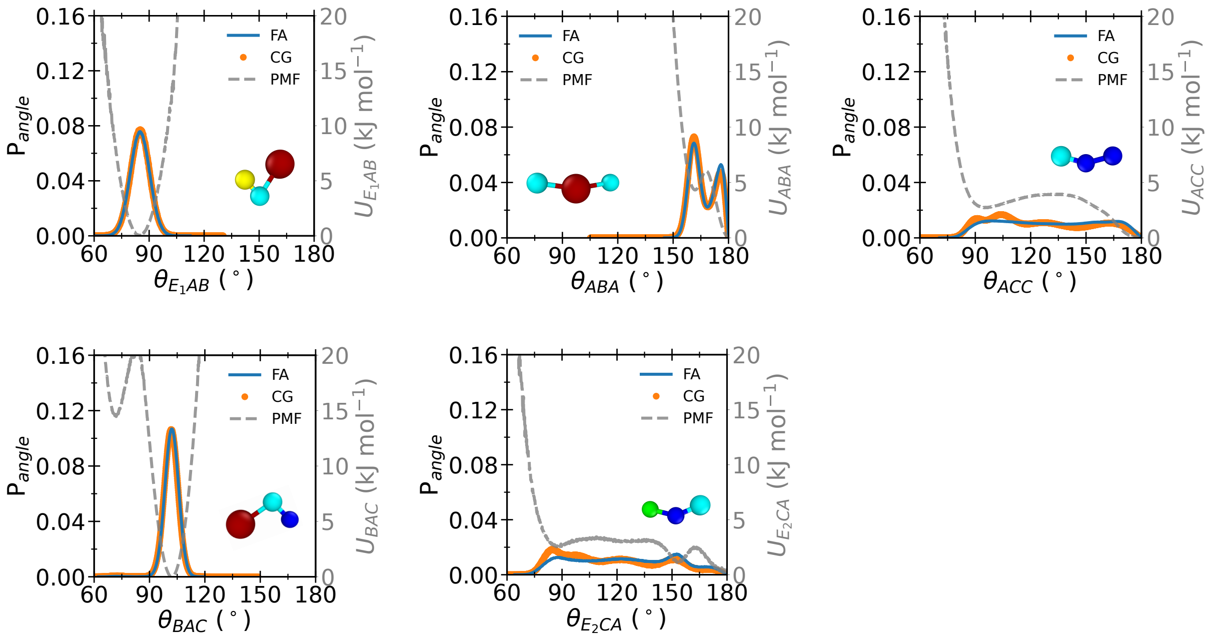

Figure S20: Averaged probability distribution functions for angle bending of the 10**RMU** system obtained using mapping scheme C on QMD-FF at FA (blue lines) and CG level (orange circles). In all panels, the corresponding CG effective potentials are displayed with grey dashed lines.

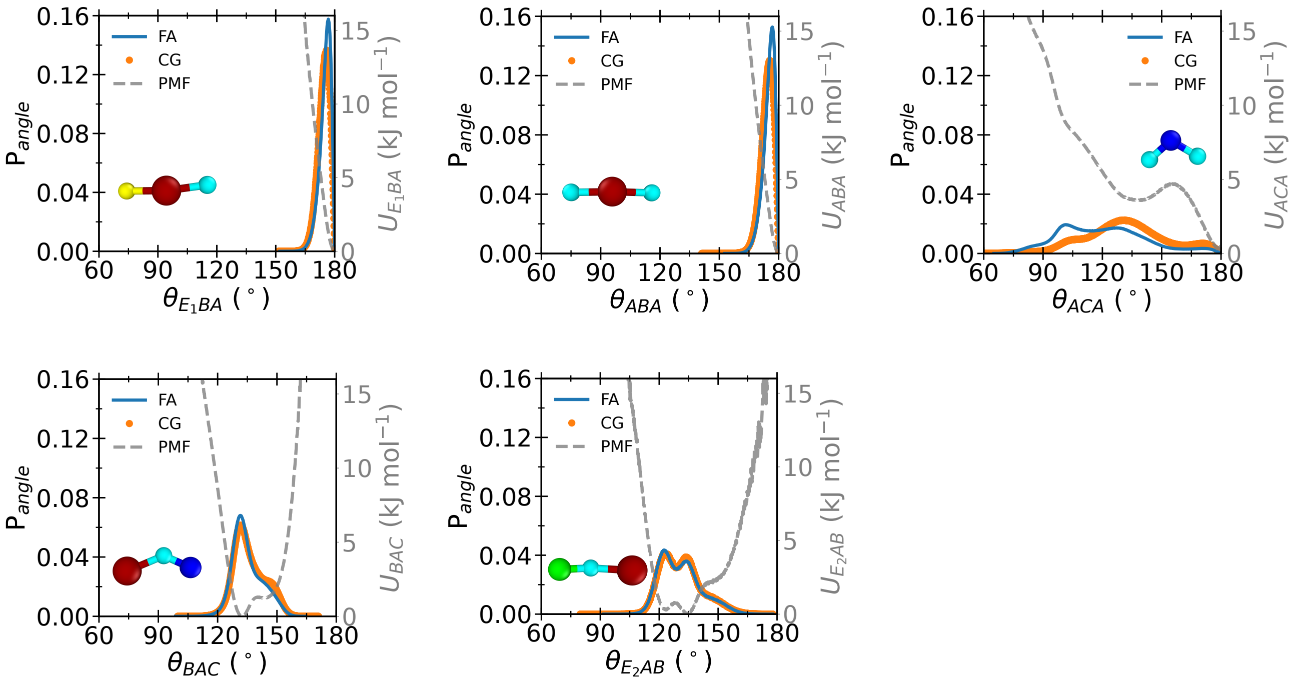

Figure S21: Averaged probability distribution functions for angle bending of the 10**RMU** system obtained using mapping scheme A on Tr-FF at FA (blue lines) and CG level (orange circles). In all panels, the corresponding CG effective potentials are displayed with grey dashed lines.

The populations computed with CG-Tr-B are very similar to those of CG-QMD-B. The distributions involving two aromatic beads become stiffer with the former CG-FF. Similarly, CG-Tr-C gives sharper peaks for E_1AB and BAC angles. ACC and E_2CA continue being very smooth but now show two distinct peaks.

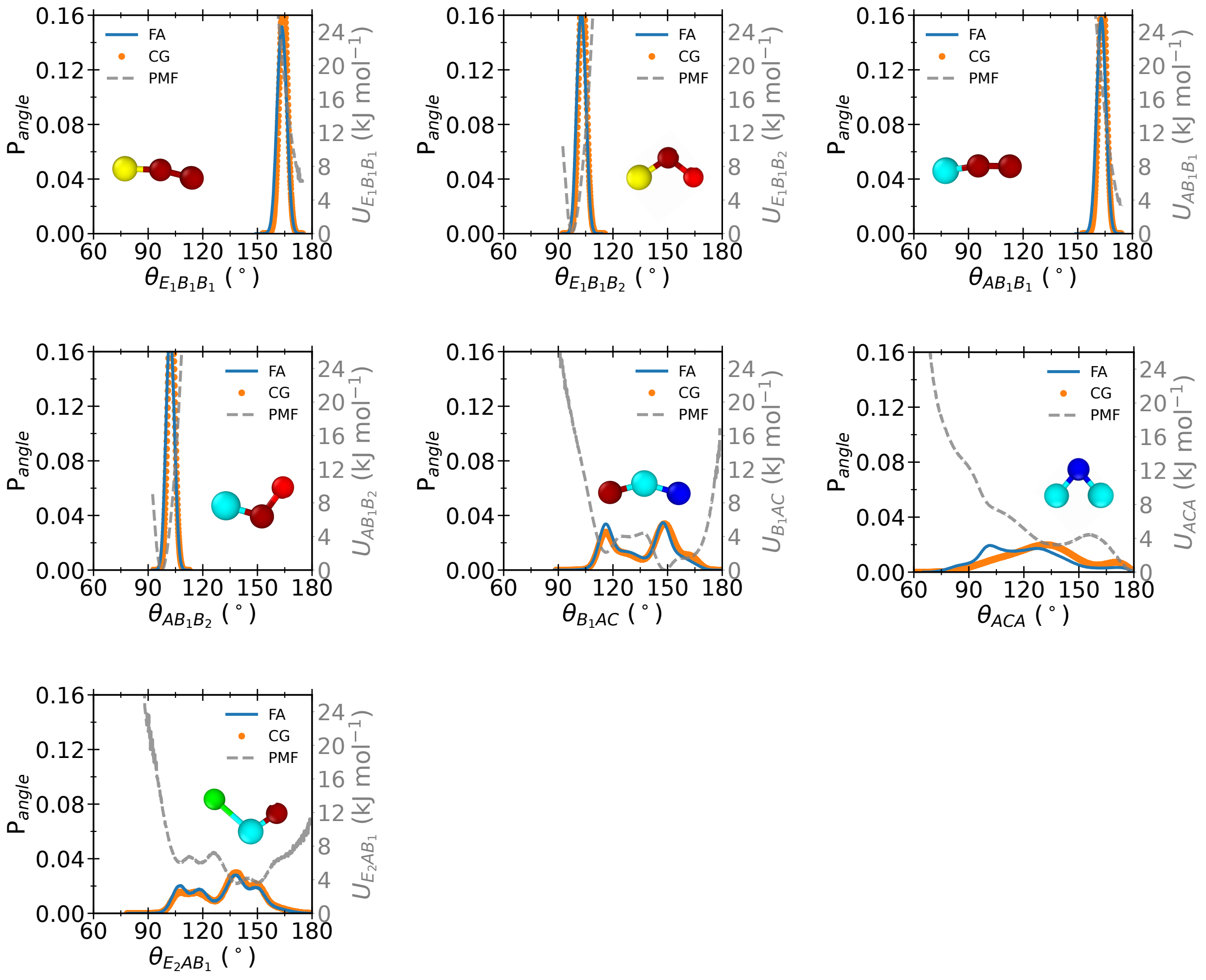

Figure S22: Averaged probability distribution functions for angle bending of the 10**RMU** system obtained using mapping scheme B on Tr-FF at FA (blue lines) and CG level (orange circles). In all panels, the corresponding CG effective potentials are displayed with grey dashed lines.

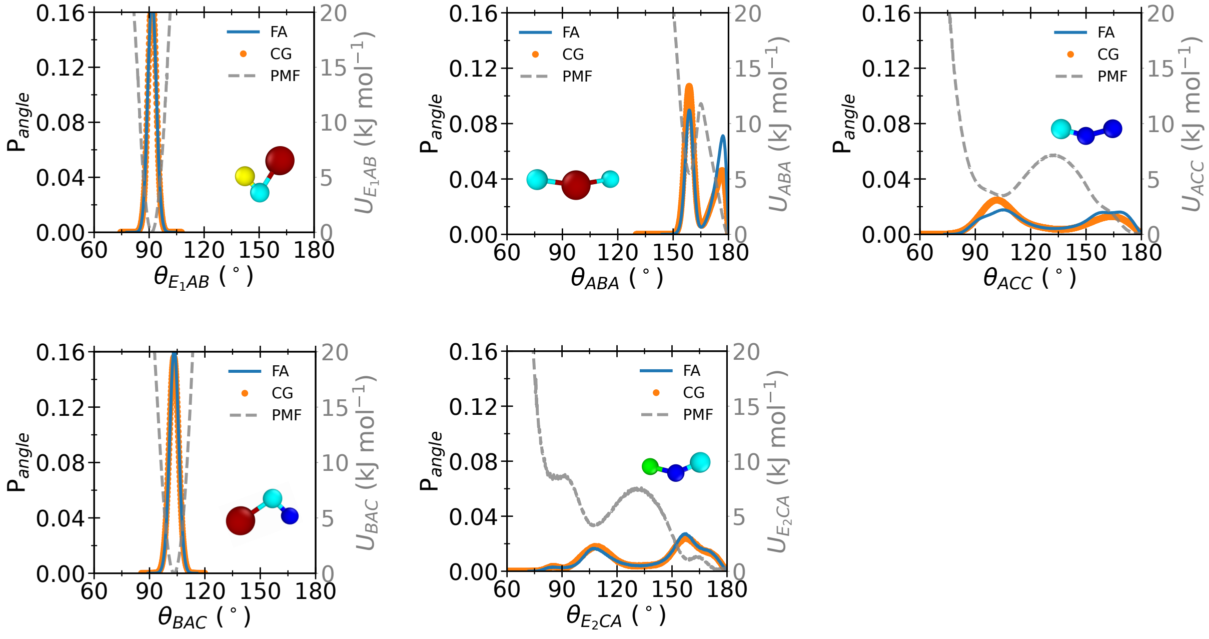

Figure S23: Averaged probability distribution functions for angle bending of the 10**RMU** system obtained using mapping scheme C on Tr-FF at FA (blue lines) and CG level (orange circles). In all panels, the corresponding CG effective potentials are displayed with grey dashed lines.

S6.2.3 Dihedral distributions

The number of dihedral distribution functions increases to 5 and 9 in scheme C and B, respectively. In scheme B, rotations around the B_1-B_1 and B_1-B_2 bonds are constraint, which translate into very large barriers. Rotations around $A-C$ and $A-B_1$ bonds show comparable

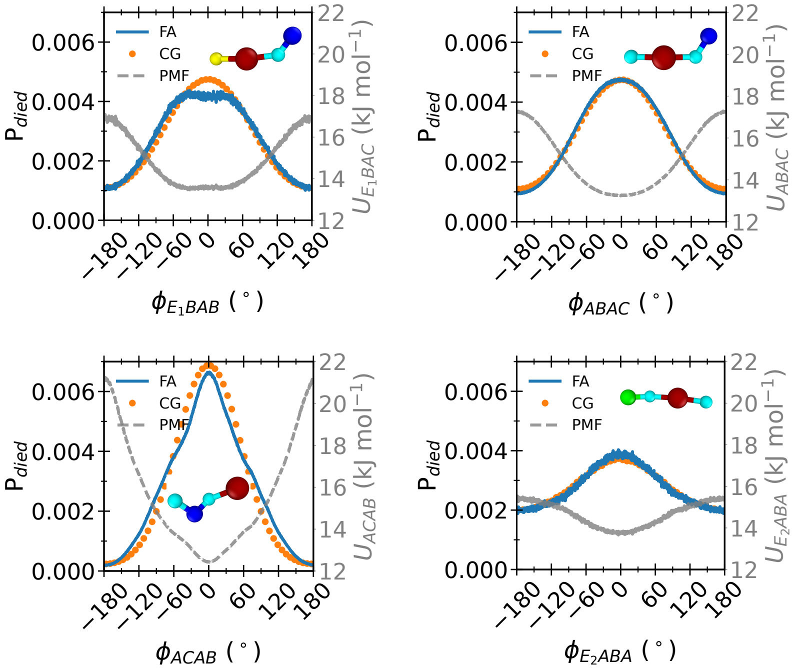

Figure S24: Averaged probability distribution functions for torsion angle of the 10**RMU** system obtained using mapping scheme A on QMD-FF at FA (blue lines) and CG level (orange circles). In all panels, the corresponding CG effective potentials are displayed with grey dashed lines.

barriers to scheme A. However, $\phi_{B_1B_1AC}$ and $\phi_{B_2B_1AC}$ also populate the trans state at -180° and 180° . CG-Tr-B gives very similar curves, with slight changes in the population at 0° and 180° for some dihedrals. The stiffness caused by the bending and rotation around the three-membered ring decreases significantly the speed-up in the CG-MD runs. The largest time step for the bonded interactions becomes no larger than 2 fs.

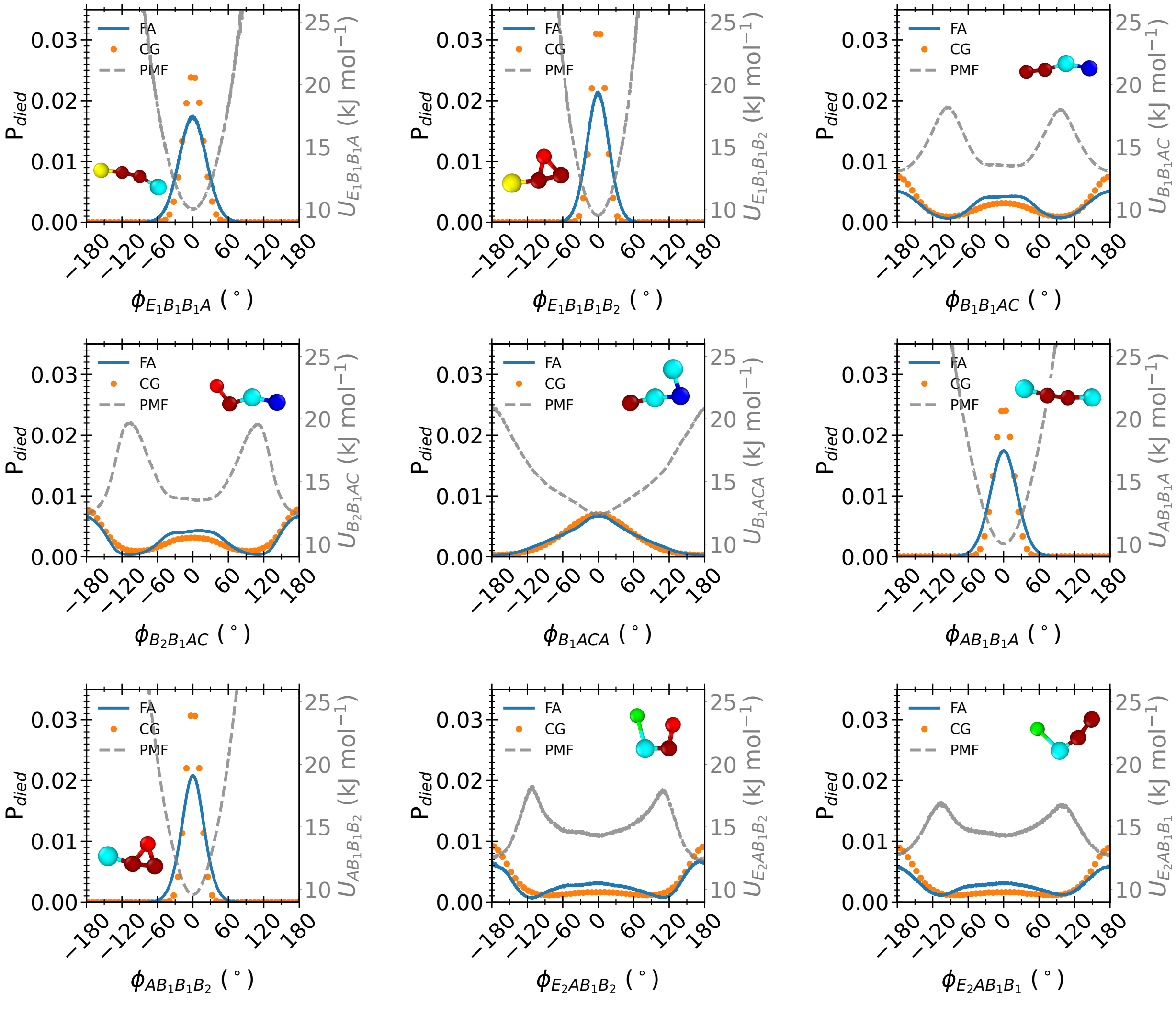

Figure S25: Averaged probability distribution functions for torsion angle of the 10**RMU** system obtained using mapping scheme B on QMD-FF at FA (blue lines) and CG level (orange circles). In all panels, the corresponding CG effective potentials are displayed with grey dashed lines.

The rotation angle energy landscape becomes completely different in scheme C. The peaks

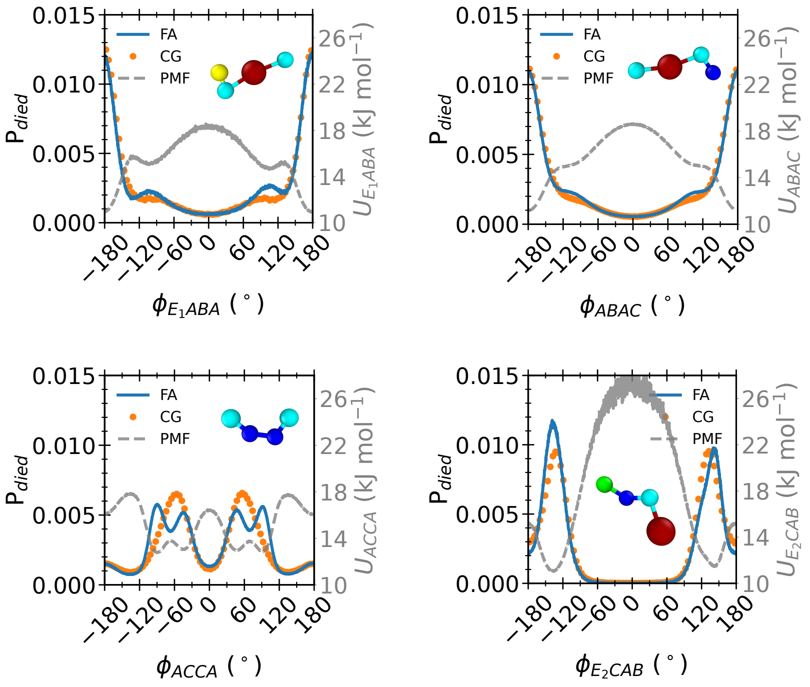

Figure S26: Averaged probability distribution functions for torsion angle of the 10**RMU** system obtained using mapping scheme C on QMD-FF at FA (blue lines) and CG level (orange circles). In all panels, the corresponding CG effective potentials are displayed with grey dashed lines.

at 0° disappear and instead most of the dihedrals show two symmetric peaks at 100° (BACC), 140° (E_2CAB) and 180° (ABAC, E_1ABA) of considerable height. As expected, rotations around the ester group (BACC) is stiff, and also around A−B is much larger than in scheme A. ϕ_{ACCA}, "equivalent" of the atomistic δ_{OCCO}, shows a complex double-peak structure at 45° and 90° corresponding to the *gauche* state, as well as a peak at 180° for the *trans* conformation. The former is splitted because of the A−C free rotation in this mapping scheme.

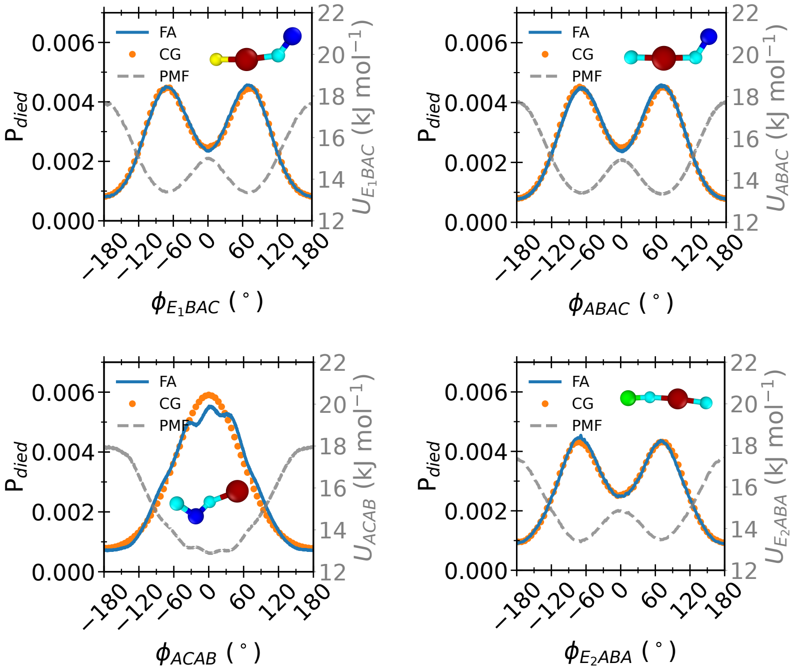

Figure S27: Averaged probability distribution functions for torsion angle of the 10**RMU** system obtained using mapping scheme A on Tr-FF at FA (blue lines) and CG level (orange circles). In all panels, the corresponding CG effective potentials are displayed with grey dashed lines.

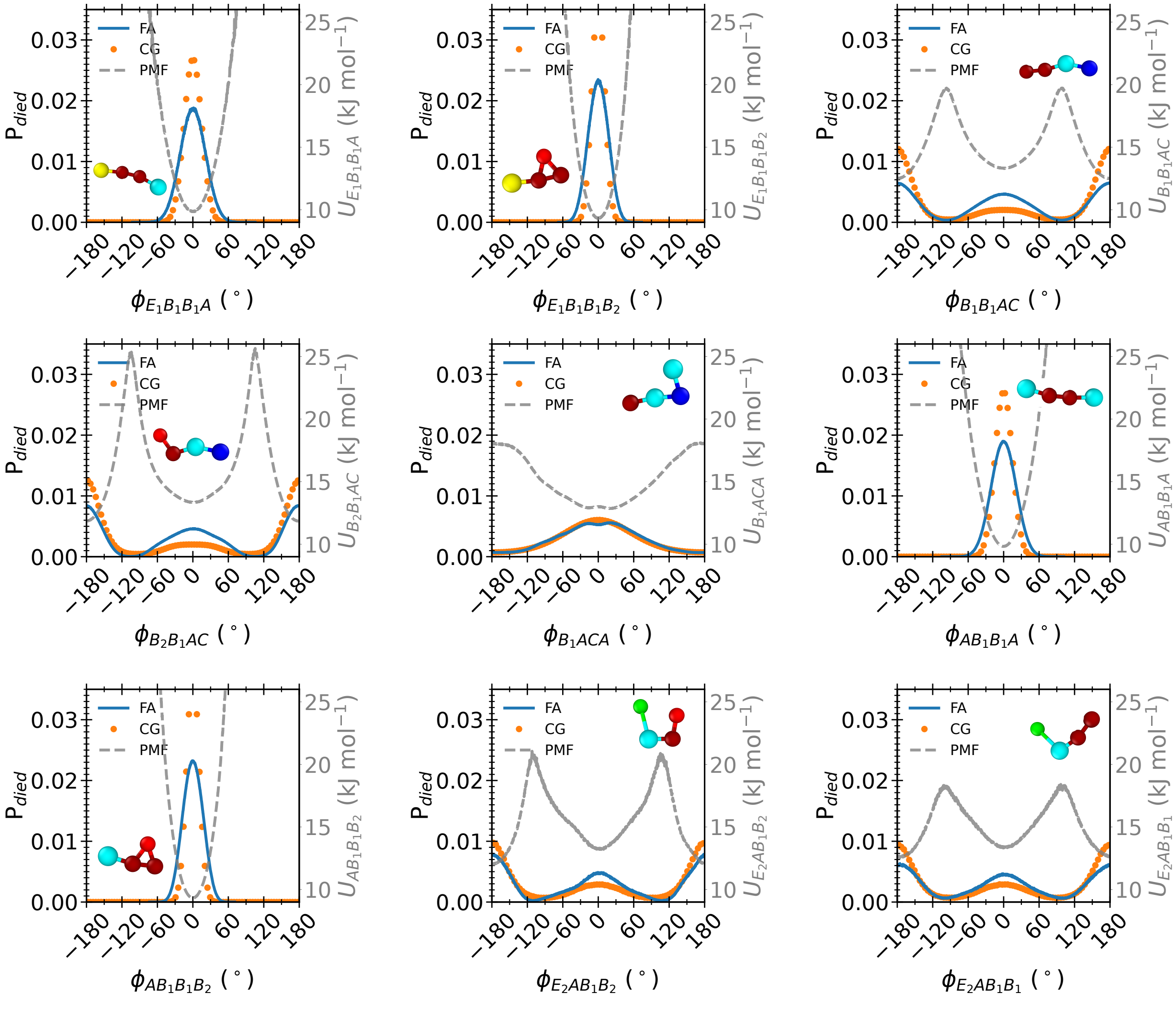

Figure S28: Averaged probability distribution functions for torsion angle of the 10**RMU** system obtained using mapping scheme B on Tr-FF at FA (blue lines) and CG level (orange circles). In all panels, the corresponding CG effective potentials are displayed with grey dashed lines.

Interestingly, CG-OPLS-C softens significantly ϕ_{BACC}, ϕ_{ACCA} and ϕ_{E_2CAB}. This last one now shows two peaks close to each other. The shoulders in ϕ_{E_1ABA} and ϕ_{ABAC} observed with CG-QMD-C now become well-defined peaks. Also the *trans* population in ϕ_{ACCA} increases slightly, in line with the atomistic simulations.

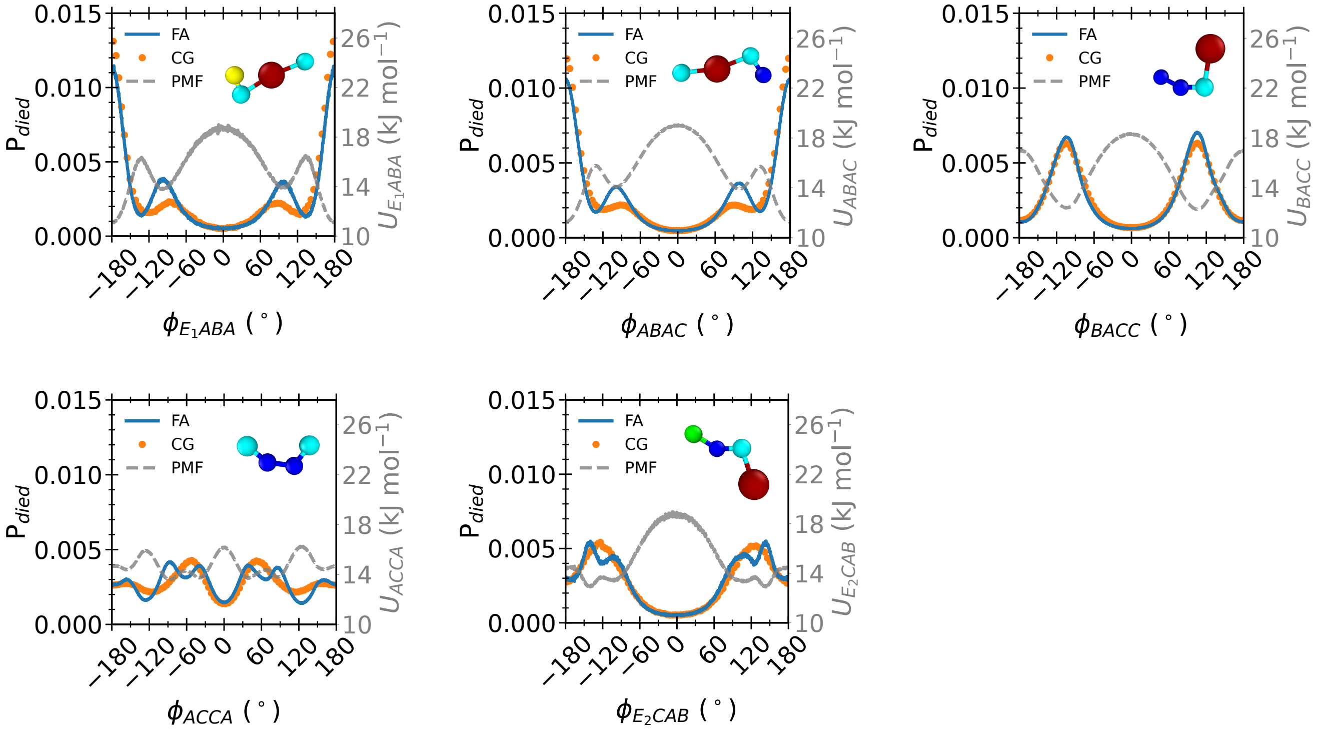

Figure S29: Averaged probability distribution functions for torsion angle of the 10**RMU** system obtained using mapping scheme C on Tr-FF at FA (blue lines) and CG level (orange circles). In all panels, the corresponding CG effective potentials are displayed with grey dashed lines.

S6.2.4 Radial distribution functions

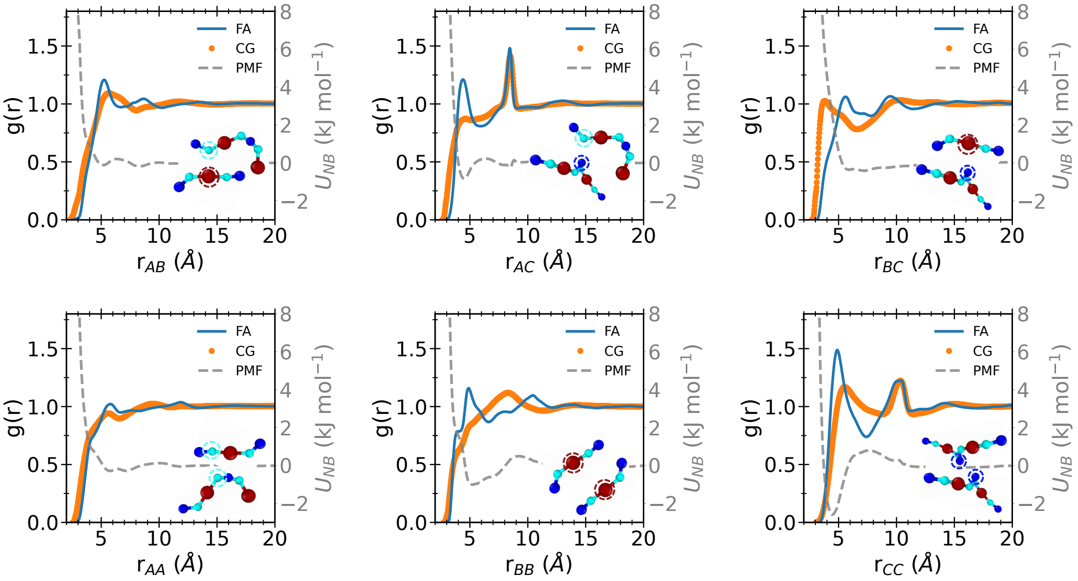

Figure S30: Averaged probability distribution functions for radial distribution functions of the 10**RMU** system obtained using mapping scheme A on QMD-FF at FA (blue lines) and CG level (orange circles). In all panels, the corresponding CG effective potentials are displayed with grey dashed lines. Only distributions involving inner CG beads are included.

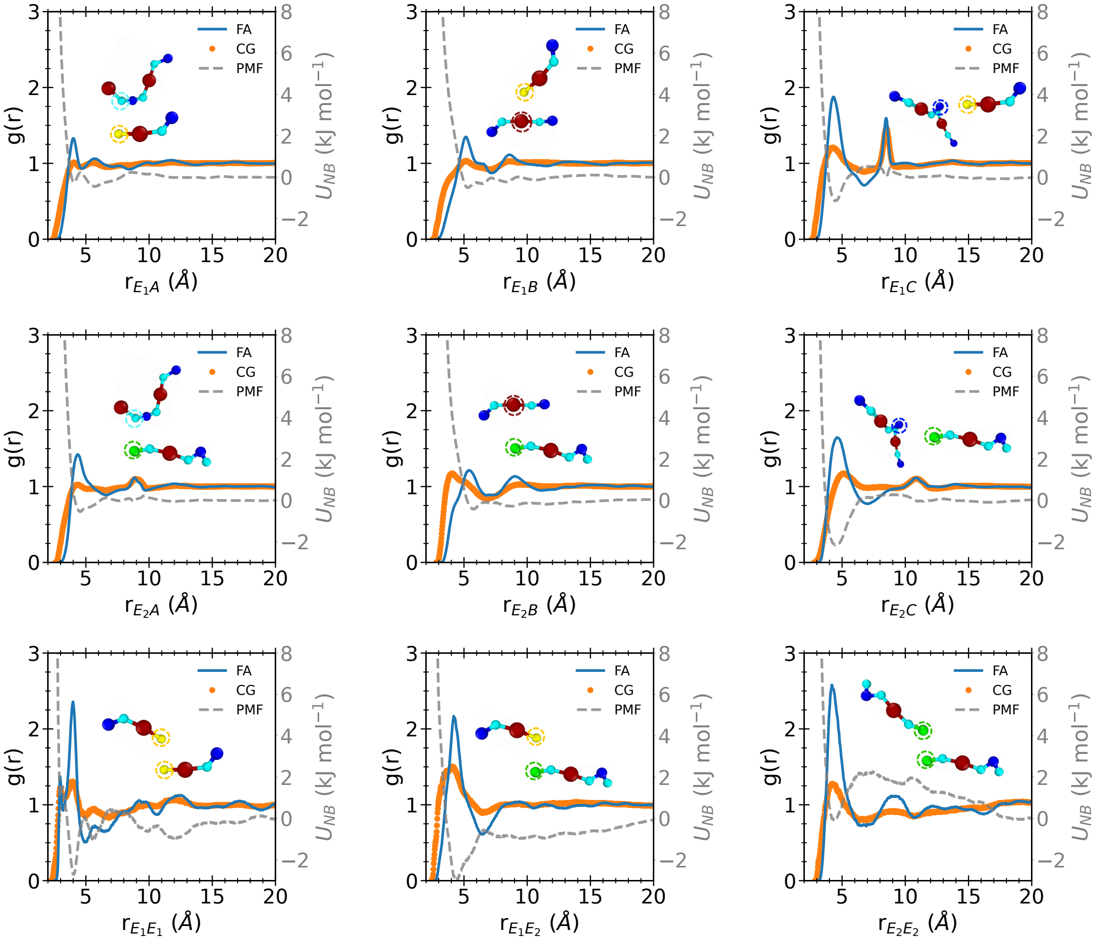

Figure S31: Averaged probability distribution functions for radial distribution functions of the 10**RMU** system obtained using mapping scheme A on QMD-FF at FA (blue lines) and CG level (orange circles). In all panels, the corresponding CG effective potentials are displayed with grey dashed lines. Only distributions involving terminal CG beads are included.

Some of the RDFs are affected by the partitioning of the aromatic ring in scheme B. The

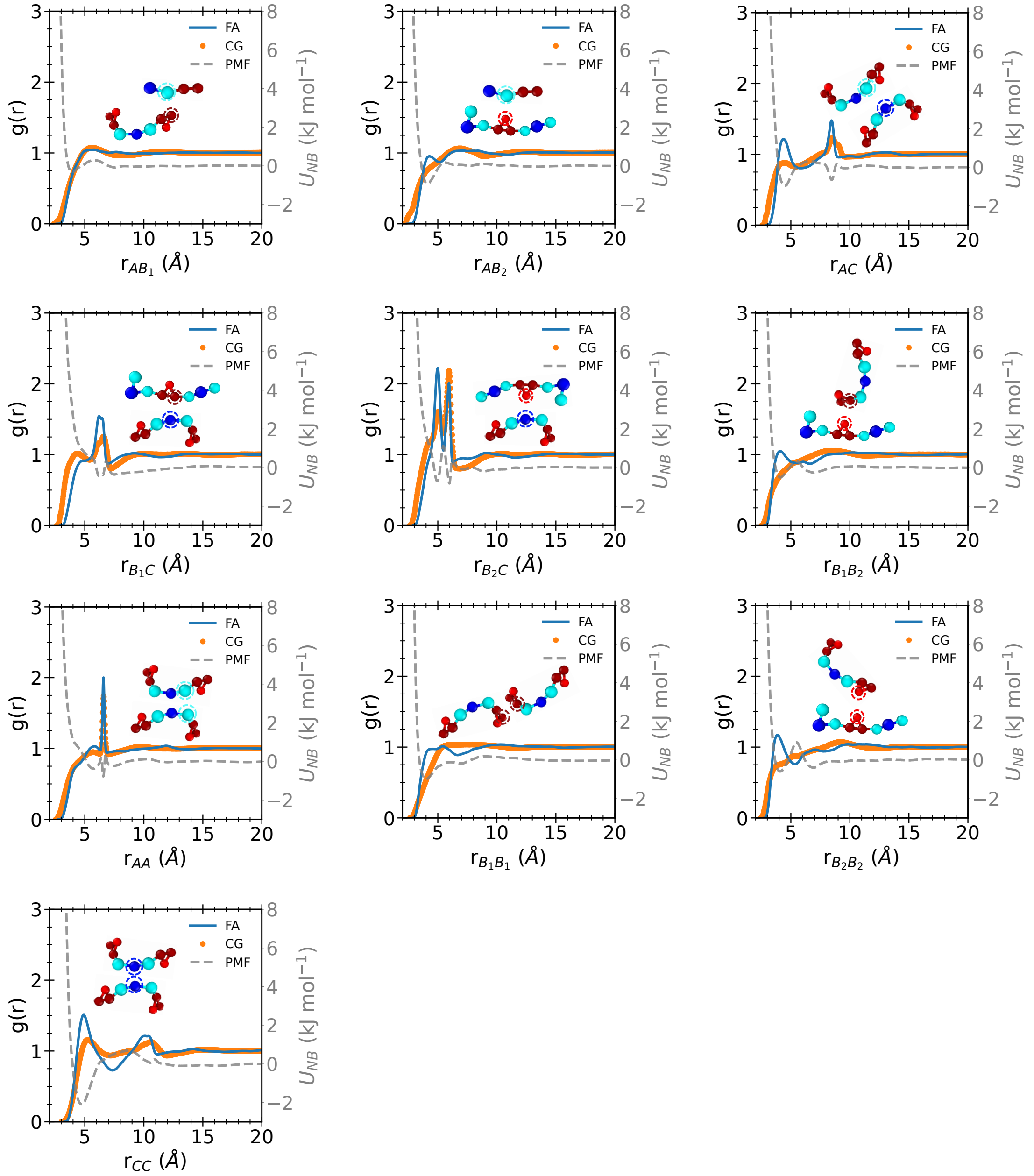

Figure S32: Averaged probability distribution functions for radial distribution functions of the 10**RMU** system obtained using mapping scheme B on QMD-FF at FA (blue lines) and CG level (orange circles). In all panels, the corresponding CG effective potentials are displayed with grey dashed lines. Only distributions involving inner CG beads are included.

most visible change is the appearance of a very sharp peak at 6.5 Å in $g_{AA}(r)$. $g_{AC}(r)$ and $g_{CC}(r)$ remain unchanged with respect to scheme A.

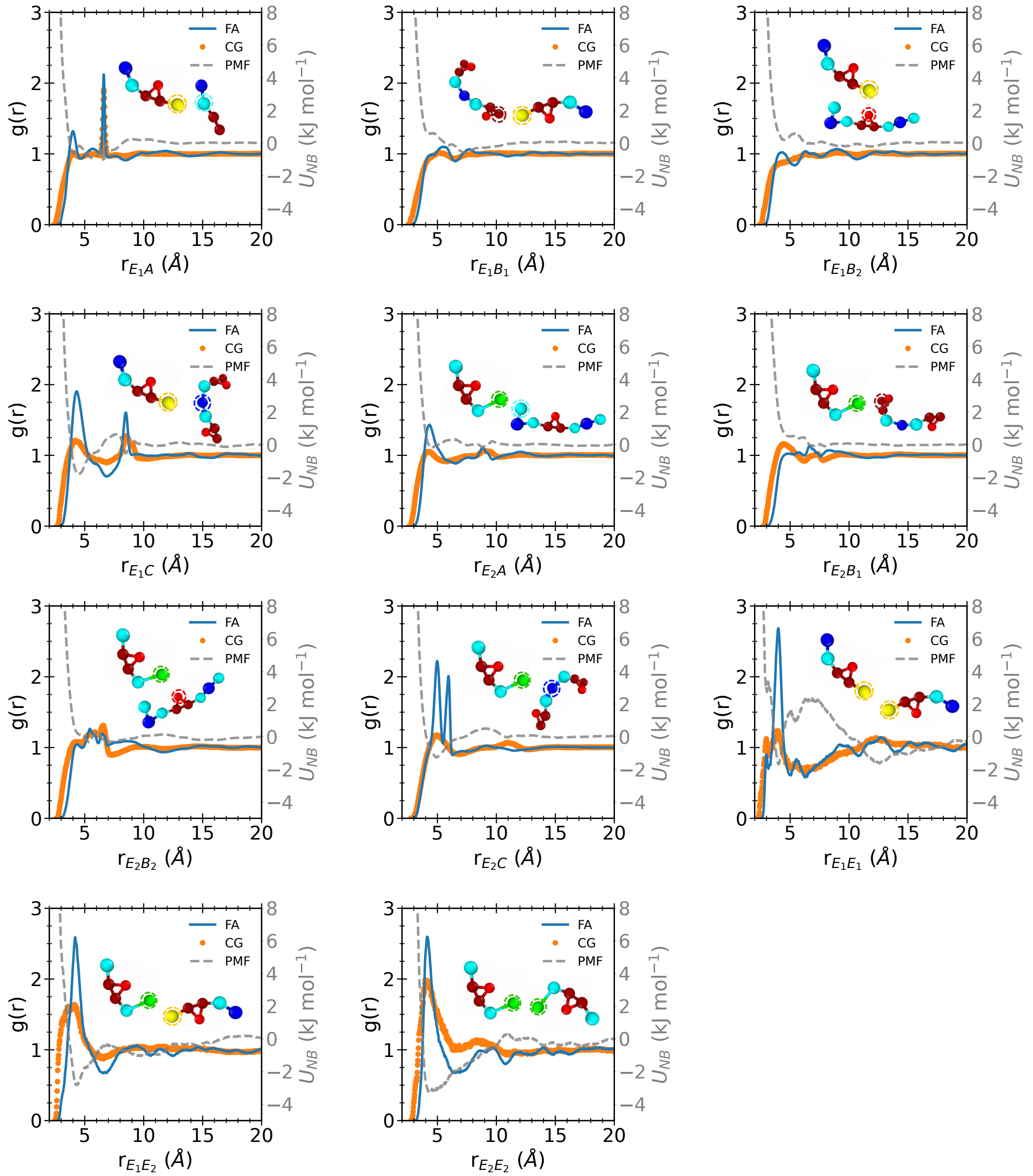

Figure S33: Averaged probability distribution functions for radial distribution functions of the 10**RMU** system obtained using mapping scheme B on QMD-FF at FA (blue lines) and CG level (orange circles). In all panels, the corresponding CG effective potentials are displayed with grey dashed lines. Only distributions involving terminal CG beads are included.

$g_{B_1C}(r)$ and $g_{B_2C}(r)$ show high peaks, while $g_{B_1A}(r)$ and $g_{B_2A}(r)$ show a very smooth profile, suggesting weaker interactions. $g_{B_1C}(r)$ shows a prominent shoulder at 4.8 Å before the first peak at 6.2 Å. The former might reflect the interaction with the closest B_1 bead in the ring,

whereas the latter with the B_1 bead further apart. The intra-ring RDFs differ significantly from the BB RDF of scheme A, both around the first peak and at larger distances, which might reflect the stacking with B_i beads of other chains and beyond 1−4 particle pairs. In CG-Tr-B the $g_{B_1C}(r)$ and $g_{B_2C}(r)$ show a stronger interaction than in CG-QMD-B.

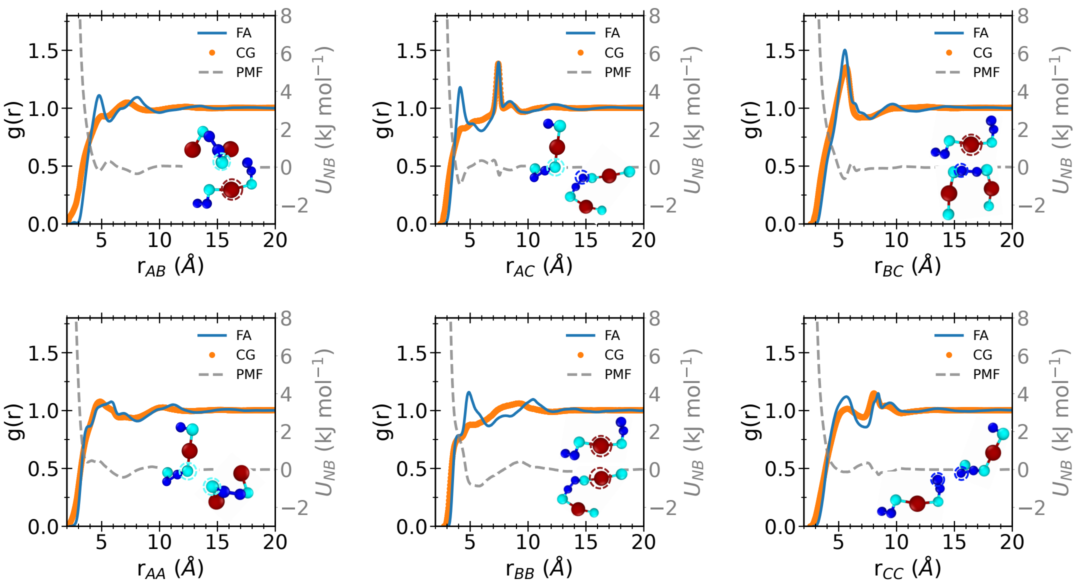

Figure S34: Averaged probability distribution functions for radial distribution functions of the 10**RMU** system obtained using mapping scheme C on QMD-FF at FA (blue lines) and CG level (orange circles). In all panels, the corresponding CG effective potentials are displayed with grey dashed lines. Only distributions involving inner CG beads are included.

$g_{AB}(r)$ has the same profile beyond the second peak in scheme A and C. However, in the latter the first peak has less intensity and is shifted to lower distances due to the smaller bead size. The second peak is also shifted to lower r but presents a higher peak and a shoulder. In $g_{AC}(r)$ the first two peaks are divided into two since now there are two C beads per RMU. The first peak in $g_{BC}(r)$ becomes stronger in scheme C, but after this it overlaps with that of scheme A. CC interaction is significantly weaker in scheme C, whereas the first peaks in AA become somewhat more pronounced. No noticeable differences are found in $g_{BB}(r)$. CG-Tr-C shows similar RDF profiles to CG-QMD-C, but the first peaks are slightly shifted to larger r.

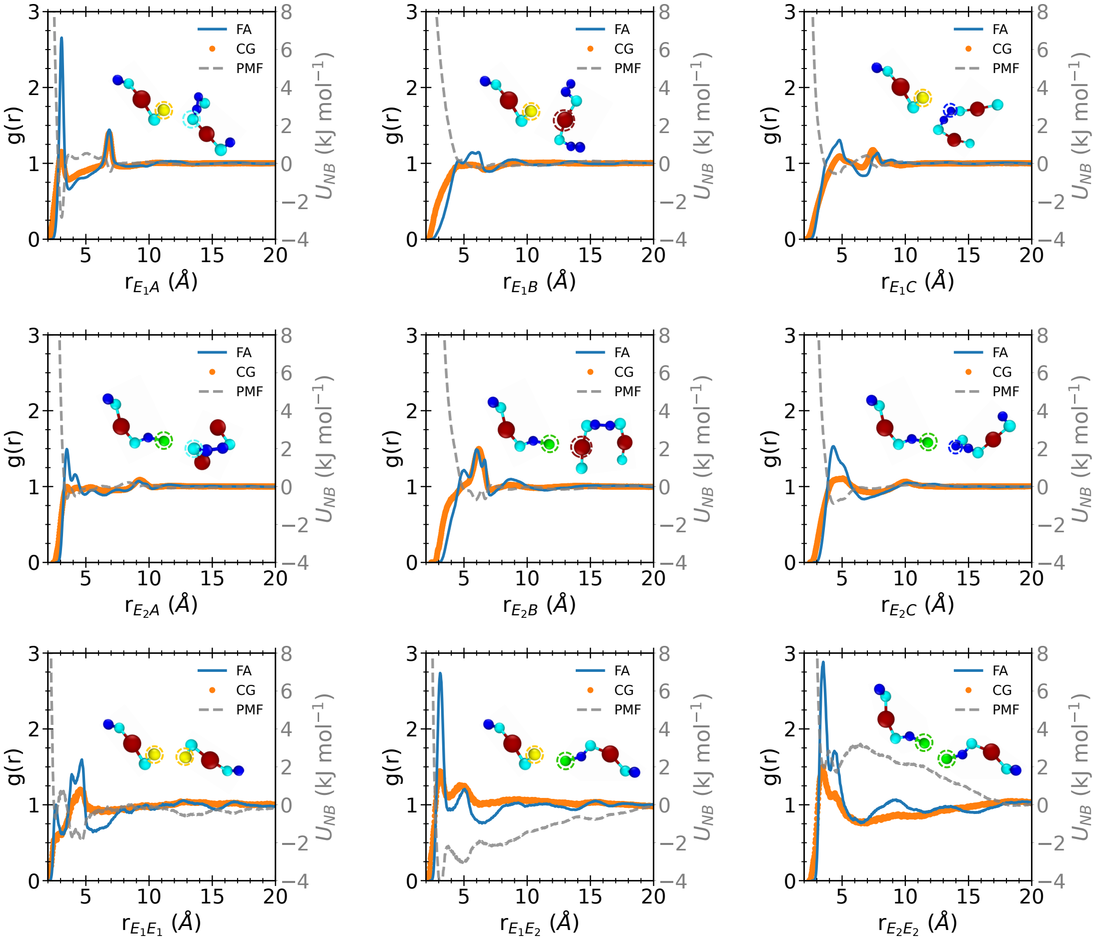

Figure S35: Averaged probability distribution functions for radial distribution functions of the 10**RMU** system obtained using mapping scheme C on QMD-FF at FA (blue lines) and CG level (orange circles). In all panels, the corresponding CG effective potentials are displayed with grey dashed lines. Only distributions involving terminal CG beads are included.

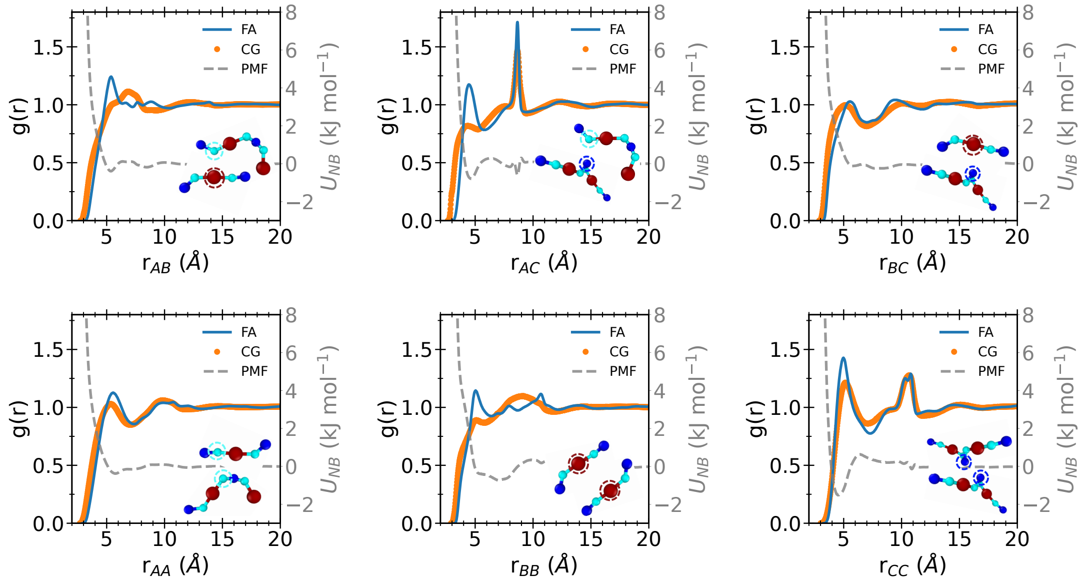

Figure S36: Averaged probability distribution functions for radial distribution functions of the 10**RMU** system obtained using mapping scheme A on Tr-FF at FA (blue lines) and CG level (orange circles). In all panels, the corresponding CG effective potentials are displayed with grey dashed lines. Only distributions involving inner CG beads are included.

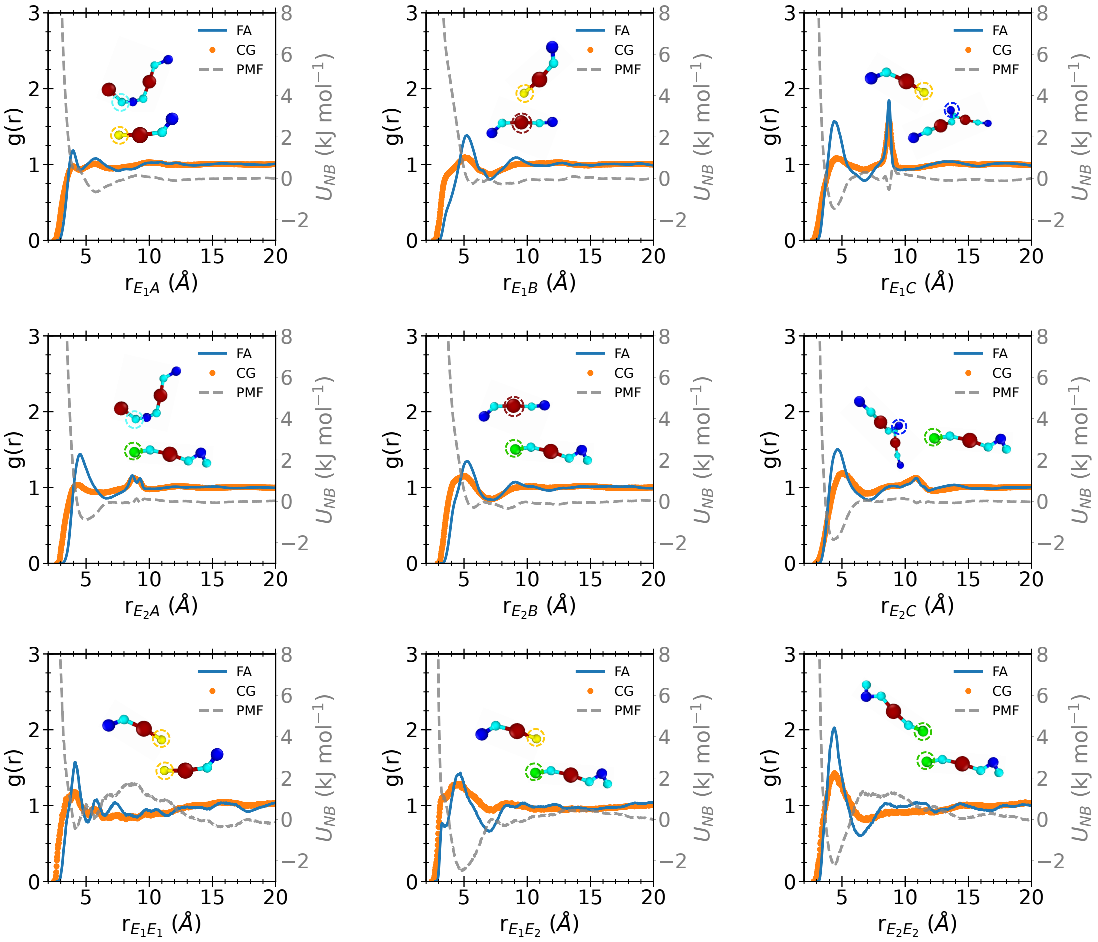

Figure S37: Averaged probability distribution functions for radial distribution functions of the 10**RMU** system obtained using mapping scheme A on Tr-FF at FA (blue lines) and CG level (orange circles). In all panels, the corresponding CG effective potentials are displayed with grey dashed lines. Only distributions involving terminal CG beads are included.

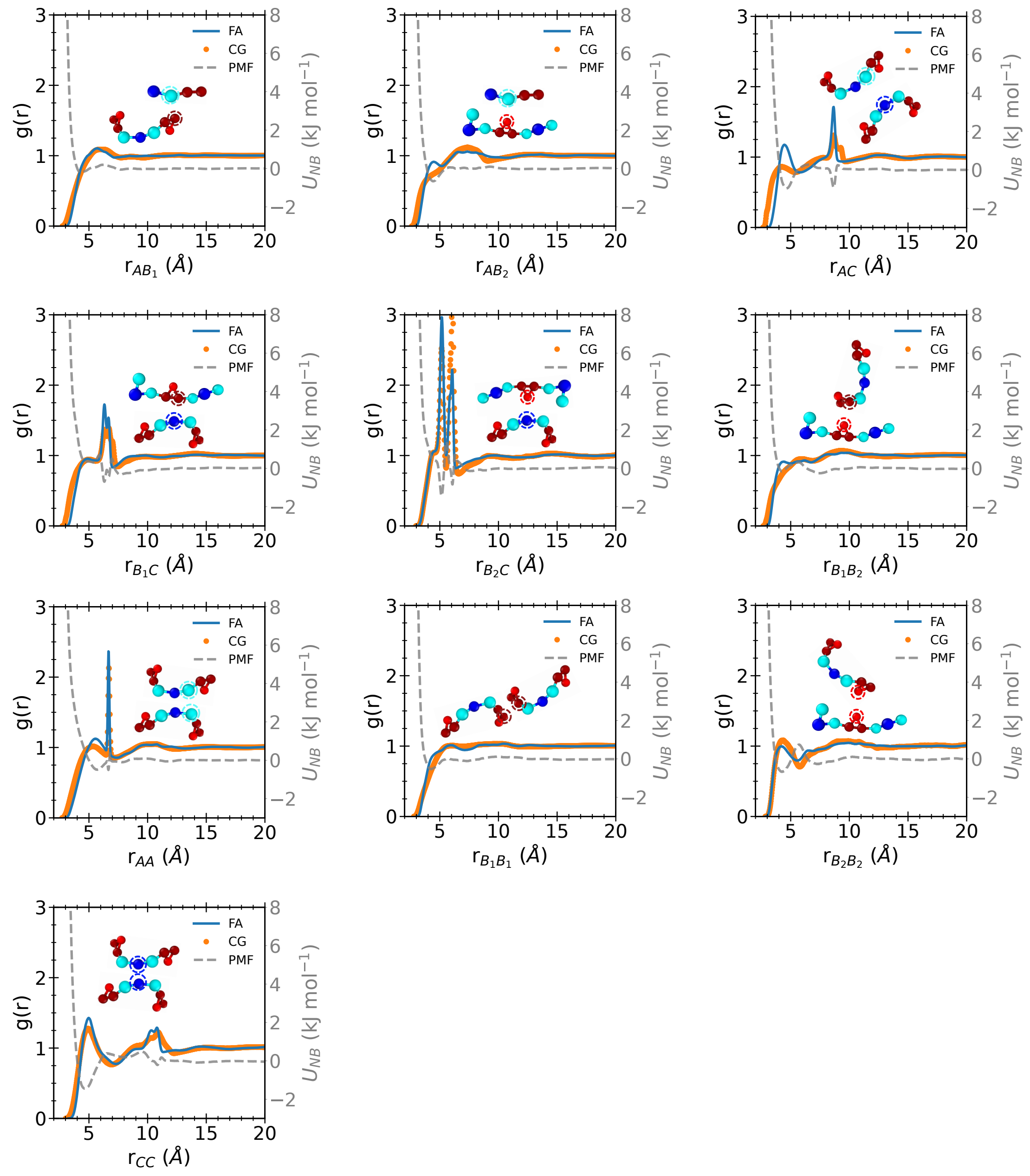

Figure S38: Averaged probability distribution functions for radial distribution functions of the 10**RMU** system obtained using mapping scheme B on Tr-FF at FA (blue lines) and CG level (orange circles). In all panels, the corresponding CG effective potentials are displayed with grey dashed lines. Only distributions involving inner CG beads are included.

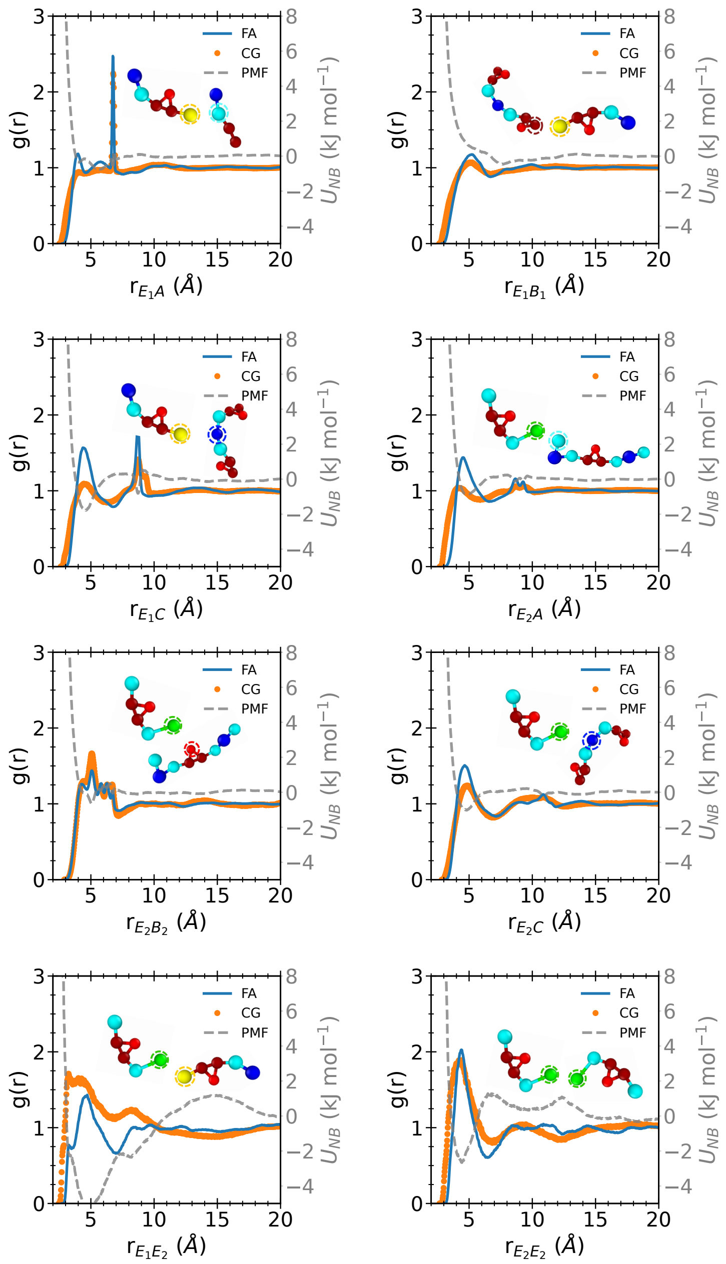

Figure S39: Averaged probability distribution functions for radial distribution functions of the 10**RMU** system obtained using mapping scheme B on Tr-FF at FA (blue lines) and CG level (orange circles). In all panels, the corresponding CG effective potentials are displayed with grey dashed lines. Only distributions involving terminal CG beads are included.

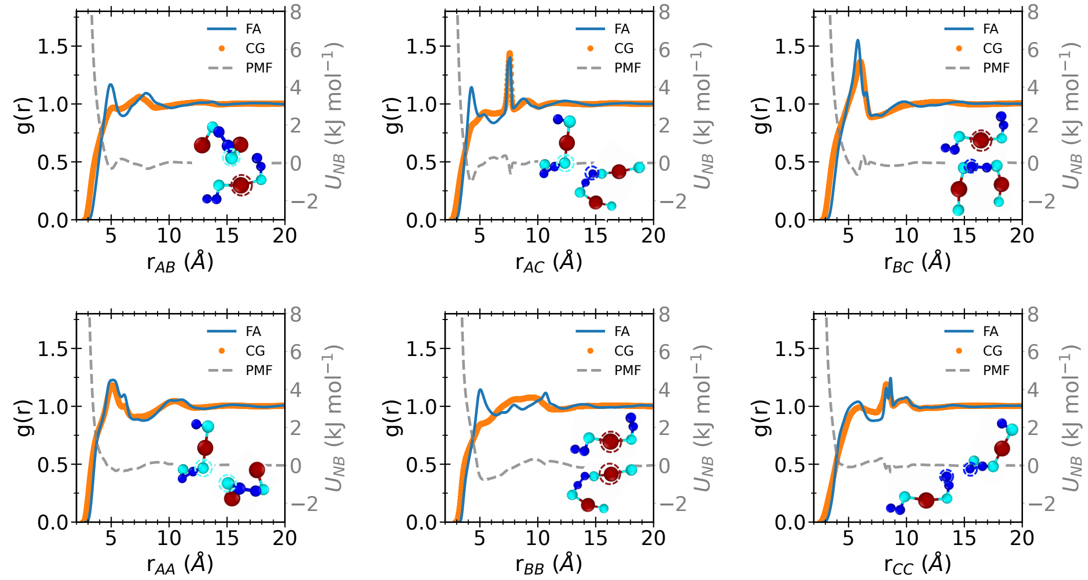

Figure S40: Averaged probability distribution functions for radial distribution functions of the 10**RMU** system obtained using mapping scheme C on Tr-FF at FA (blue lines) and CG level (orange circles). In all panels, the corresponding CG effective potentials are displayed with grey dashed lines. Only distributions involving inner CG beads are included.

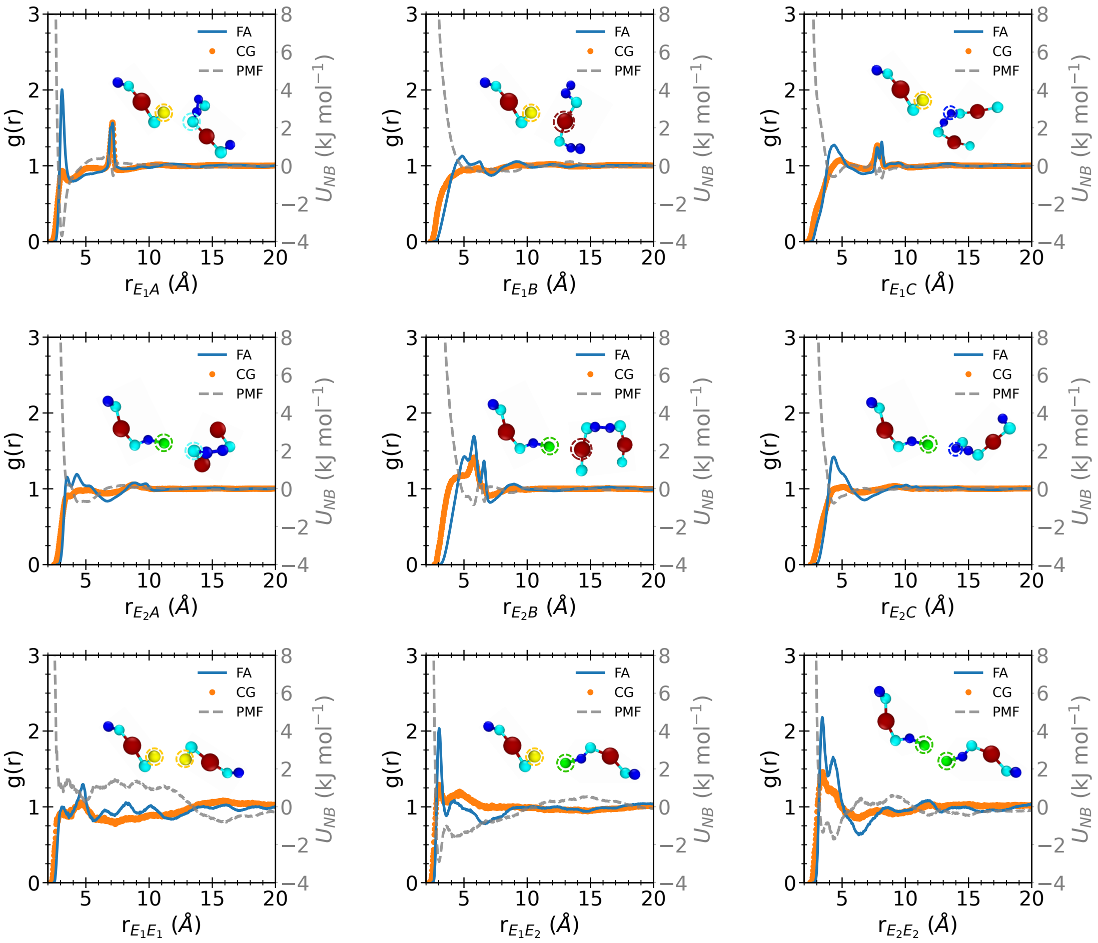

Figure S41: Averaged probability distribution functions for radial distribution functions of the 10**RMU** system obtained using mapping scheme C on Tr-FF at FA (blue lines) and CG level (orange circles). In all panels, the corresponding CG effective potentials are displayed with grey dashed lines. Only distributions involving terminal CG beads are included.

All in all, the agreement of the CG RDFs with the FA RDFs is very good for all mapping schemes, proving that the IBI method can discern the main differences among similar mapping schemes and reference trajectories obtained with distinct FA-FFs.

S6.2.5 Global structural properties

The CG radius of gyration $P(R_g)$ and end-to-end distance distribution $P(R_{ee})$ were computed for the 10**RMU** system considering the three mapping schemes and using either the Tr-FF or QMD-FF derived CG-FFs, and compared in Figure S42 and S43 with those retrieved from the FAMD runs. The R_g distributions extracted from CGMD closely follows the atomistic distributions for all models. The $\langle R_g \rangle$ value obtained with the CG-Tr models fall very close to the value of 13.2 ± 3.8 Å reported previously by Keffer *et al.* [30].

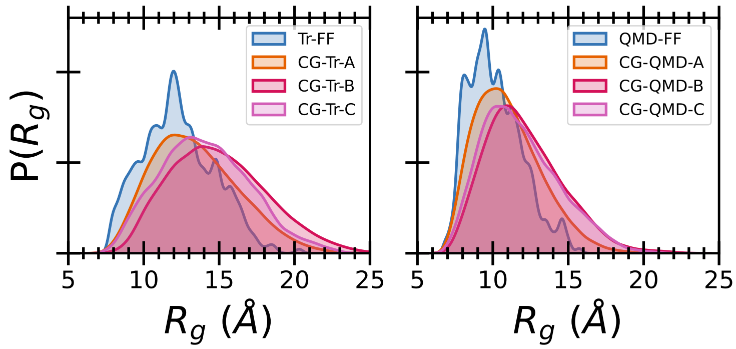

Figure S42: Comparison between FA and CG distributions of the radius of gyration, P(R_g), of the 10**RMU** system obtained using the different mapping schemes. Parametrization results for both CG-Tr (left) and CG-QMD (right) are shown for comparison.

In both cases, albeit $\langle R_{ee} \rangle$ is very close to the atomistic value, the width of the CG distributions significantly overstimates the FA one, extending more than double the full width at half maximum (FWHM). Furthermore, for the QMD CG models, we observe slight differences in the distribution width and average value among the mapping schemes. In particular, mapping scheme B displays a very long tail, clearly visible also for CG-Tr-B model. Such an effect might be due to a combination of sampling issues the atomistic scale and end group effects in the CG model. In fact, even though a large number of atomistic trajectories have been sampled, it may not have been sufficient to populate the states at large R_{ee}. Goujon *et al.* analyze the effect of this mismatch in great detail [31]. Besides, due to the relatively short length of the

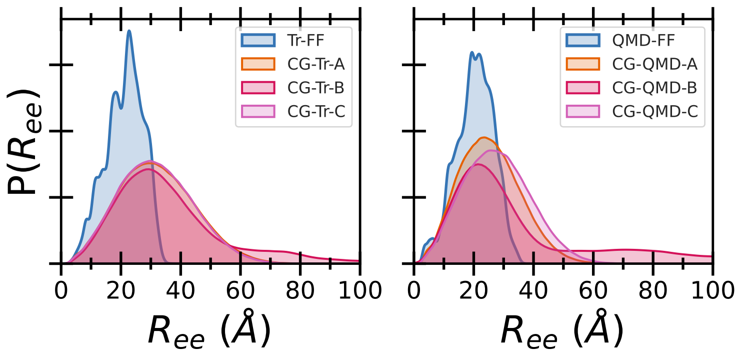

Figure S43: Comparison between FA and CG distributions of the end-to-end distances, P(R_{ee}), of the 10**RMU** system obtained using the different mapping schemes. Parametrization results for both CG-Tr (left) and CG-QMD (right) are shown for comparison.

10**RMU** chains, the terminal beads will present significant excluded volume effect, which in turn lead to the overestimation of the polymer density. A similar behavior has been observed previously for polystyrene [32]. In any case, CG-Tr models predict more rigid chains than CG-QMD (larger R_{ee}), in line with the atomistic picture.

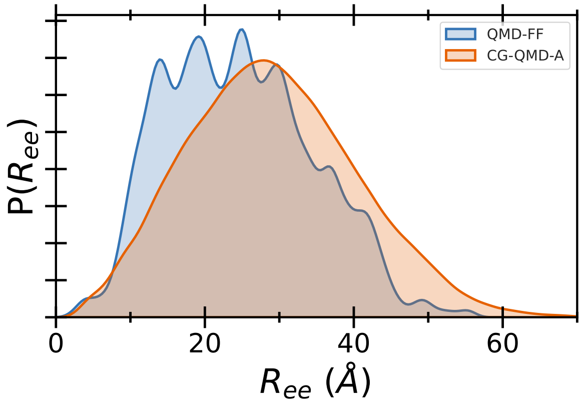

Figure S44: Comparison between FA and CG distributions of the end-to-end distance, $P(R_{ee})$, of the 400 × 10**RMU** system obtained using mapping scheme A. Only the parametrization results for CG-QMD is shown.

As a back-up case, we have rerun FAMD and CGMD simulations on a larger system consisting of 400 chains. As displayed in Figure S44, the resulting FA distribution is more spread and the agreement with the CG predictions improves considerably.

To understand the impact of the dihedrals on the global structure, we performed CGMD simulations without considering the torsion potentials for scheme A, and compared it with the full CG-FF. Figure S45 shows the R_{ee} distribution for both cases. As can be observed, when

dihedral interactions are neglected, the chains become more flexible and extended. Dihedral interactions are responsible for increasing chain stiffness by restricting free rotation. This remarks that 1−4 bonded interactions may still be important depending on the polymer and degree of resolution of CG model, and its exclusion has to be properly justified.

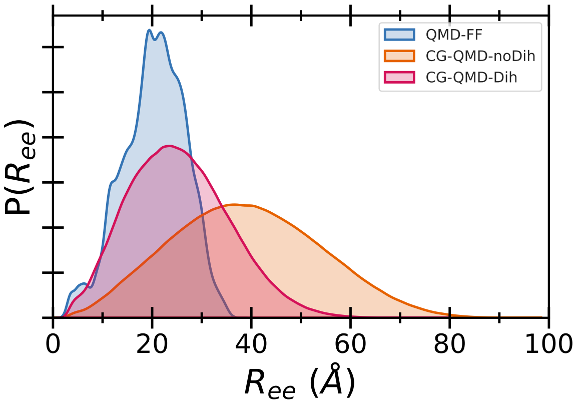

Figure S45: Comparison between FA and CG distributions of the end-to-end distance, $P(R_{ee})$, of the 10**RMU** system obtained using mapping scheme A. Parametrization results for CG-QMD with and without dihedral interactions are shown for comparison.

References

[1] Rezáč, J.; Hobza, P. Benchmark Calculations of Interaction Energies in Noncovalent Complexes and Their Applications. *Chem. Rev.* **2016**, *116*, 5038–5071.

[2] Prampolini, G.; Livotto, P. R.; Cacelli, I. Accuracy of Quantum Mechanically Derived Force-Fields Parameterized from Dispersion-Corrected DFT Data: The Benzene Dimer as a Prototype for Aromatic Interactions. *J. Chem. Theory Comput.* **2015**, *11*, 5182–96.

[3] Jacobs, M.; Greff Da Silveira, L.; Prampolini, G.; Livotto, P. R.; Cacelli, I. Interaction Energy Landscapes of Aromatic Heterocycles through a Reliable yet Affordable Computational Approach *J. Chem. Theory Comput.* **2018**, *14*, 543–556.

[4] Cacelli, I.; Lipparini, F.; Greff da Silveira, L.; Jacobs, M.; Livotto, P. R.; Prampolini, G. Accurate interaction energies by spin component scaled Möller-Plesset second order perturbation theory calculations with optimized basis sets (SCS-MP2^{mod}): Development and application to aromatic heterocycles *J. Chem. Phys.* **2019**, *150*, 234113.

[5] Halkier, A.; Helgaker, T.; Jorgensen, P.; Klopper, W.; Koch, H.; Olsen, J.; Wilson, A. Basis-set Convergence in Correlated Calculations on Ne, N_2 and H_2O *Chem. Phys. Lett.* **1998**, *286*, 243.

[6] Sherrill, C. D.; Takatani, T.; Hohenstein, E. G. An Assessment of Theoretical Methods for Nonbonded Interactions: Comparison to Complete Basis Set Limit Coupled-Cluster Potential Energy Curves for the Benzene Dimer, the Methane Dimer, Benzene-Methane, and Benzene-H_2S *J. Phys. Chem. A* **2009**, *113*, 10146–10159.

[7] Frisch, M. J.; Trucks, G. W.; Schlegel, H. B.; Scuseria, G. E.; Robb, M. A.; Cheeseman, J. R.; Scalmani, G.; Barone, V.; Petersson, G. A.; Nakatsuji, H.; Li, X.; Caricato, M.; Marenich, A. V.; Bloino, J.; Janesko, B. G.; Gomperts, R.; Mennucci, B.; Hratchian, H. P.; Ortiz, J. V.; Izmaylov, A. F.; Sonnenberg, J. L.; Williams-Young, D.; Ding, F.; Lipparini, F.; Egidi, F.; Goings, J.; Peng, B.; Petrone, A.; Henderson, T.; Ranasinghe, D.; Zakrzewski, V. G.; Gao, J.; Rega, N.; Zheng, G.; Liang, W.; Hada, M.; Ehara, M.;

Toyota, K.; Fukuda, R.; Hasegawa, J.; Ishida, M.; Nakajima, T.; Honda, Y.; Kitao, O.; Nakai, H.; Vreven, T.; Throssell, K.; Montgomery, J. A., Jr.; Peralta, J. E.; Ogliaro, F.; Bearpark, M. J.; Heyd, J. J.; Brothers, E. N.; Kudin, K. N.; Staroverov, V. N.; Keith, T. A.; Kobayashi, R.; Normand, J.; Raghavachari, K.; Rendell, A. P.; Burant, J. C.; Iyengar, S. S.; Tomasi, J.; Cossi, M.; Millam, J. M.; Klene, M.; Adamo, C.; Cammi, R.; Ochterski, J. W.; Martin, R. L.; Morokuma, K.; Farkas, O.; Foresman, J. B.; Fox, D. J.; Gaussian˜16 Revision D.02; 2016; Gaussian Inc. Wallingford CT.

[8] Boys, S.; Bernardi, F. The Calculation of Small Molecular Interactions by the Differences of Separate Total Energies. Some Procedures with Reduced Errors *Mol. Phys.* **1970**, *19*, 553–566.

[9] Grimme, S.; Ehrlic, S.; Goerigk, L. Effect of the Damping Function in Dispersion Corrected Density Functional Theory *J. Comp. Chem.* **2011**, *32*, 1456–65.

[10] Cacelli, I.; Prampolini, G. Parametrization and Validation of Intramolecular Force Fields Derived from DFT Calculations *J. Chem. Theory Comput.* **2007**, *3*, 1803–1817.

[11] Giannini, S.; Martinez, P. M.; Semmeq, A.; Galvez, J. P.; Piras, A.; Landi, A.; Padula, D.; Vilhena, J. G.; Cerezo, J.; Prampolini, G. JOYCE3.0: A General Protocol for the Specific Parametrization of Accurate Intramolecular Quantum Mechanically Derived Force Fields *J. Chem. Theory Comput.* **2025**, *21*, 3156–3175.

[12] Martinez, P. M.; Piras, A.; Cerezo, J.; Galvez, J. P.; Giannini, S.; Landi, A.; Padula, D.; Semmeq, A.; Vilhena, J. G.; Prampolini, G.; JOYCE website, visit: `https://joyce-documentation.gitlab.io/`; 2024.

[13] Cerezo, J.; Prampolini, G.; Cacelli, I. Developing accurate intramolecular force fields for conjugated systems through explicit coupling terms *Theor. Chem. Accounts* **2018**, *137*, 80.

[14] Cacelli, I.; Cimoli, A.; Livotto, P. R.; Prampolini, G. An Automated Approach for the Parameterization of Accurate Intermolecular Force-Fields: Pyridine as a Case Study. *J. Comp. Chem.* **2012**, *33*, 1055.

[15] Vilhena, J. G.; Greff da Silveira, L.; Livotto, P. R.; Cacelli, I.; Prampolini, G. Automated Parameterization of Quantum Mechanically Derived Force Fields for Soft Materials and Complex Fluids: Development and Validation *J. Chem. Theory Comput.* **2021**, *17*, 4449–4464.

[16] Greff Da Silveira, L.; Jacobs, M.; Prampolini, G.; Livotto, P. R.; Cacelli, I. Development and Validation of Quantum Mechanically Derived Force-Fields: Thermodynamic, Structural, and Vibrational Properties of Aromatic Heterocycles *J. Chem. Theory Comput.* **2018**, *14*, 4884–4900.

[17] Amovilli, C.; Cacelli, I.; Campanile, S.; Prampolini, G. Calculation of the Intermolecular Energy of Large Molecules by a Fragmentation Scheme: Application to the 4-*n*-Pentyl-4′-Cyanobiphenyl (5CB) Dimer *J. Chem. Phys.* **2002**, *117*, 3003.

[18] Cacelli, I.; Cinacchi, G.; Prampolini, G.; Tani, A. Computer Simulation of Solid and Liquid Benzene with an Atomistic Interaction Potential Derived from Ab Initio Calculations. *J. Am. Chem. Soc.* **2004**, *126*, 14278–86.

[19] Prampolini, G.; A., C.; Cacelli, I.; PICKY3.0, a Fortran 77 code for inter-molecular force field parameterization, available free of at http://www.iccom.cnr.it/en/picky-en/, last consulted May 2022; 2020.

[20] Jorgensen, W. L.; Tirado-Rives, J. Potential Energy Functions for Atomic-Level Simulations of Water and Organic and Biomolecular Systems. *Proc. Natl. Acad. Sci. USA* **2005**, *102*, 6665–70.

[21] Dodda, L. S.; Cabeza de Vaca, I.; Tirado-Rives, J.; Jorgensen, W. L. LigParGen web server: an automatic OPLS-AA parameter generator for organic ligands *Nucleic Acids Res.* **2017**, *45*, W331–W336.

[22] Abraham, M. J.; Murtola, T.; Schulz, R.; Páll, S.; Smith, J. C.; Hess, B.; Lindahl, E. GROMACS: High performance molecular simulations through multi-level parallelism from laptops to supercomputers *SoftwareX* **2015**, *1-2*, 19 – 25.

[23] Lightfoot, J.; Buchard, A.; Castro-Dominguez, B.; Parker, S. Comparative Study of Oxygen Diffusion in Polyethylene Terephthalate and Polyethylene Furanoate ff Using Molecular Modeling: Computational Insights into the Mechanism for Gas Transport in Bulk Polymer Systems *Macromol.* **2022**, *55*, 498–510.

[24] Reith, D.; Meyer, H.; Müller-Plathe, F. Mapping Atomistic to Coarse-Grained Polymer Models Using Automatic Simplex Optimization To Fit Structural Properties *Macromol.* **2001**, *34*, 2335–2345.

[25] Müller-Plathe, F. Coarse-Graining in Polymer Simulation: From the Atomistic to the Mesoscopic Scale and Back *ChemPhysChem* **2002**, *3*, 754–769.

[26] Reith, D.; Pütz, M.; Müller-Plathe, F. Deriving effective mesoscale potentials from atomistic simulations *J. Comp. Chem.* **2003**, *24*, 1624–1636.

[27] Henderson, R. A uniqueness theorem for fluid pair correlation functions *Physics Letters A* **1974**, *49*, 197–198.

[28] Eslami, H.; Karimi-Varzaneh, H. A.; Müller-Plathe, F. Coarse-Grained Computer Simulation of Nanoconfined Polyamide-6,6 *Macromolecules* **2011**, *44*, 3117–3128.

[29] Thompson, A. P.; Aktulga, H. M.; Berger, R.; Bolintineanu, D. S.; Brown, W. M.; Crozier, P. S.; in 't Veld, P. J.; Kohlmeyer, A.; Moore, S. G.; Nguyen, T. D.; Shan, R.; Stevens, M. J.; Tranchida, J.; Trott, C.; Plimpton, S. J. LAMMPS - a flexible simulation tool for particle-based materials modeling at the atomic, meso, and continuum scales *Comp. Phys. Comm.* **2022**, *271*, 108171.

[30] Wang, Q.; Keffer, D. J.; Nicholson, D. M.; Thomas, J. B. Coarse-Grained Molecular Dynamics Simulation of Polyethylene Terephthalate (PET) *Macromol.* **2010**, *43*, 10722–10734.

[31] Nkepsu Mbitou, R. L.; Goujon, F.; Dequidt, A.; Latour, B.; Devémy, J.; Blaak, R.; Martzel, N.; Emeriau-Viard, C.; Tchoufag, J.; Garruchet, S.; Munch, E.; Hauret, P.; Mal-

freyt, P. Consistent and Transferable Force Fields for Statistical Copolymer Systems at the Mesoscale *J. Chem. Theory Comput.* **2022**, *18*, 6940–6951.

[32] Bayramoglu, B.; Faller, R. Coarse-Grained Modeling of Polystyrene in Various Environments by Iterative Boltzmann Inversion *Macromolecules* **2012**, *45*, 9205–9219.
